# Design, synthesis and evaluation of 2, 6, 8-substituted Imidazopyridine derivatives as potent PI3K*α* inhibitors

**DOI:** 10.1080/14756366.2022.2155638

**Published:** 2023-01-17

**Authors:** Rui Chen, Zhongyuan Wang, Lijie Sima, Hu Cheng, Bilan Luo, Jianta Wang, Bing Guo, Shunyi Mao, Zhixu Zhou, Jingang Peng, Lei Tang, Xinfu Liu, Weike Liao

**Affiliations:** a Guizhou Provincial Engineering Technology Research Center for Chemical Drug R&D, Guizhou Medical University, Guiyang, China; b Department of Pharmacy, Guizhou Provincial People’s Hospital, Guiyang, China; c Department of Hematology and Oncology, The Affiliated Shaoyang Hospital, Hengyang Medical School, University of South China (Shaoyang Central Hospital), China; d Guizhou Provincial Key Laboratory of Pathogenesis and Drug Research on Common Chronic Diseases, Guizhou Medical University, Guiyang, China; e School of Pharmaceutical Sciences, Guizhou University, Guiyang, China

**Keywords:** PI3Kα, synthesis, Imidazo[12-a]pyridine derivatives, antitumor activity

## Abstract

Inhibition of PI3K pathway has become a desirable strategy for cancer treatment. In this work, a series of 2, 6, 8-substituted Imidazo[1,2-a]pyridine derivatives were designed and screened for their activities against PI3K*α* and a panel of PI3K*α*-addicted cancer cells. Among them, compound **35** was identified as a PI3K*α* inhibitor with nanomolar potency as well as acceptable antiproliferative activity. Flow cytometry analysis confirmed **35** induced cell cycle arrest and apoptosis in T47D cells. In addition, it also showed desirable *in vitro* ADME properties. The design, synthesis, and SAR exploration of **35** are described within.

## Introduction

Malignant tumours have always been one of the major diseases threatening human health. At present, the commonly used treatment methods for cancer are inseparable from the support of chemotherapy drugs^1^. Therefore, it is of great significance to find anti-tumour drugs with high efficiency and low side effects^2^.

Phosphatidylinositol-3-kinases (PI3Ks) are a family of lipid kinases that are responsible for catalysing the phosphorylation of inositol phospholipids to produce PIP3 (phosphatidylinositol triphosphate). This phosphorylation process results in recruiting cytosolic signalling enzymes such as Akt to the plasma membrane, triggering cell growth, proliferation, differentiation, and motility^3–5^. Aberrant activation of PI3K as well as its downstream effectors including Akt and mTOR has been linked to numerous forms of cancer including lymphatic tumours, breast, lung, brain, ovarian, melanoma, and prostate cancers^6–8^. Moreover, the negative regulator PTEN^9^ which dephosphorylates PIP3 to PIP2, is often inactivated in many cancer types, leading to elevated levels of PIP3 and increased tumour survival^3^. With distinct sequence homology and substrate preferences, the eight known PI3K family members are divided into classes I, II, and III. The most extensively studied Class I PI3Ks are further subdivided into Class IA (*α*, *β*, and *δ*) and Class IB (PI3K*γ*), based upon the types of regulatory subunits and the catalytic domains to which they bind in the active heterodimeric forms. The former Class IA PI3Ks are activated through tyrosine kinase signalling, whereas the sole PI3K*γ* is mostly activated through GPCRs^10^.

**Figure 1.**
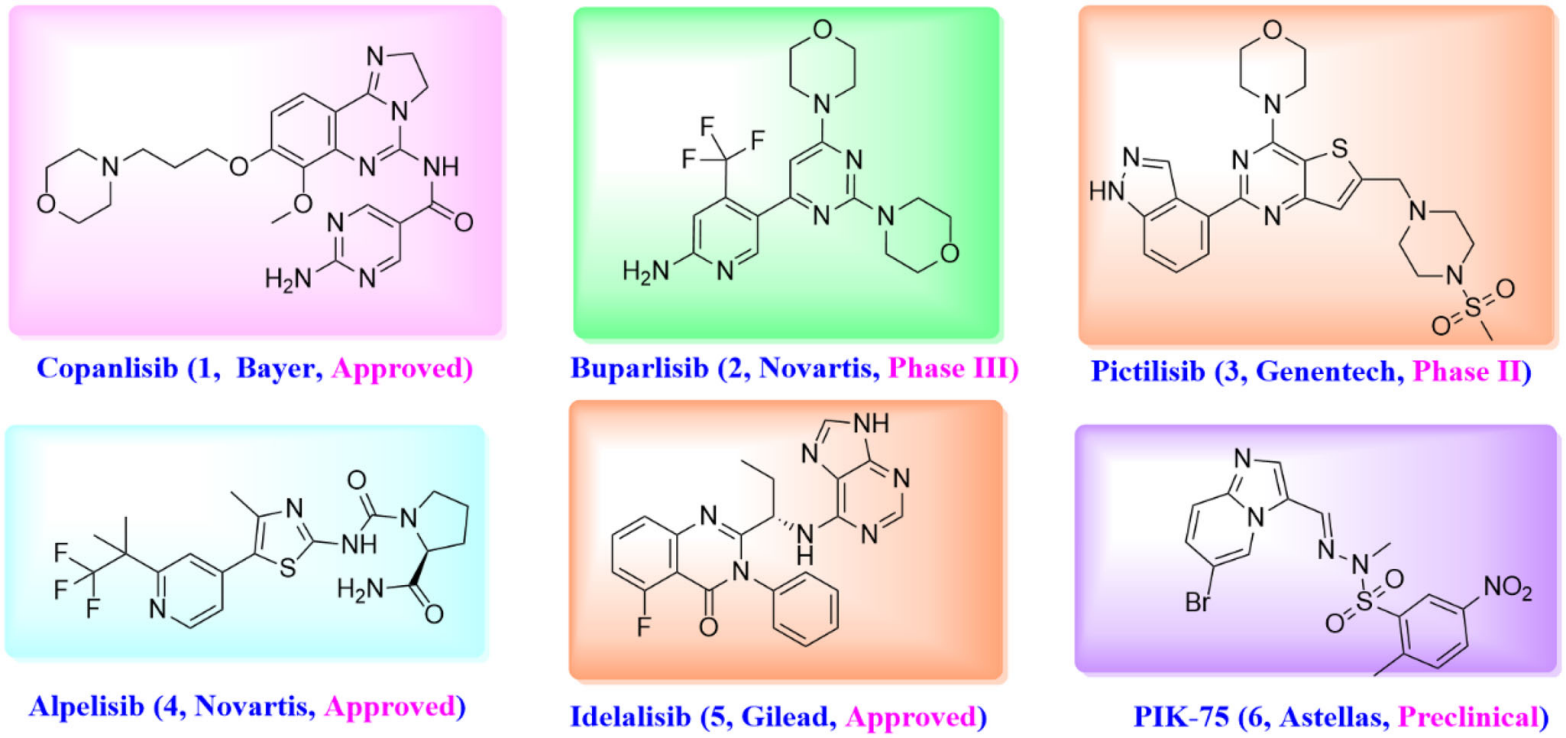
Representative structures of reported PI3K inhibitors.

**Figure 2.**
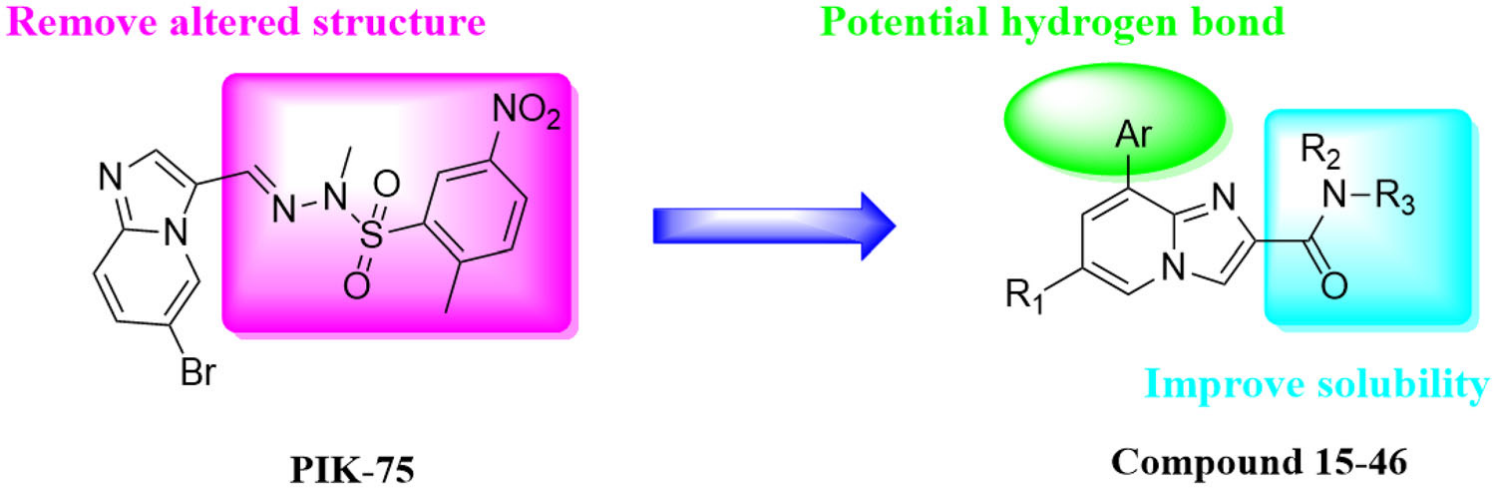
The design of novel Imidazo[1,2-a]pyridine derivatives based on PIK-75.

While there is growing evidence that small molecule inhibition of PI3Ks as an attractive strategy for oncology indication^11^^,^^12^. Several small molecule inhibitors have been approved by FDA for use in patients or under active clinical development, including pan-PI3K inhibitors Copanlisib (**1**)^13^^,^^14^, Buparlisib (**2**)^15^, and GDC-0941 (**3**)^16^, *α*-specific PI3K inhibitor Alpelisib (**4**)^17^^,^^18^, PI3K*δ*-selective inhibitor Idelalisib (**5**)^19^, and so on (Figure 1). The common pharmacophoric features of reported PI3K*α* inhibitors are summarised as follows: First, the morpholine or heterocycle ring forms an important hydrogen bond with Val851 in the hinge region. Another hydrogen bond can be found between aromatic ring or lipophilic side chain with Tyr836, Asp810 and/or Lys802 at the affinity pocket. Furthermore, some derivatives extend to the solvent exposed region to form additional interaction with the surrounding amino acids as well as improve the druggability of designed compounds. Among them, PIK-75 (**6**) is an Imidazo[1,2-a]pyridine derivative that shows good selectivity for PI3K*α* over the other related Class I PI3Ks as well as desirable activity in a human cancer xenograft model^20^. However, the SAR explorations on PIK-75 are focussed on either replacing the Imidazo[1,2-a]pyridine ring with other heterocycles or modifying the sulfonohydrazone side chains^21^, modifications on C-2 or C-8 position have barely been reported. Considering the sulfonohydrazide group as an alert structure, we were interested in removing this group, and introducing amide and substituted aryl group to the 2-, and 8-position of Imidazo[1,2-a]pyridine ring to improve the solubility as well as provide hydrogen binding group that was essential for PI3K*α* activity (Figure 2).

In this manuscript, we communicate the discovery and optimisation of a series of Imidazo[1,2-a]pyridine derivatives as PI3K*α* selective inhibitors and their potential application in the treatment of cancers. The efforts leading to the discovery of the PI3K*α*-specific inhibitor **35** are described herein.

## Results and discussion

### Chemistry

**Scheme 1.**
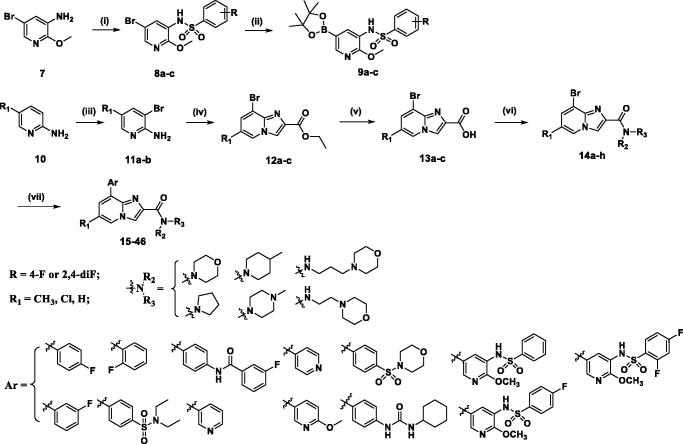
Reagents and conditions: (i) substituted benzene-1-sulfonyl chloride, pyridine, room temperature, overnight; (ii) (BPin)_2_, PdCl_2_(dppf), KOAc, dioxane, 100 °C, 8 h; (iii) NBS, CH_3_CN, 0 °C, 4 h; (iv) ethyl bromopyruvate, EtOH, 80 °C, 4 h; (v) NaOH, EtOH, 80 °C, 3 h; (vi) amines, HBTU, Et_3_N, DMF, rt, overnight; (vii) aryl boronic acid or **9a-c**, Pd(PPh_3_)_4_, K_2_CO_3_, 1,4-Dioxane/H_2_O, 100 °C, overnight.

As depicted in Scheme 1, a simple five-step synthetic sequence was used for the preparation of compounds described herein. Firstly, 2-aminopyridine derivatives and *N*-Bromosuccinimide (NBS) undergo electrophilic aromatic substitution reaction in the presence of DMF to obtain 2-amino-3-bromo-pyridine derivatives (**11a-b**), **11a-b** and commercially available 3-bromopyridin-2-amine were then cyclized with ethyl 3-bromopyruvate to give the ethyl 8-bromo-Imidazo[1,2-a]pyridine-2-carboxylate derivatives **(12a-c**). Intermediates **12a-c** were further hydrolysed by NaOH to offer the corresponding carboxylic acids **(13a-c)**. Subsequently, the key intermediates **14a-h** were synthesised through amidation reaction of **13a-c** with various amines and coupling reagent HBTU in DMF. Finally, compounds **14a-h** undergo Suzuki-Miyaura reaction with the corresponding boronic acids or esters (**9a-c**) to obtain the corresponding target products **15–46**. The chemical structures of the target compounds were confirmed with HRMS, ^1^H NMR, and ^13^C NMR.

### Biological evaluation

#### PI3Kα inhibition and SARs

To screen the inhibitory activity of the target compounds against PI3K*α*, the Kinase-Glo™ assay with ATP at 25 μM was utilised with PIK-75 as positive control^22^. We chose to explore the SARs at three positions on the Imidazo[1,2-a]pyridine core. To start with, a set of 8-position modifications were prepared while keeping the morpholinyl amide substituent on 2-position fixed, since it allowed quick structure − activity profiling via coupling reactions of bromide with aryl borates or boronic acids. The results are summarised in Table 1. Fluoro substituted phenyl was first incorporated, through scanning the 2, 3, 4-position of phenyl, it was clear that the ortho-position was not well tolerated (**15**, **16** vs **17**), with only 14.1% inhibitory rate at 10 μM. Moreover, meta-fluoro substituted analog **16** showed a slightly enhanced PI3K*α* activity compared to para-fluoro substituted analog **15**. A similar trend can be observed for compound **23** vs **22**. Replacement of fluorophenyl with 3-pyridinyl **20**, 6-methoxy-3-pyridinyl **21** or 4-pyridinyl **30** did not increase the activity (**20**, **21** vs **16**; **30** vs **29**). We then turned our attention back to the phenyl substituents. Changing the fluoro group of compound **15** to amide **18**, sulphonamide **19**, or urea **33** led to an increase in potency, with the inhibitory rate range from 44.5 to 58.8, a similar trend can be found both at the pyrrolidinyl amide (**26–28**) and *N*-methylpiperidinyl series (**29–33**).

**Table 1.** *In vitro* enzyme inhibitory activity of target compounds on PI3K*α* at 10 μM.
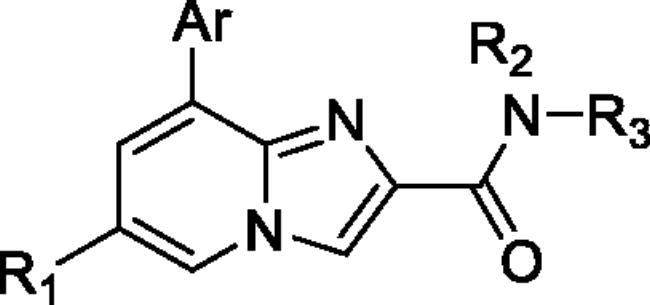

Compd.	R_1_	-NR_2_R_3_	Ar	% Inhibition at 10 μM^a^
**15**	-CH_3_	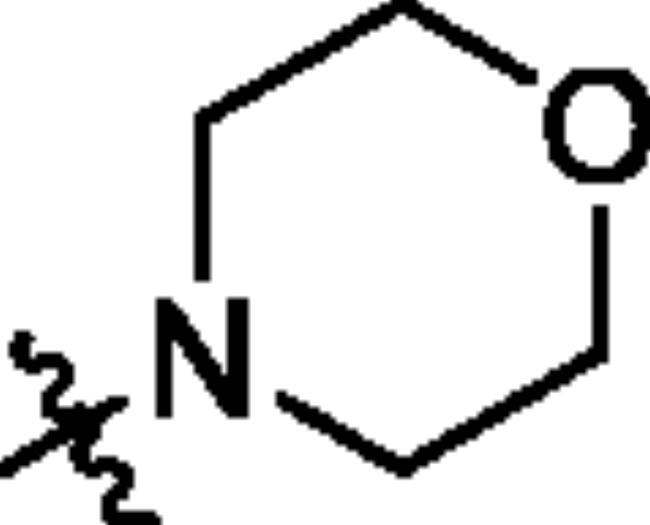	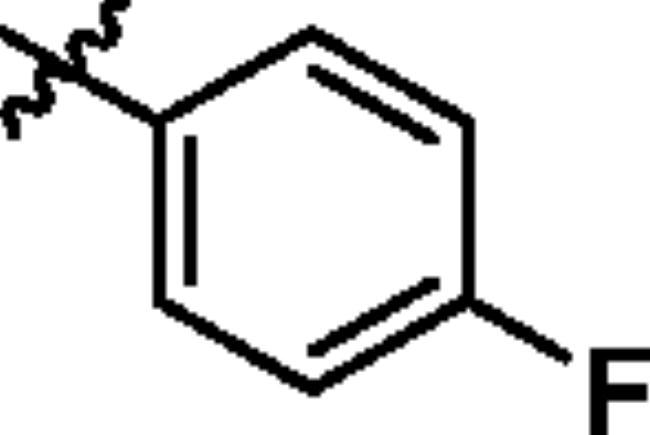	31.9 ± 2.9
**16**	-CH_3_	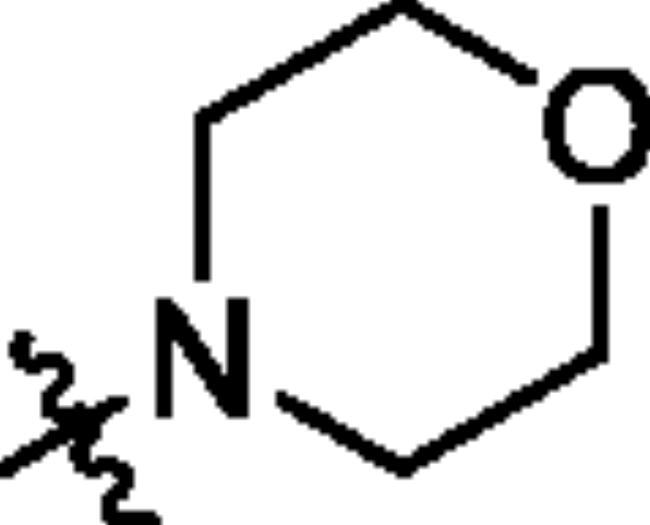	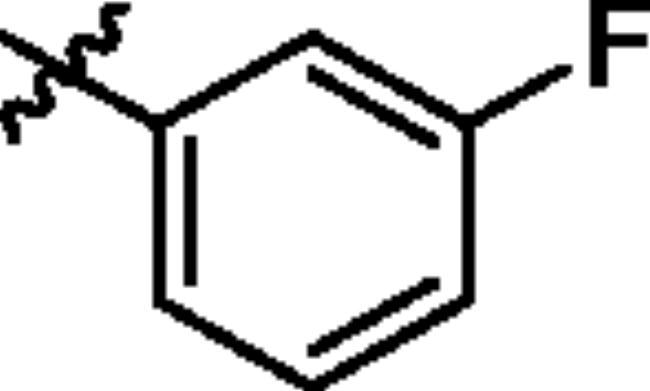	38.2 ± 3.6
**17**	-CH_3_	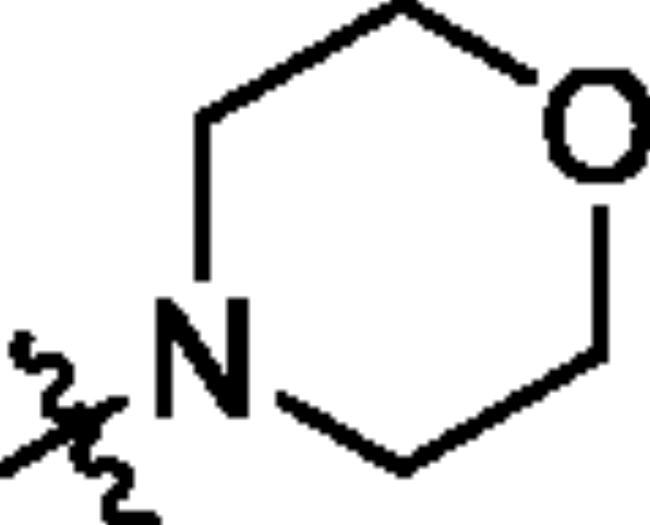	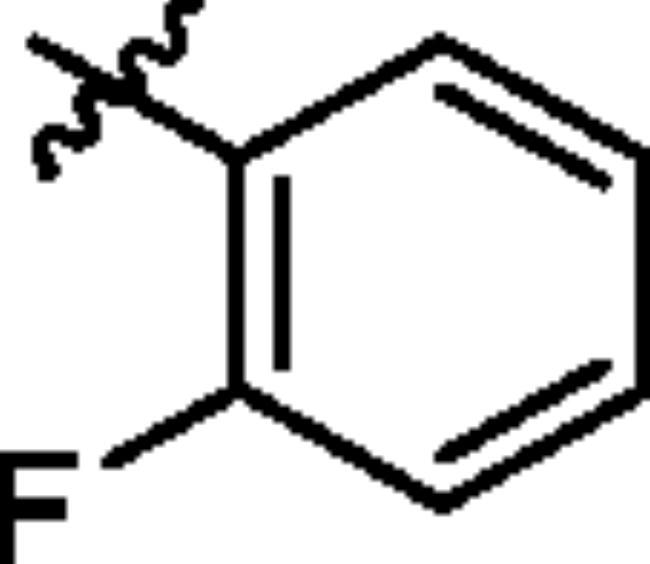	14.1 ± 2.7
**18**	-CH_3_	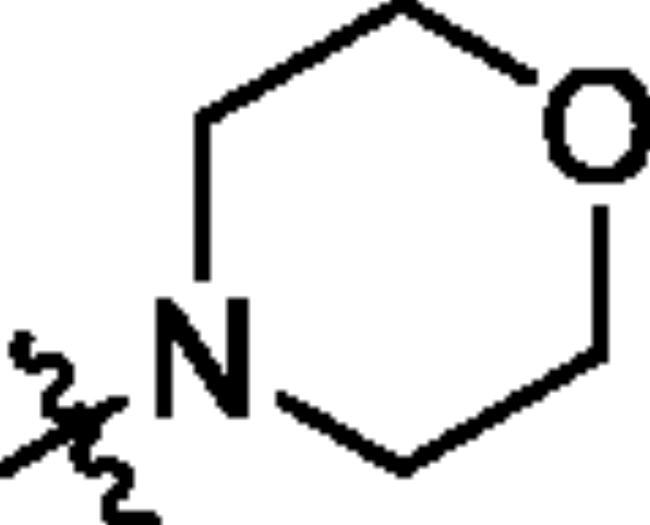	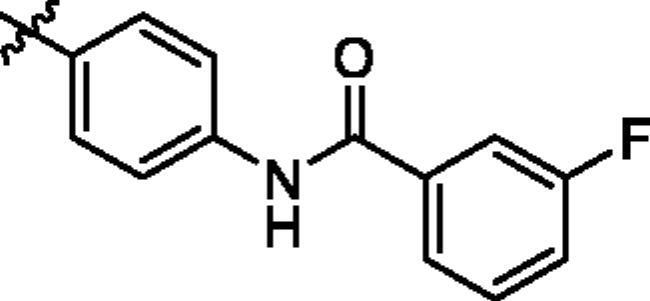	47.6 ± 3.9
**19**	-CH_3_	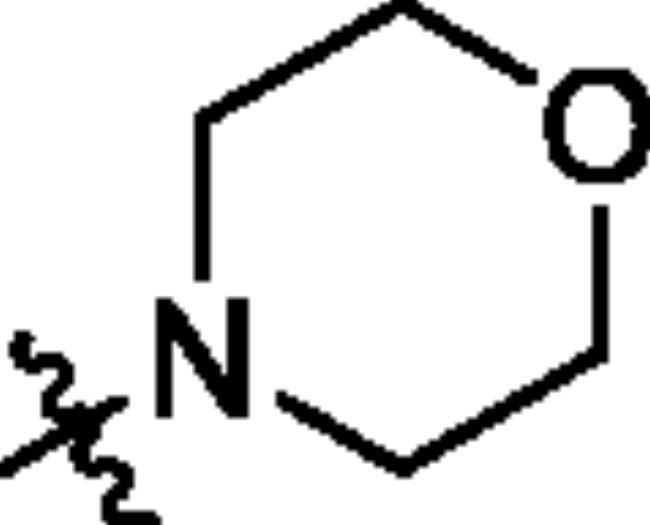	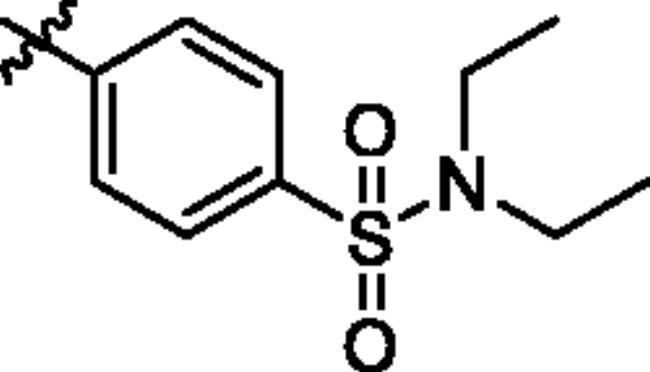	58.5 ± 1.6
**20**	-CH_3_	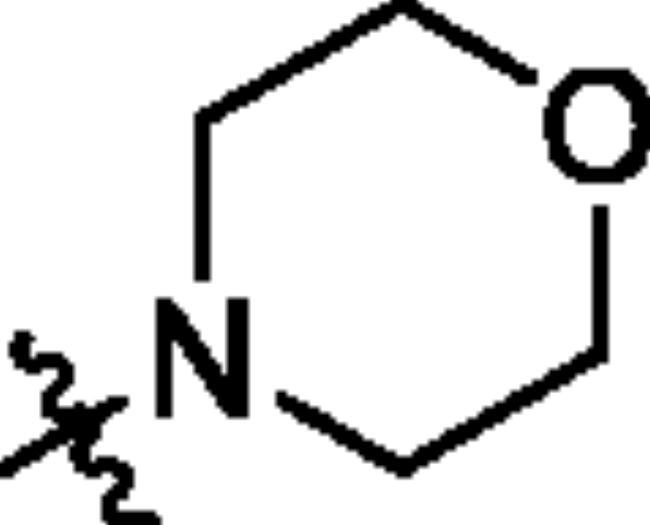	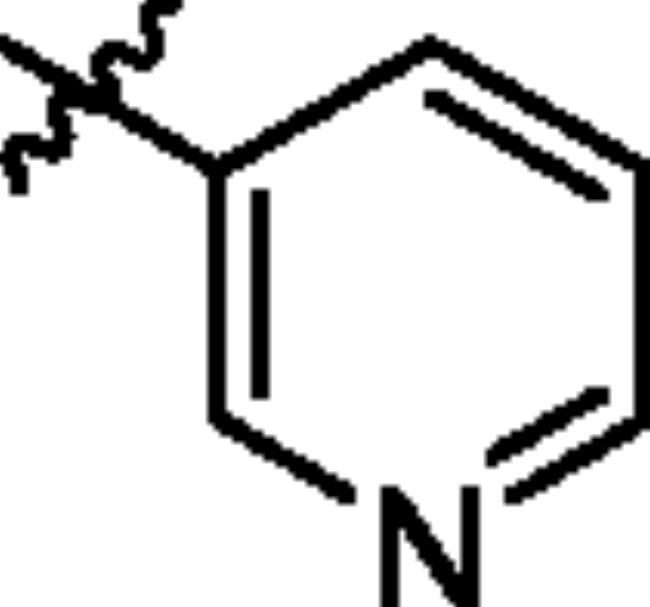	30.2 ± 2.1
**21**	-CH_3_	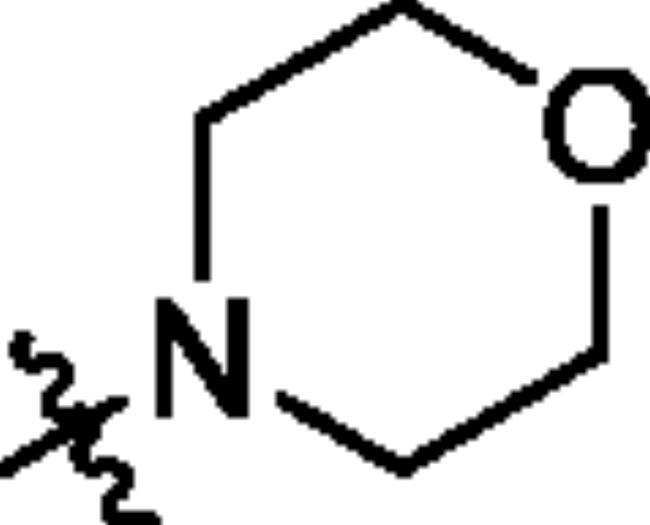	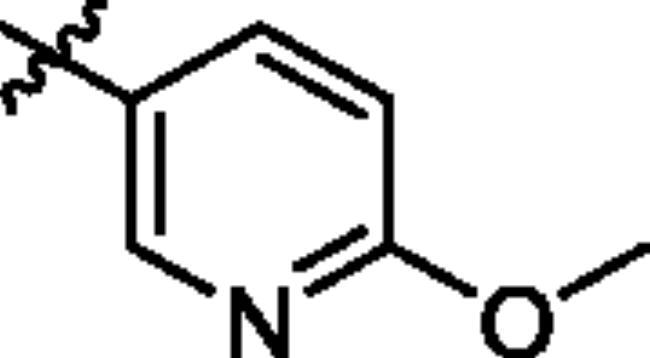	39.9 ± 3.0
**22**	-CH_3_	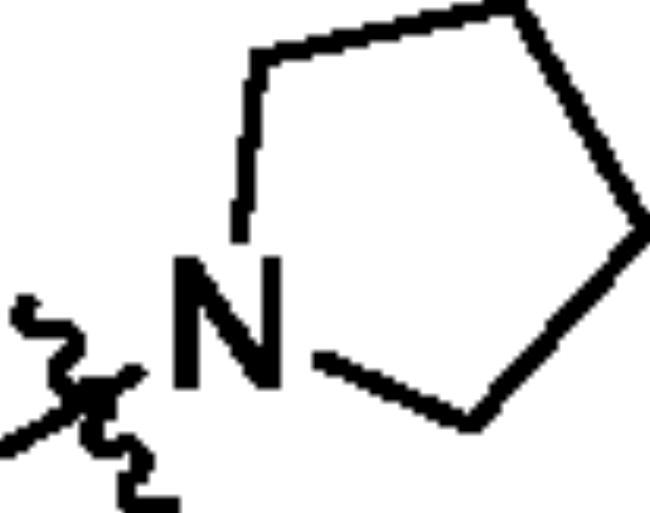	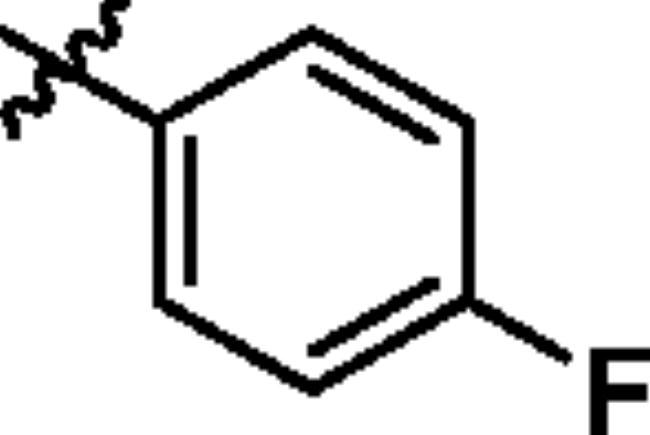	33.3 ± 4.2
**23**	-CH_3_	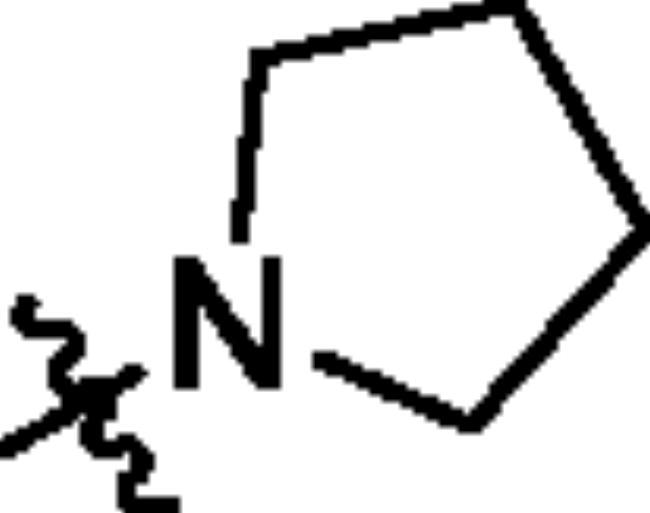	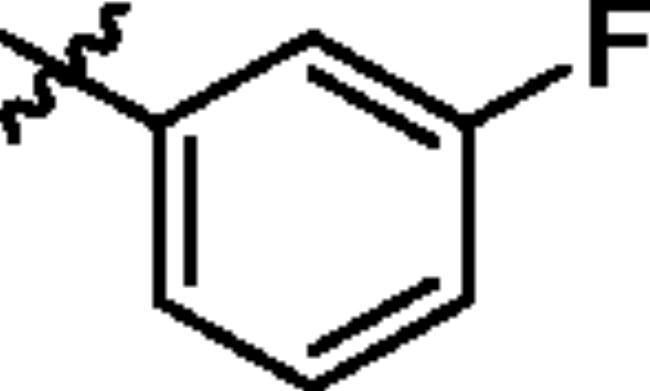	43.9 ± 1.8
**24**	-CH_3_	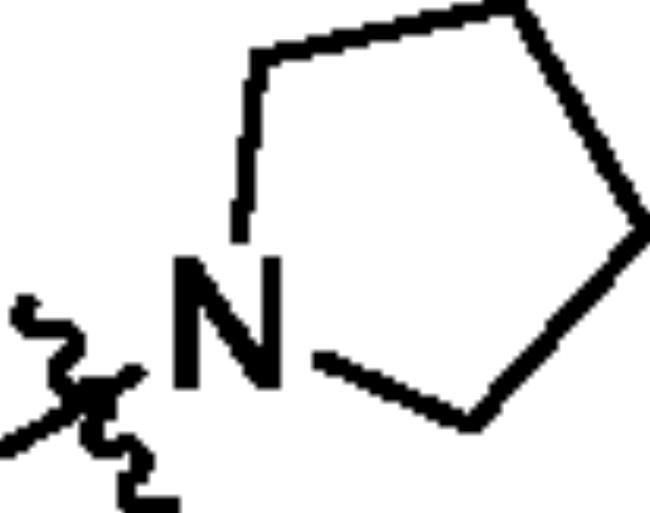	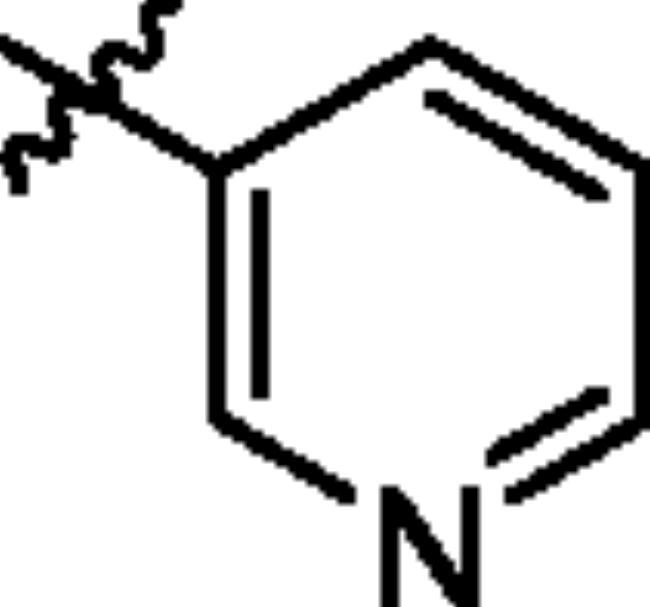	29.6 ± 2.1
**25**	-CH_3_	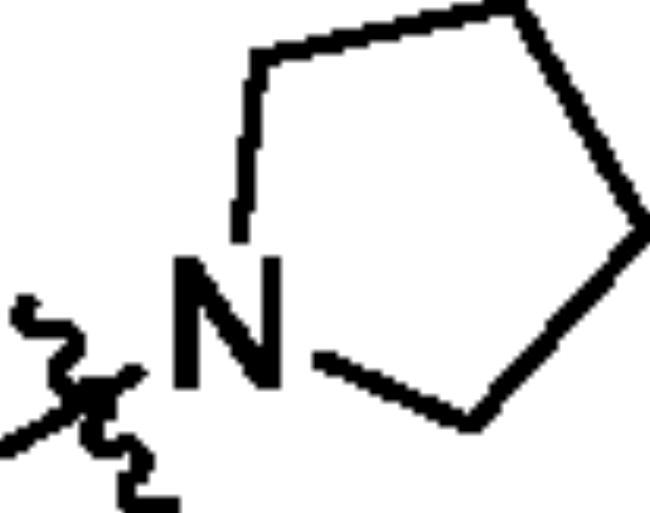	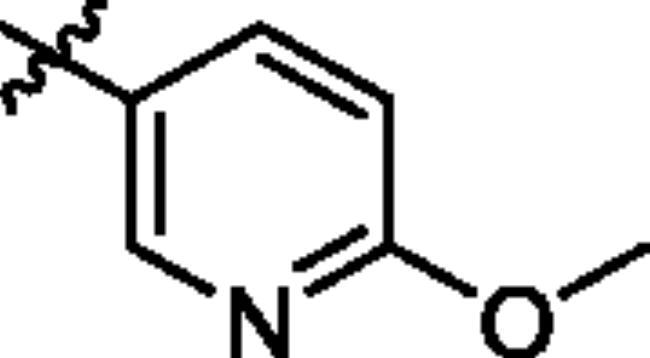	44.3 ± 1.5
**26**	-CH_3_	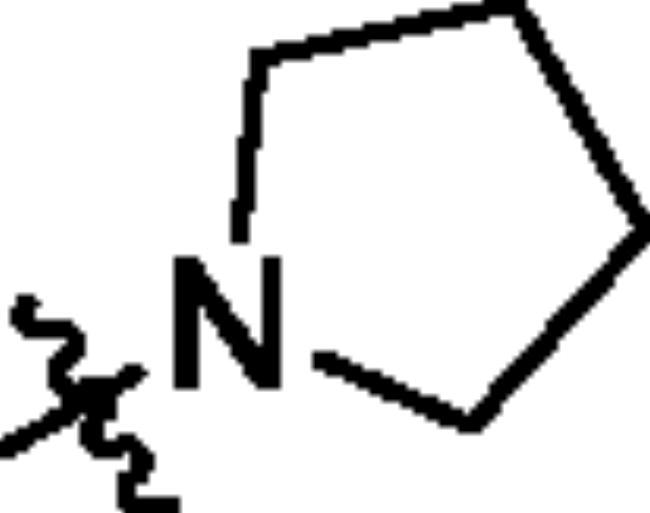	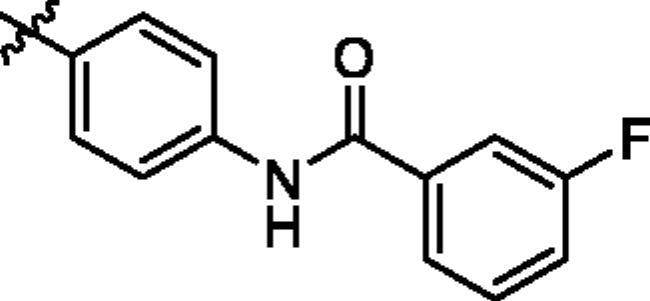	57.9 ± 4.6
**27**	-CH_3_	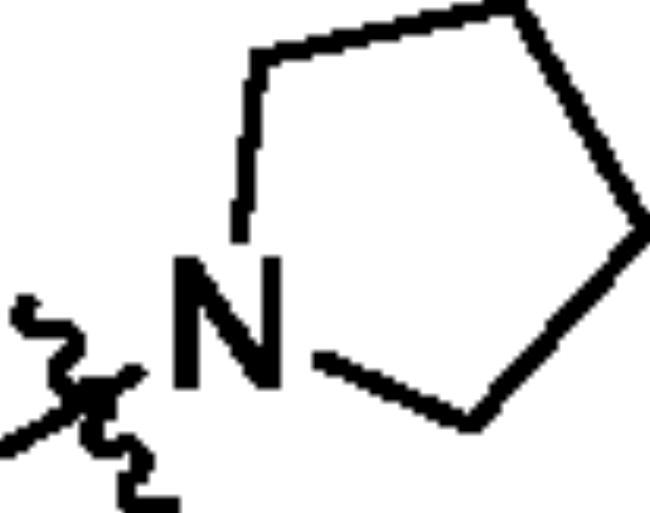	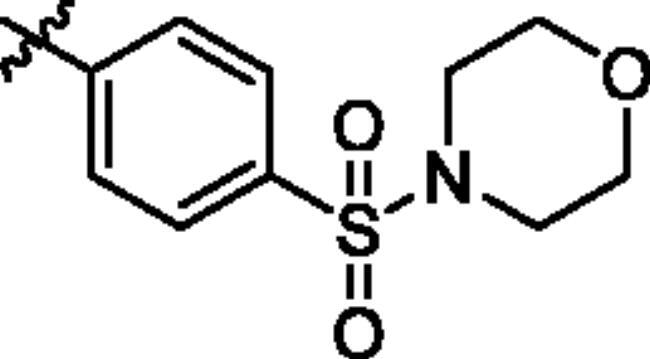	60.9 ± 6.0
**28**	-CH_3_	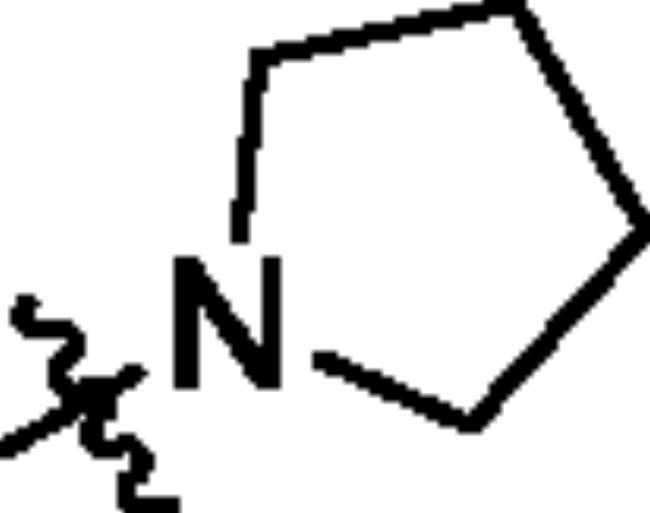	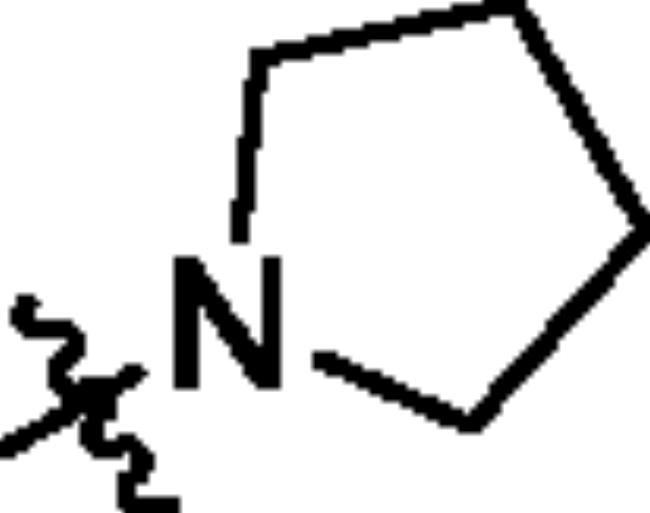	66.1 ± 4.4
**29**	-CH_3_	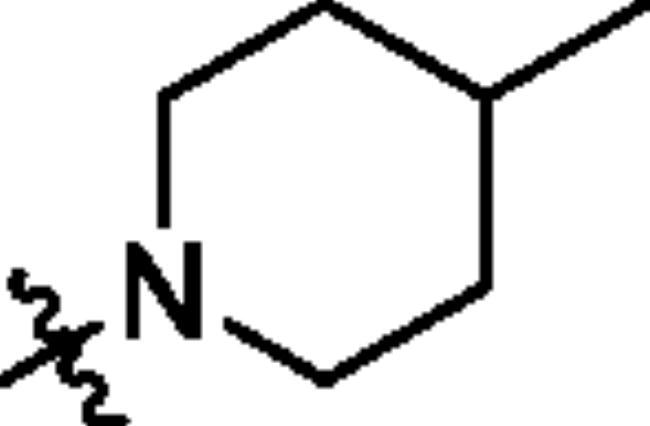	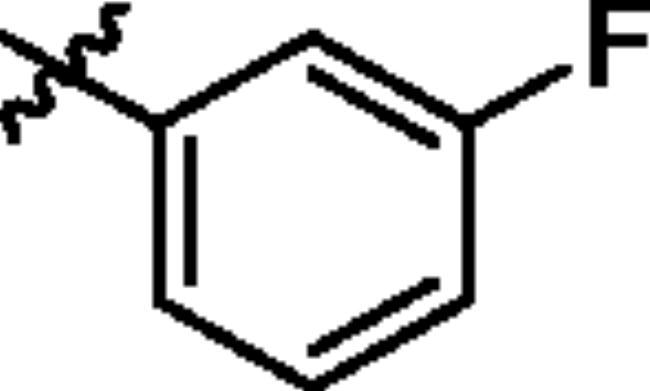	30.4 ± 3.1
**30**	-CH_3_	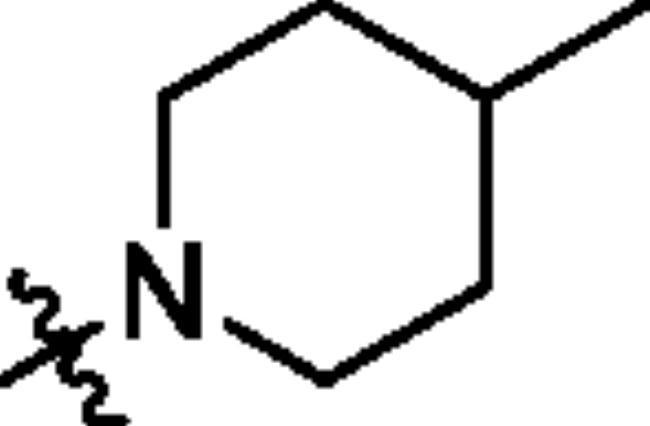	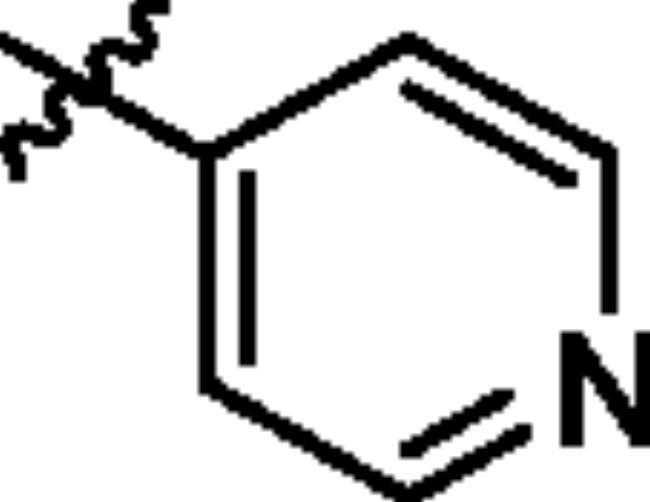	9.3 ± 1.7
**31**	-CH_3_	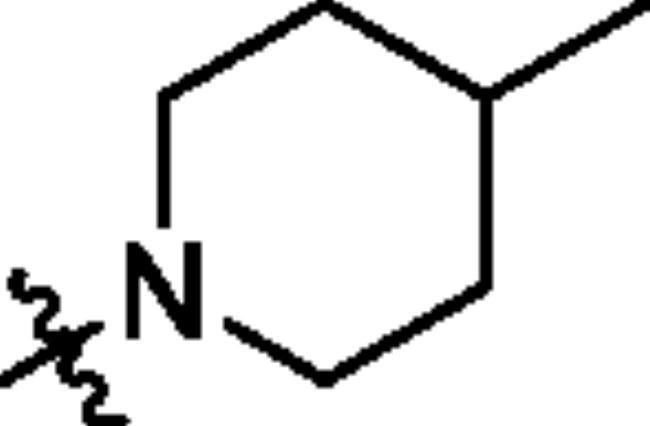	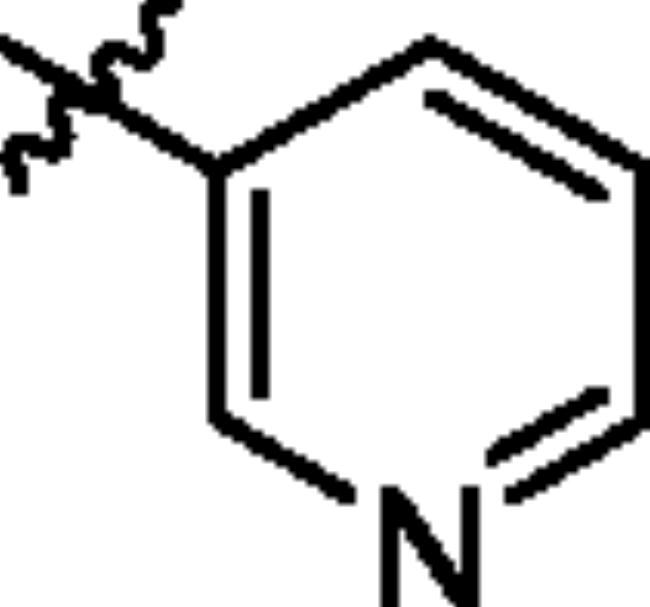	28.5 ± 1.6
**32**	-CH_3_	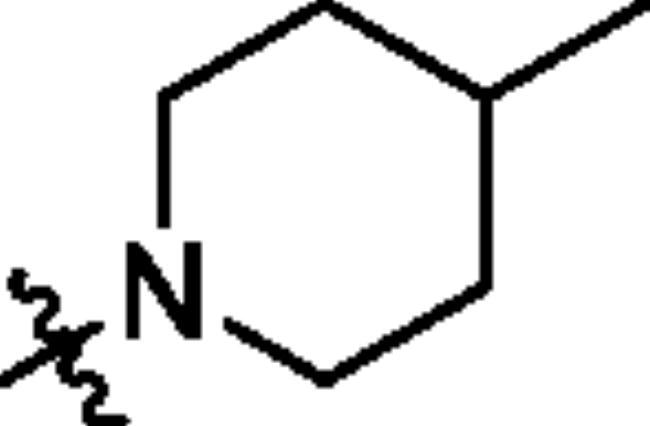	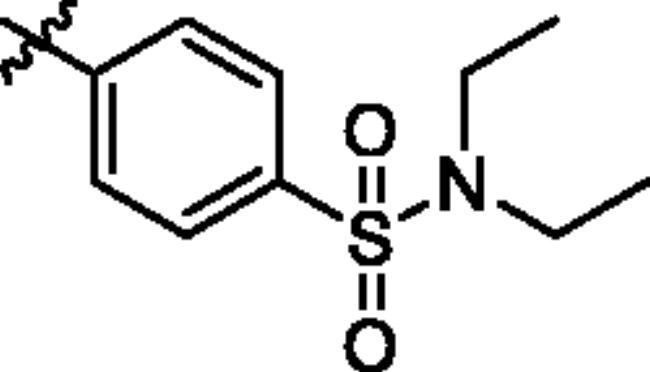	50.6 ± 0.9
**33**	-CH_3_	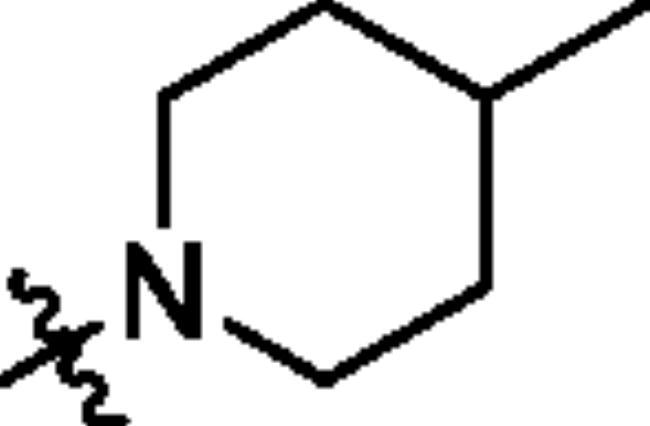	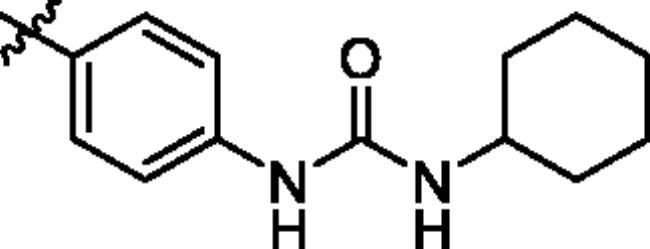	44.5 ± 1.2
**34**	-CH_3_	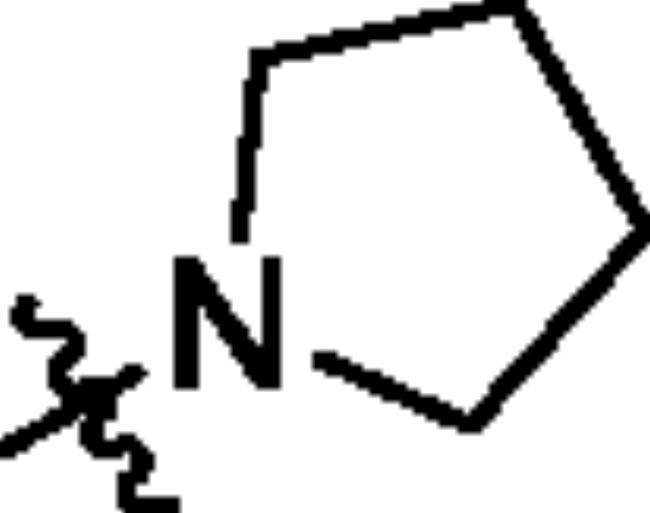	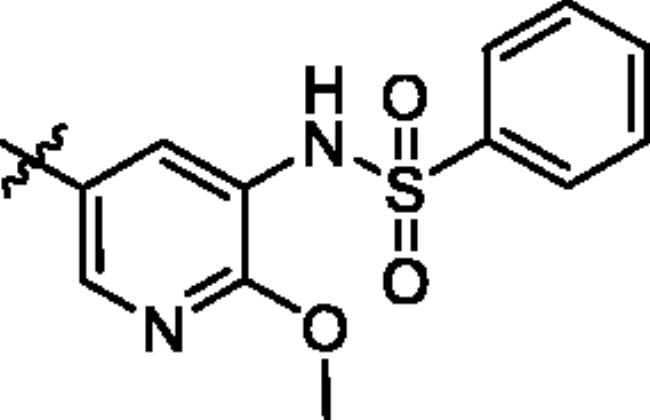	49.8 ± 1.6
**35**	-CH_3_	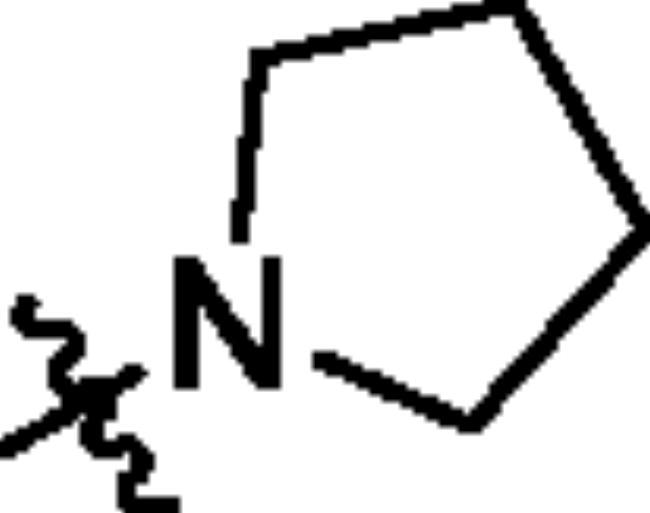	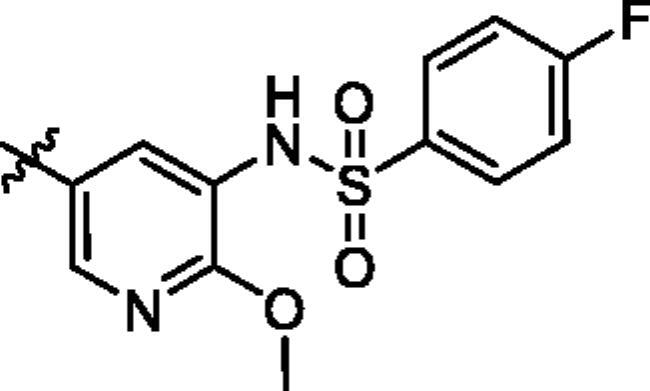	98.6 ± 0.4
**36**	-CH_3_	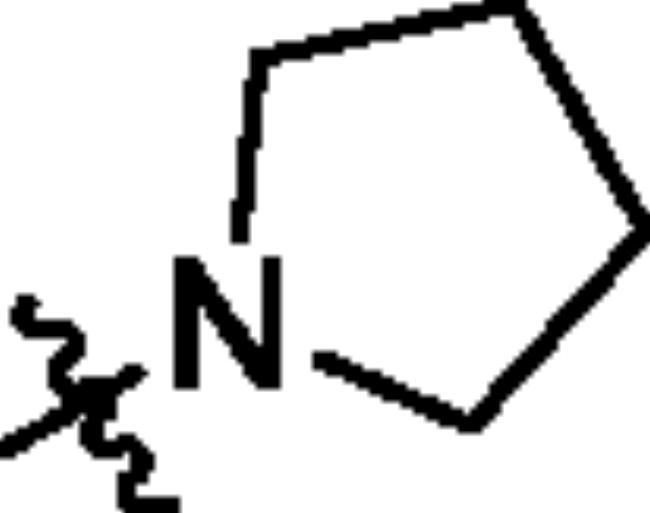	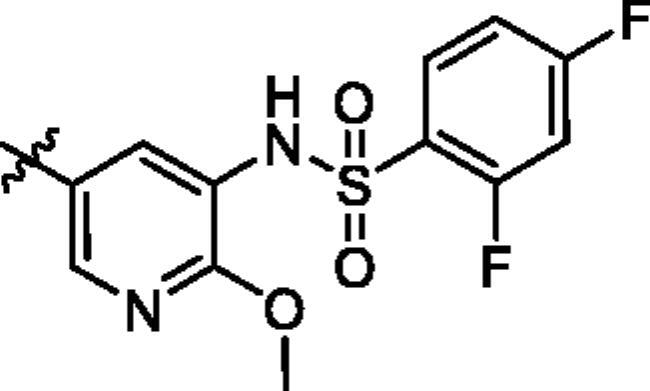	85.1 ± 2.2
**37**	-CH_3_	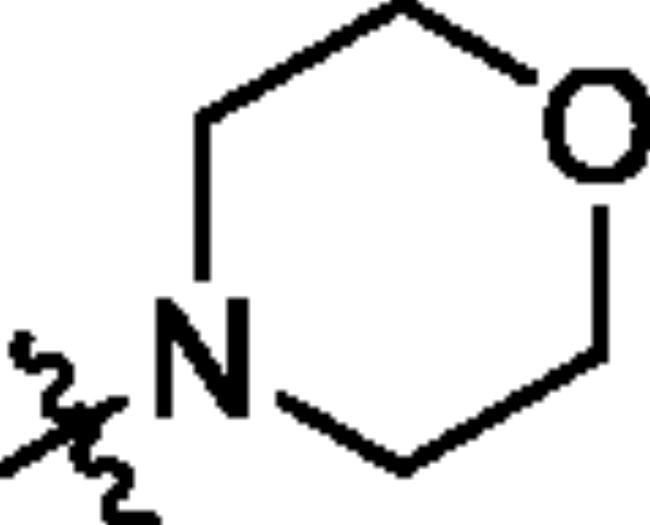	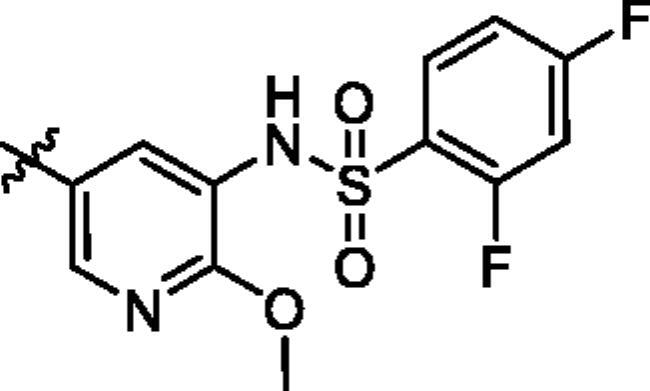	67.5 ± 0.3
**38**	-CH_3_	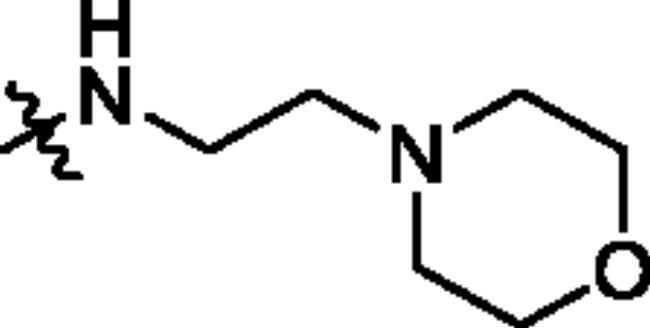	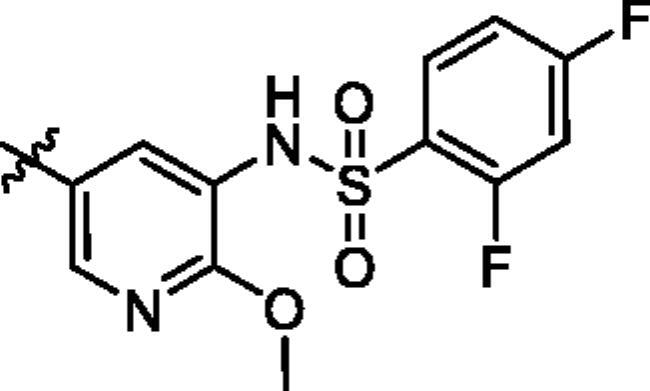	81.6 ± 2.8
**39**	-CH_3_	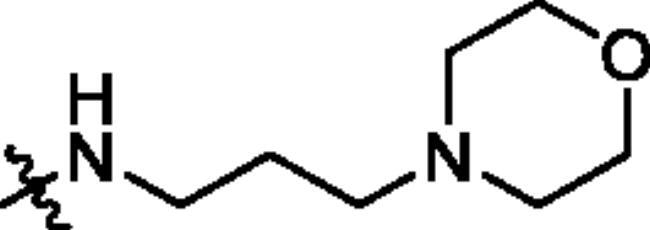	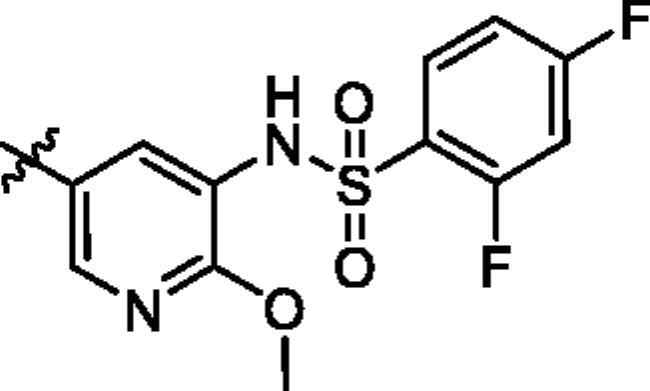	70.2 ± 3.6
**40**	-CH_3_	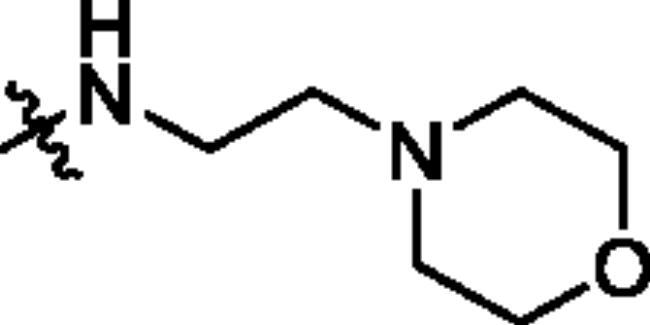	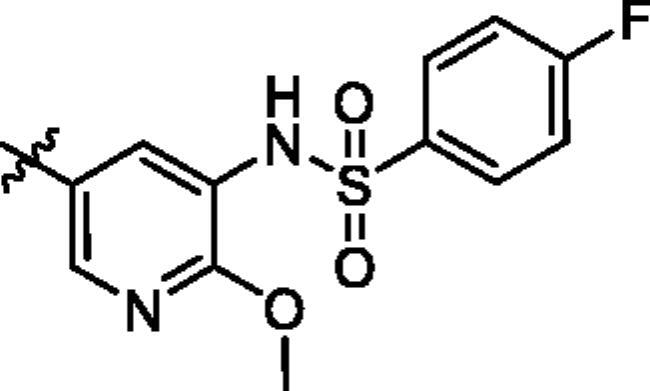	90.1 ± 4.0
**41**	-CH_3_	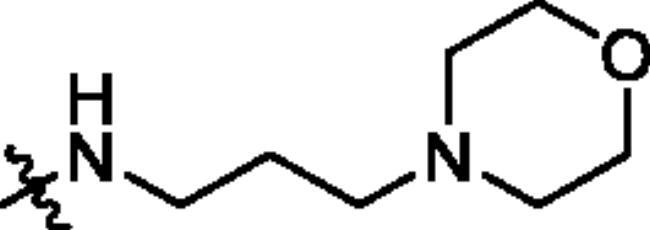	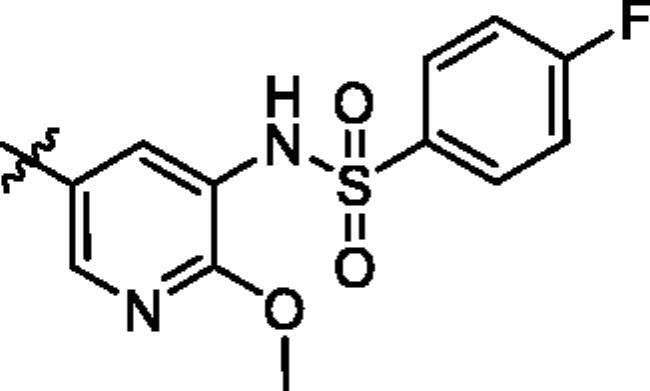	75.7 ± 2.9
**42**	-CH_3_	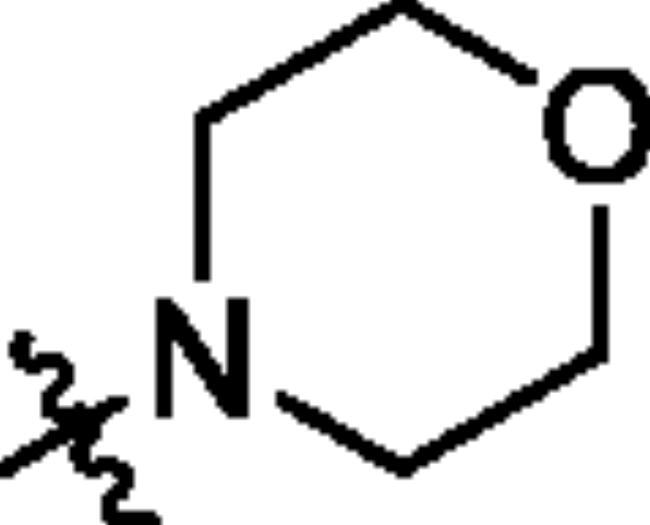	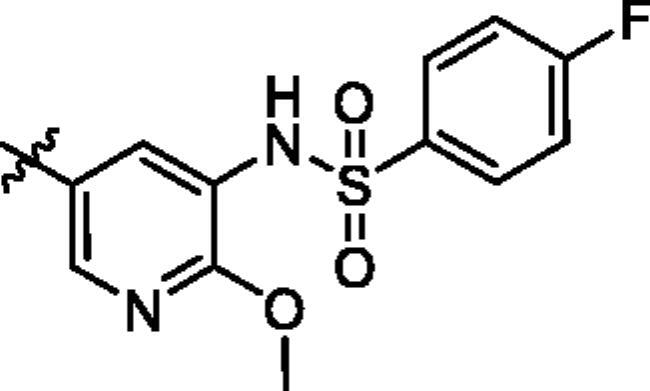	69.2 ± 6.4
**43**	-CH_3_	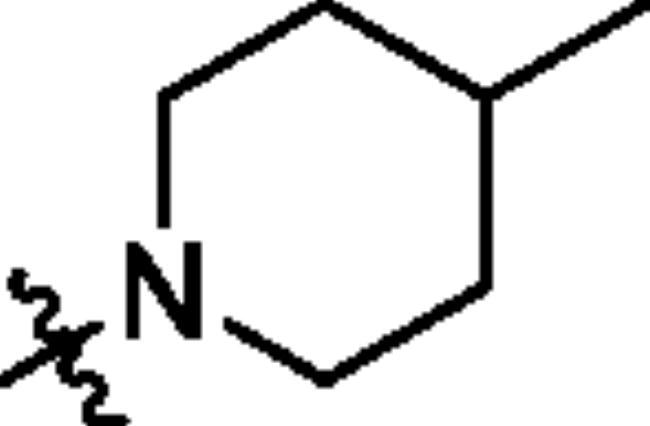	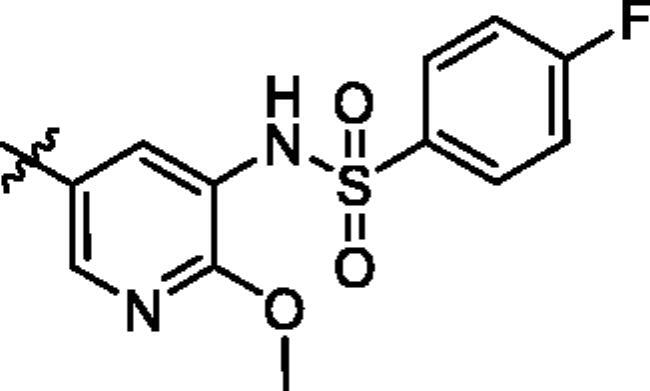	62.8 ± 4.6
**44**	-CH_3_	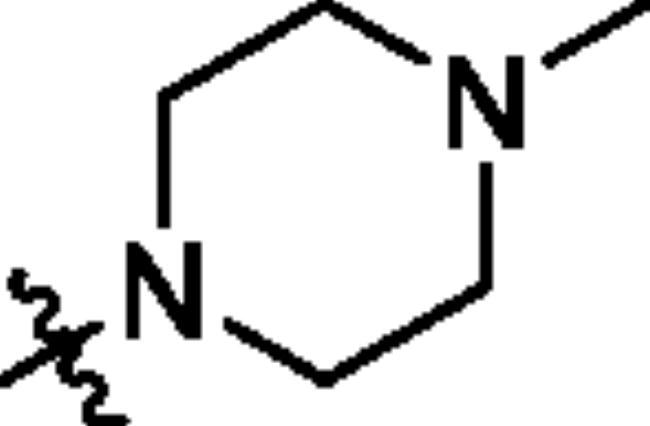	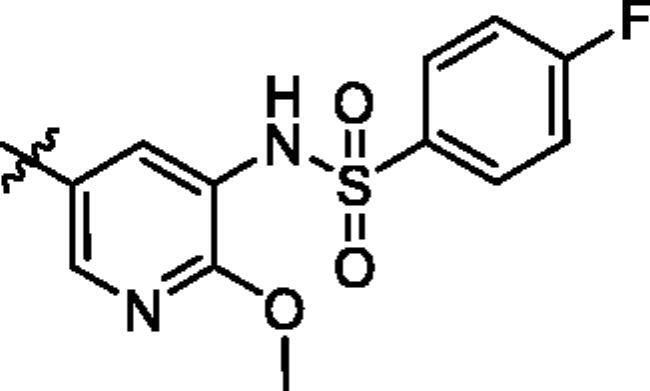	53.4 ± 5.1
**45**	-Cl	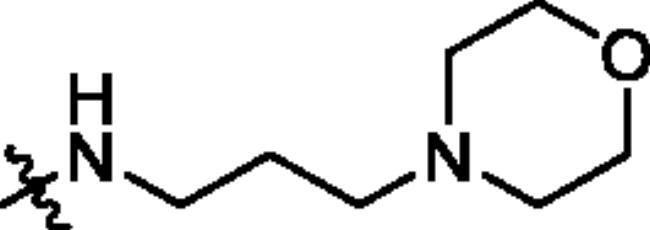	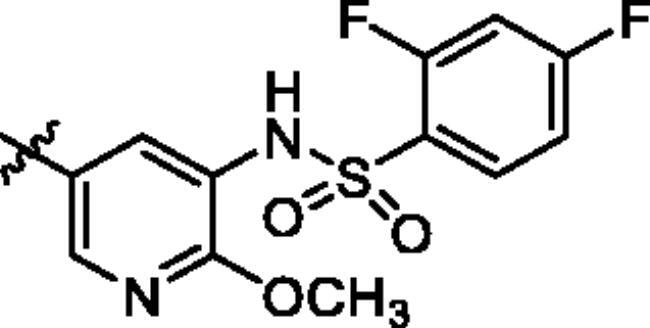	59.8 ± 3.5
**46**	-H	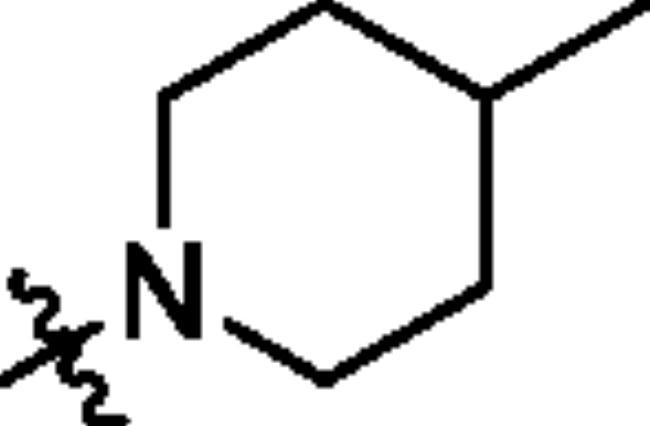	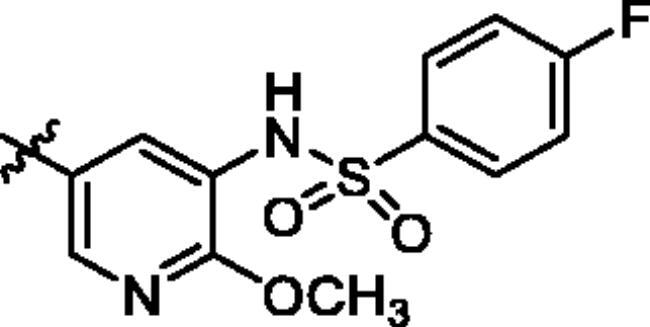	40.2 ± 3.0
**PIK-75^b^**				98.2 ± 1.6

^a^Data presented is the mean ± SD value of three independent determinations.

^b^Used as positive control.

**Figure 3.**
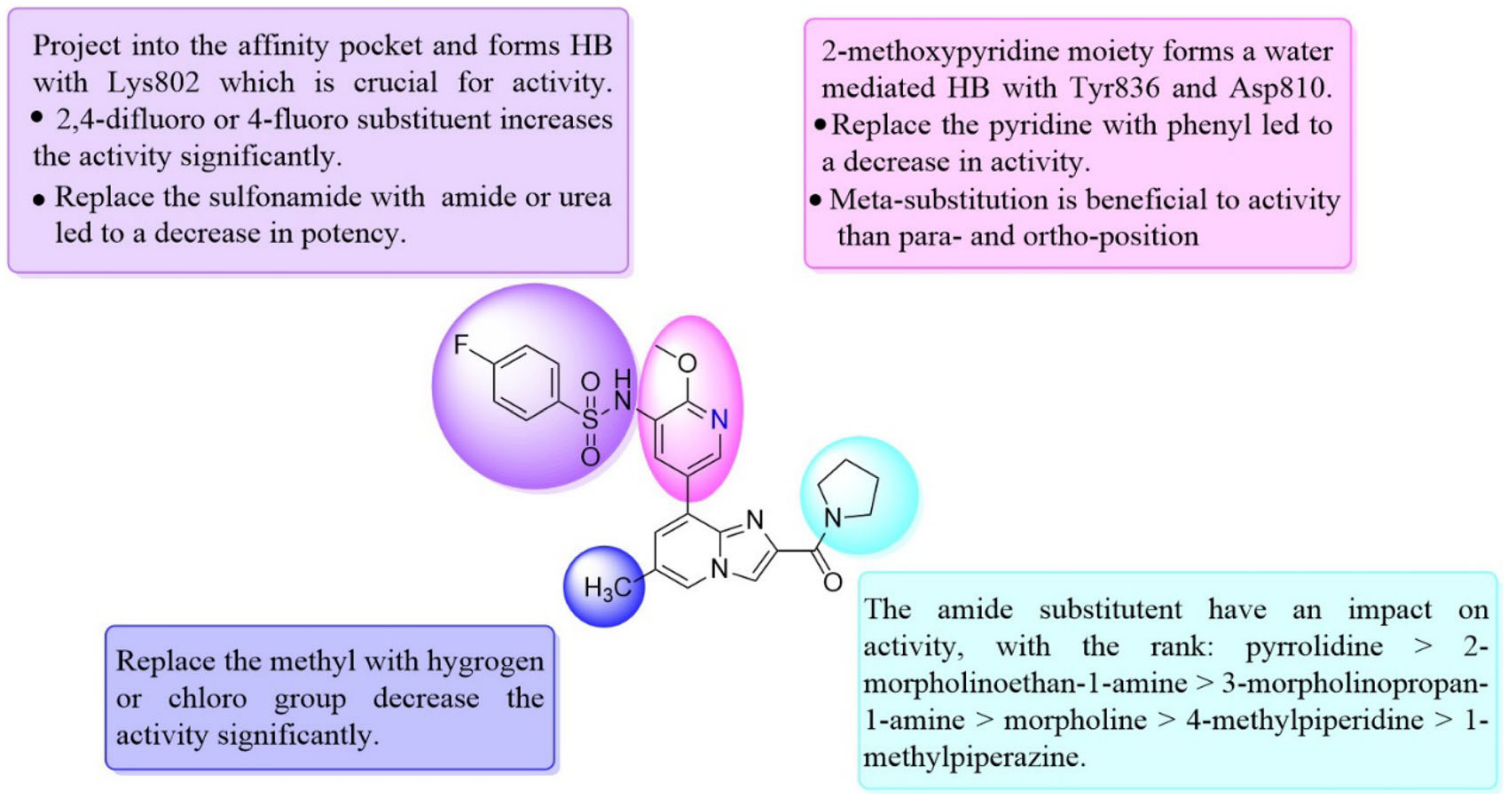
SAR of compound **35** represented in diagram.

Unable to significantly improve the potency on the phenyl group at the 8-position of the Imidazo[1,2-a]pyridine template, we examined the binding mode of PIK-75 to PI3K*α*, the removing sulfonohydrazide group formed an important H-bond with PI3K*α*. To compensate for this loss, we speculated that the pyridinephenylsulfonamide on the 8-position would project into the affinity binding pocket of PI3K*α*, making H-bond with Lys802 which is beneficial for potency. Thus, compounds **34**–**36** were prepared. However, compound **34** offered no advantage over the amide or sulphonamide analogs (**26–28**). Gratifying, a substantial increase in potency was observed when 4-fluoro or 2, 4-difluoro group were further incorporated (**35**, **36** vs **34**). Having established the pyridinesulfonamide on the 8-position in **35** as the optimal substituent, different amines on the 2-position of the Imidazo[1,2-a]pyridine core were also designed and explored (**40–44**), since they may have influence on potency as well as physicochemical properties. The results revealed that all the compounds exhibit moderate to excellent inhibitory rate, ranging from 53.4 to 90.1, with the rank: pyrrolidine > 2-morpholinoethan-1-amine > 3-morpholinopropan-1-amine > morpholine > 4-methylpiperidine > 1-methylpiperazine, which was further demonstrated by compounds **36**–**39**. Finally, the impact of 6-position on activity was also explored, neither the chloro nor the hydrogen substituent showed positive effect on the potency compared to methyl substituent (**45** vs **39**; **46** vs **43**). Overall, The SAR studies depicted above (see also Figure 3) led to the identification **35**, which was the most potent compound that we had obtained.

#### *In vitro* antiproliferative activity

Following the direction, Compounds **35**, **36**, and **40** with inhibitory rate higher than 85% at 10 μM were progressed into full IC_50_ value determination with Alpelisib as positive control. As shown in Table 2, we detected these three compounds exhibit submicromolar activity for PI3K*α*, with IC_50_ of 0.15, 1.12, 0.50 μM, respectively. Given the desirable enzyme activity profile, these compounds were further assayed for their antiproliferative activities against five independent human tumour cell line collections harbouring PIK3CA mutation^23^, including human ovarian cancer cell line SKOV-3, human breast cancer T47D and MCF-7, human lung cancer H1975 and H460. As shown in Table 2, all compounds exhibited antiproliferative effects at micromolar concentrations. In general, the cellular activity of tested derivatives corrected with their effects on kinase activity. Interestingly, these PI3K inhibitors were more sensitive with human breast cancer than non-small cell lung cancer and ovarian cancer. Notably, compound **35** potently inhibited the proliferation of human breast cancer lines T47D and MCF-7 with IC_50_ of 7.9 and 9.4 μM, respectively. Hence, compound **35** was selected for further evaluation.

**Table 2.** Enzymatic and cellular effects of compounds against PI3K***α*** and five Human Cancer Cells.

Compound	IC_50_ in μM^a^					
	PI3K*α*	SKOV-3	T47D	NCI-H1975	NCI-H460	MCF-7
**35**	0.15 ± 0.02	26.6 ± 1.3	7.9 ± 0.8	32.1 ± 2.4	17.7 ± 2.0	9.4 ± 0.6
**36**	1.12 ± 0.15	35.3 ± 4.2	15.9 ± 1.5	>50	30.2 ± 3.1	8.1 ± 0.8
**40**	0.50 ± 0.11	25.5 ± 3.9	12.5 ± 1.8	41.4 ± 3.4	21.8 ± 1.9	7.6 ± 0.5
**Alpelisib^b^**	0.0074 ± 0.0021	9.7 ± 0.7	0.46 ± 0.05	2.7 ± 0.2	8.9 ± 0.7	2.2 ± 0.3

^a^IC_50_ values were determined after exposure of cells to derivatives for 72 h, and data are expressed as the mean ± SD of two independent experiments; ^b^Used as a positive control.

#### Flow cytometry

**Figure 4.**
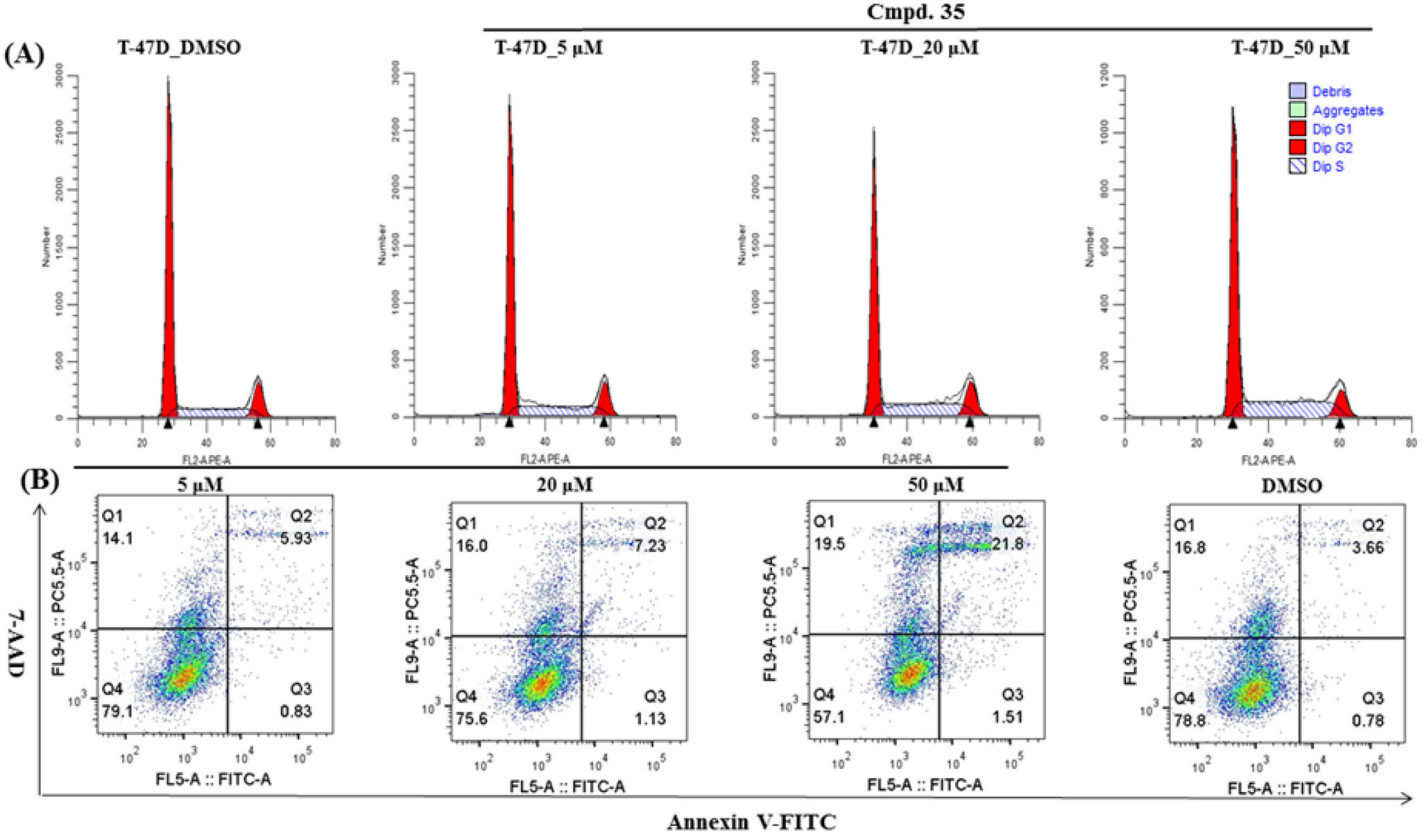
Compound **35** induces S cell cycle arrest and apoptosis in T47D cells. Exponentially growing cells were exposed to DMSO or indicated concentration of compound **35** for 24 h in T47D cells.

Considering compound **35** was most sensitive in T47D cells, we decided to get some mechanistic insight into the mode of action in T47D cell line. As such, the annexin V/7-AAD staining and cell cycle phase distribution have been carried out. In line with its activity, compounds **35** induced a S cell cycle arrest in T47D cells. Furthermore, T47D cells were also treated with different concentrations of **35**, and the percentage of apoptotic cells was measured. Exposure to 50 μM of **35** resulted in an induction of early (1.51%) and late (21.8%) apoptosis of T47D cells (Figure 4).

#### *In vitro* ADME properties of compound 35

To assess its druggability, *In vitro* ADME properties of compound **35** were also determined. As depicted in Table 3, compound **35** showed acceptable stability both in human liver microsomes (HLM) and mouse liver microsomes (RLM), with T_1/2_ of 45.1 and 31.9 min, respectively. To screen its membrane permeability, Parallel artificial membrane permeability assay (PAMPA) was conducted and the result revealed that compound **35** was highly permeable, with permeability rates (P_e_) higher than 10 nm/s. Besides, to avoid any drug-drug interaction, compound **35** was further screened for its cytochrome P450 inhibition activity, and **35** showed little or minimal inhibitions for 5 major cytochrome P450 (CYP1A2, 2D6, 2C9, 2C19, and 3A4) enzymes.

**Table 3.** *In Vitro* ADME properties for compound **35**.

Metabolic stability^a^								
Species		T_1/2_ (min)		CL _int_ (μL/min/mg)			Remaining (T = 60min)	
RLM		31.9		43.4			31.5%	
HLM		45.1		30.7			42.7%	
Permeability assay^b^								
Egg-PAMPA		Mean Pe (nm/s)		Incubation Time (h)			Permeability	
–		15.4		4			High	
CYP450 inhibition^c^								
isozyme	CYP1A2		CYP2D6		CYP2C9	CYP2C19		CYP3A4
% inhibition	9.8		13.5		30.6	25.9		42.6

^a^Testosterone and Diclofenac were used at positive control. ^b^The permeability assay was Performed at 10 μM concentration. High permeability: Pe > 10.0 nm/s. ^c^Performed at 10 μM concentration. α-Naphthoflavone (CYP1A2), Squinidine (CYP2D6), (+)-N-3-benzylnirvanol (CYP2C19), Quinidine (CYP2D6), and ketoconazole (CYP3A4) were used as the positive controls.

### Molecular modelling and dynamic simulation

**Figure 5.**
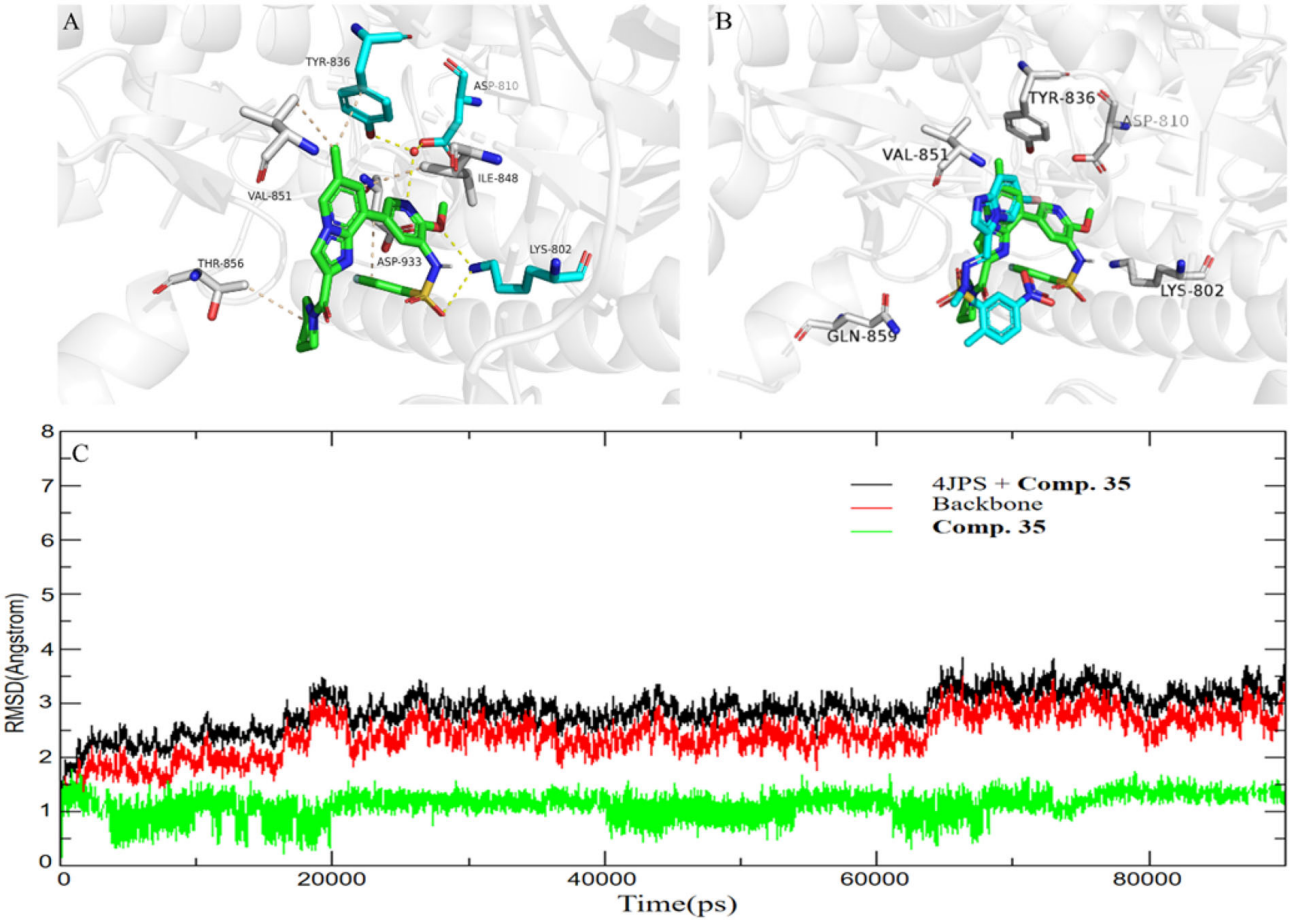
(A) Proposed binding mode of compound **35** to PI3K*α* (4JPS). Hydrogen bonds are indicated by yellow dashed lines, and hydrophobic interactions are indicated in wheat lines. Images are generated using PyMol. (B) The overlay of compound **35** with PIK-75 in PI3K*α* (4JPS). (C) Dynamics of compound **35** bound to FLT3 PI3Kα (4JPS) during 90 ns simulation time.

To better understand the activity of **35** against PI3K*α*, the binding mode between the **35** and PI3K*α* was then studied, and the structure of PI3K*α* (PDB ID code: 4JPS)^18^ was selected as the docking model. Compound **35** adopted a similar binding mode as previously reported PI3K*α* inhibitors^24^^,^^25^. As shown in Figure 5(A), the methoxy in 2-position of pyridine and the oxygen of sulphonamide in compound **35** formed two H-bond donor–acceptor interactions with Lys802, and the pyridine nitrogen atom of **35** is part of a hydrogen bond network involving the conserved water molecule with the side chains of residues Tyr836 and Asp810. In addition, compound **35** also made hydrophobic interactions within Ile848, Val851, Thr856, and Asp933, respectively. The overlay of **35** with PIK-75 revealed the pyrrolidine amide moiety formed weaker interactions with PI3K*α* than that of PIK-75 (Figure 5(B)). However, the binding affinity of compound **35** with PI3K*α* was partly compensated by forming strong hydrogen bonds with the backbone of Tyr836 and Asp810 than that of a bromine atom in the Imidazopyridine ring of PIK-75. To further access the binding stability of compound **35/**PI3K*α* adducts, a 90 ns long molecular dynamics simulation was carried out. The RMSD values of protein backbone atoms relative to the initial structure were calculated to examine the protein stability over the course of the simulation period. As shown in Figure 5(C), the RMSD did not fluctuate significantly, and the value of RMSD converged to 3.0 Å at 90 ns, which revealed that compound **35** could stably bind to PI3K*α*. In general, the docking result confirm the rationality of our design strategy.

## Conclusion

In summary, 32 novel compounds bearing Imidazo[1,2-a]pyridine scaffold were designed and synthesised. The design strategy of PI3K*α* inhibitor involved modification of the 2,6,8-position of Imidazo[1,2-a]pyridine ring. Systematic SAR studies led us identified **35** as the most promising compound, with IC_50_ of 150 nM against PI3K*α* in enzymatic assay. By screening a panel of cancer cell lines that harboured PIK3CA mutation, compound **35** was found to be more potent against breast cancer than others. Further *in vitro* ADME evaluation demonstrated **35** was metabolic stable in RLM and HLM, highly permeable in PAMPA, and not a CYP450 inhibitor. More work regarding the optimisation of **35** to achieve a more potent PI3K*α* inhibitor will be disclosed in due course.

## Experimental

### Chemistry

Unless otherwise specified, all the reagents and solvents were obtained from commercial sources and used without further purification. Reactions’ time and purity of the products were monitored by TLC on FLUKA silica gel aluminium cards (0.2 mm thickness) with fluorescent indicator 254 nm. All melting points were obtained on a Büchi Melting Point B-540 apparatus (Büchi Labortechnik, Flawil, Switzerland) and were uncorrected. Flash chromatography was performed using silica gel (200–300 mesh) from Qingdao ocean Chemicals (Qingdao, Shandong, China). The positive control PIK-75 and Alpelisib were purchased from TargetMol (Shanghai, China). Mass spectra (MS) were taken in ESI mode on Waters e2695. ^1^H NMR and ^13^C NMR spectra were recorded on Bruker ARX-400 or ARX-600, 400 or 600 MHz spectrometers (Bruker Bioscience, Billerica, MA, USA) with TMS as an internal standard.

### General procedures

#### General procedure for preparation of intermediate 8a-c

To a solution of 5-bromo-2-methoxypyridin-3-amine (2.0 g, 10 mmol) in pyridine (30 mL) was added substituted benzene-1-sulfonyl chloride (15 mmol), the mixture was stirred at room temperature overnight. Upon completion, the mixture was then concentrated and redissolved in 30 mL CH_2_Cl_2_, washed with aqueous citric acid and then brine. After drying over Na_2_SO_4_, the solvent was evaporated and used without further purification.

##### N-(5-bromo-2-methoxypyridin-3-yl)benzenesulfonamide (8a)

White solid; Yield: 67.8%; HRMS(ESI) calculated for C_12_H_12_BrN_2_O_3_S [M + H]^+^ m/z 342.9674, found: 342.9679.

##### N-(5-bromo-2-methoxypyridin-3-yl)-4-fluorobenzenesulfonamide (8b)

White solid; Yield: 63.5%; HRMS(ESI) calculated for C_12_H_11_BrFN_2_O_3_S [M + H]^+^ m/z 360.9580, found: 360.9589.

##### N-(5-bromo-2-methoxypyridin-3-yl)-2,4-difluorobenzenesulfonamide (8c)

Yellow solid; Yield: 75.4%; HRMS(ESI) calculated for C_12_H_10_BrF_2_N_2_O_3_S [M + H]^+^ m/z 378.9485, found: 378.9480.

#### General procedure for preparation of intermediate 9a-c

A solution of **8a-c** (3.2 mmol), bis(pinacolato)diboron (1.6 g, 6.4 mmol), PdCl_2_(dppf)-DCM (258 mg, 0.32 mmol), potassium acetate (932 mg, 9.4 mmol) in 1,4-dioxane (20 mL) was heated at 100 °C under N_2_ for 8 h. Upon cooling, the mixture was diluted with CH_2_Cl_2_ (100 mL) and then filtered, the organic layers were washed with brine, dried over Na_2_SO_4_, and concentrated. The residue was then subjected to flash chromatography to give pure ***9a-c***.

##### N-(2-methoxy-5–(4,4,5,5-tetramethyl-1,3,2-dioxaborolan-2-yl)pyridin-3-yl)ben zenesulfonamide (9a)

White solid; Yield: 81.2%; HRMS(ESI) calculated for C_18_H_23_BN_2_O_5_S [M + H]^+^ m/z 391.1499, found: 391.1508.

##### 4-Fluoro-N-(2-methoxy-5–(4,4,5,5-tetramethyl-1,3,2-dioxaborolan-2-yl)pyridine-3-yl)benzenesulfonamide (9 b)

White solid; Yield: 78.5%; HRMS(ESI) calculated for C_18_H_22_BFN_2_O_5_S [M + H]^+^ m/z 409.1405, found: 409.1411.

##### 2,4-Difluoro-N-(2-methoxy-5–(4,4,5,5-tetramethyl-1,3,2-dioxaborolan-2-yl)pyridin-3-yl)benzenesulfonamide (9c)

White solid; Yield: 76.7%; HRMS(ESI) calculated for C_18_H_21_BF_2_N_2_O_5_S [M + H]^+^ m/z 427.1311, found: 427.1323.

#### General procedure for preparation of intermediate 11a-b

NBS (32.9 g, 184.9 mmol) was slowly added into the solution of 2-aminopyridine derivatives (184.9 mmol) in N, N-dimethylformamide (200 mL) under ice bath, the reaction was then stirred at 0 °C for 4 h. The mixture was poured into ice water (400 mL) when the reaction was completed by TLC detection, the aqueous phase was then extracted with methyl tert-butyl ether (80 mL × 3), washed with brine, and concentrated under reduced pressure to give **11a-b**.

##### 3-Bromo-5-methyl-1,2-dihydropyridin-2-amine (11a)

Brown solid; Yield: 89.5%; HRMS(ESI) calculated for C_6_H_10_BrN_2_ [M + H]^+^ m/z 189.0027, found: 189.0035.

##### 3-Bromo-5-chloro-1,2-dihydropyridin-2-amine (11b)

White solid; Yield: 86.9%; HRMS(ESI) calculated for C_5_H_7_BrClN_2_ [M + H]^+^ m/z 208.9481, found: 208.9496.

#### General procedure for preparation of intermediate 12a-c

Commercially available 2-aminopyridine or **11a-b** (181.8 mmol), ethyl bromopyruvate (44.3 g, 227.2 mmol) were successively added to ethanol (300 mL), the mixture was heated to 80 °C for 4 h. Upon completion, the reaction solution was cooled to room temperature, concentrated under reduced pressure to obtain a brownish red solid, which was then triturated with acetone to give **12a-c**.

##### Ethyl 8-bromo-6-methylImidazo[1,2-a]pyridine-2-carboxylate (12a)

Light yellow solid; Yield: 83.3%; HRMS(ESI) calculated for C_11_H_12_BrO_2_N_2_ [M + H]^+^ m/z 283.0082, found: 283.0091.

##### Ethyl 8-bromo-6-chloroImidazo[1,2-a]pyridine-2-carboxylate (12b)

White solid, Yield: 76.6%; HRMS(ESI) calculated for C_10_H_9_ClBrO_2_N_2_ [M + H]^+^ m/z 302.9536, found: 302.9548.

##### Ethyl 8-bromoImidazo[1,2-a]pyridine-2-carboxylate (12c)

Light yellow solid; Yield: 71.1%; HRMS(ESI) calculated for C_10_H_10_BrO_2_N_2_ [M + H]^+^ 268.9926, found: 268.9937.

#### General procedure for preparation of intermediate 13a-c

To a solution of corresponding carboxylate **12a-c** (37.2 mmol) in ethanol (50 mL) was added NaOH solution (15 mL, 1 N), the reaction was heated to 80 °C for 3 h. After cooling, the reaction solution was concentrated under reduced pressure to remove the solvent and the pH was adjusted to 5 with diluted hydrochloric acid (15 mL, 1 N), a large amount of solid was precipitated. The precipitant was filtered, washed with water, and dried under vacuum to afford the products **13a-c**.

##### 8-Bromo-6-methylImidazo[1,2-a]pyridine-2-carboxylic acid (13a)

Light yellow solid; Yield: 74.4%; HRMS(ESI) calculated for C_9_H_6_ClBrO_2_N_2_ [M + H]^+^ m/z 252.9613, found: 252.9625.

##### 8-Bromo-6-chloroImidazo[1,2-a]pyridine-2-carboxylic acid (13b)

Light yellow solid; Yield: 85.0%; HRMS(ESI) calculated for C_8_H_3_ClBrO_2_N_2_ [M + H]^+^ m/z 272.9066, found: 272.9079.

##### 8-Bromoimidazo[1,2-a]pyridine-2-carboxylic acid (13c)

Light yellow solid; Yield: 77.3%; HRMS(ESI) calculated for C_8_H_4_ClBrO_2_N_2_ [M + H]^+^ m/z 238.9456, found: 238.9473.

#### General procedure for preparation of intermediate 14a-h

To a solution of corresponding carboxyl acid **13a-c** (1.2 mmol) in DMF was subsequently added morpholine (0.31 g, 3.5 mmol), triethylamine (0.17 g, 1.8 mmol) and HBTU (0.67 g, 1.8 mmol). The reaction was stirred at room temperature overnight. Upon completion, the mixture was then poured into ice-water, extracted with EtOAc (15 mL × 3). The combined extracts were then washed with brine, dried over Na_2_SO_4_, and concentrated. The crude products were then subjected to flash chromatography to give pure products **14a-h**.

##### (8-Bromo-6-methylImidazo[1,2-a]pyridin-2-yl)(morpholino)methanone (14a)

White solid; Yield: 45.3%; HRMS(ESI) calculated for C_13_H_15_BrO_2_N_3_ [M + H]^+^ m/z 324.0348, found: 324.0376.

##### (8-Bromo-6-methylImidazo[1,2-a]pyridin-2-yl)(4-methylpiperazin-1-yl)methanone (14 b)

Gray white solid; Yield: 50.3%; HRMS(ESI) calculated for C_14_H_18_BrON_4_ [M + H]^+^ m/z 337.0664, found: 337.0711.

##### (8-Bromo-6-methylImidazo[1,2-a]pyridin-2-yl)(pyrrolidin-1-yl)methanone (14c)

Light yellow solid; Yield: 48.9%; HRMS(ESI) calculated for C_13_H_15_BrON_3_ [M + H]^+^ m/z 308.0398, found: 308.0408.

##### (8-Bromo-6-methylImidazo[1,2-a]pyridin-2-yl)(4-methylpiperidin-1-yl)methanone (14d)

White solid; Yield: 63.1%; HRMS(ESI) calculated for C_15_H_19_BrON_3_ [M + H]^+^ m/z 336.0712, found: 336.0765.

##### 8-Bromo-6-methyl-N-(2-morpholinoethyl)imidazo[1,2-a]pyridine-2-carboxamide (14e)

White solid; Yield: 69.4%; HRMS(ESI) calculated for C_15_H_20_BrO_2_N_4_ [M + H]^+^ m/z 367.0770, found: 367.0780.

##### 8-Bromo-6-methyl-N-(3-morpholinopropyl)imidazo[1,2-a]pyridine-2-carboxamide (14f)

Light yellow solid; Yield: 57.6%; HRMS(ESI) calculated for C_16_H_22_BrO_2_N_4_ [M + H]^+^ m/z 381.0926, found: 381.0911.

##### 8-Bromo-6-chloro-N-(3-morpholinopropyl)imidazo[1,2-a]pyridine-2-carboxamide (14 g)

White solid; Yield: 80.3%; HRMS(ESI) calculated for C_15_H_19_BrClO_2_N_4_ [M + H]^+^ m/z 401.0380, found: 401.0392.

##### (8-Bromoimidazo[1,2-a]pyridin-2-yl)(4-methylpiperidin-1-yl)methanone (14 h)

White solid; Yield: 62.3%; HRMS(ESI) calculated for C_14_H_17_BrF_2_ON_3_ [M + H]^+^ m/z 322.0555, found: 322.0578.

#### General procedure for preparation of target compounds 15–46

Method A: a mixture of corresponding amide (3.1 mmol), xylene: H_2_O = 5:1 (30 mL), arylboric acids (4.8 mmol), potassium carbonate (0.85 g, 6.2 mmol) and Pd(PPh_3_)_4_ (0.25 g, 0.31 mmol) was heated to 80 °C for 8 h under the protection of N_2_. Upon completion, the solvent was removed under reduced pressure, and then poured into water to precipitate solids. The precipitant was filtered, washed with tert-Butyl methyl ether, dried under vacuum to afford the target compounds.

Method B: a mixture of corresponding amide (0.93 mmol), corresponding boric acid ester (1.39 mmol), potassium carbonate (0.26 g, 1.86 mmol), 1,4-dioxane: H_2_O = 3:1 and Pd(PPh_3_)_4_ (0.076 g, 0.093 mmol) was stirred at 90 °C for 12 h under nitrogen atmosphere. After cooling to room temperature, water (30 mL) was added followed by extraction with EtOAc (10 mL × 3). The combined organic layers were dried over MgSO_4_ and concentrated under reduced pressure. The crude products were purified using silica gel column chromatography to give pure compounds.

##### (8–(4-Fluorophenyl)-6-methylImidazo[1,2-a]pyridine-2-yl)(morpholino)methanone (15)

White solid; Yield: 75.0%; *m.p.*: 1 8 0 ∼ 182 °C; ^1^H NMR (400 MHz, CDCl_3_) *δ* 8.15 (s, 1H), 8.12 − 7.98 (m, 2H), 7.96 (s, 1H), 7.23 (s, 1H), 7.22 − 7.18 (m, 2H), 4.52 − 4.42 (m, 2H), 3.89 − 3.79 (m, 6H), 2.43 (s, 3H); ^13^C NMR (100 MHz, DMSO-*d_6_*) *δ* 163.83, 162.77, 161.39, 141.25, 140.01, 132.34, 131.09, 127.41, 124.32, 123.11, 117.44, 115.74, 66.92, 40.99, 18.1. HRMS (ESI) calculated for C_19_H_19_FN_3_O_2_ [M + H]^+^ m/z 340.1461, found: 340.1447.

##### (8–(3-Fluorophenyl)-6-methylImidazo[1,2-a]pyridin-2-yl)(morpholino)methanone (16)

White solid; Yield: 79.6%; *m.p.*: 1 7 7 ∼ 180 °C; ^1^H NMR (400 MHz, DMSO-*d_6_*) *δ* 8.45 (s, 1H), 8.38 (s, 1H), 8.11 (d, *J* = 11.3 Hz, 1H), 8.02 (d, *J* = 7.6 Hz, 1H), 7.62 (s, 1H), 7.60 − 7.54 (m, 1H), 7.30 (t, *J* = 7.6 Hz, 1H), 4.27 − 4.21 (m, 2H), 3.73 − 3.65 (m, 6H), 2.39 (s, 3H). HRMS (ESI) calculated for C_19_H_19_FN_3_O_2_ [M + H]^+^ m/z 340.1461, found: 340.1530.

##### (8–(2-Fluorophenyl)-6-methylImidazo[1,2-a]pyridin-2-yl)(morpholino)methanone (17)

White solid; Yield: 76.4%; *m.p.*: 1 8 6 ∼ 189 °C; ^1^H NMR (400 MHz, CDCl_3_) *δ* 8.14 (s, 1H), 8.00 (s, 1H), 7.85 (t, *J* = 7.4 Hz, 1H), 7.45 (dd, *J* = 13.6, 7.3 Hz, 1H), 7.29 − 7.21 (m, 3H), 4.44 − 4.36 (m, 2H), 3.85 − 3.73 (m, 6H), 2.43 (s, 3H). HRMS (ESI) calculated for C_19_H_19_FN_3_O_2_ [M + H]^+^ m/z 340.1461, found: 340.1451.

##### 3-Fluoro-N-(4–(6-methyl-2-(morpholine-4-carbonyl)Imidazo[1,2-a]pyridin-8-yl)phenyl)benzamide (18)

White solid; Yield: 70.9%; *m.p.*: 2 6 8 ∼ 270 °C; ^1^H NMR (400 MHz, DMSO-*d_6_*) *δ* 10.49 (s, 1H), 8.40 (s, 1H), 8.37 (s, 1H), 8.20 (d, *J* = 7.7 Hz, 2H), 7.94 (d, *J* = 7.9 Hz, 2H), 7.90 − 7.82 (m, 2H), 7.64 (d, *J* = 5.4 Hz, 1H), 7.54 − 7.48 (m, 2H), 4.34 − 4.24 (m, 2H), 3.74 − 3.65 (m, 6H), 2.39 (s, 3H). HRMS (ESI) calculated for C_26_H_24_FN_4_O_3_ [M + H]^+^ m/z 459.1832, found: 459.1937.

##### N,N-Diethyl-3–(6-methyl-2-(morpholine-4-carbonyl)Imidazo[1,2-a]pyridin-8-yl)benzenesulfonamide (19)

White solid; Yield: 72.2%; *m.p.*: 1 7 0 ∼ 173 °C; ^1^H NMR (400 MHz, DMSO-*d_6_*) *δ* 8.89 (s, 1H), 8.48 (s, 1H), 8.42 (s, 1H), 8.34 (d, *J* = 7.6 Hz, 1H), 7.87 (d, *J* = 7.6 Hz, 1H), 7.77 (t, *J* = 7.7 Hz, 1H), 7.68 (s, 1H), 4.41 − 4.31 (m, 2H), 3.75 − 3.63 (m, 6H), 3.23 (q, *J* = 6.7 Hz, 4H), 2.40 (s, 3H), 1.09 (t, *J* = 6.9 Hz, 6H). HRMS (ESI) calculated for C_23_H_29_N_4_O_4_S [M + H]^+^ m/z 457.1910, found: 457.1897.

##### (6-Methyl-8-(pyridin-3-yl)Imidazo[1,2-a]pyridin-2-yl)(morpholino)methanone (20)

White solid; Yield: 77.5%; *m.p.*: 1 9 5 ∼ 198 °C; ^1^H NMR (400 MHz, DMSO-*d_6_*) *δ* 9.30 (s, 1H), 8.64 (d, *J* = 3.4 Hz, 1H), 8.53 (d, *J* = 7.9 Hz, 1H), 8.46 (s, 1H), 8.39 (s, 1H), 7.61 (s, 1H), 7.57 (dd, *J* = 7.6, 4.9 Hz, 1H), 4.26 − 4.18 (s, 2H), 3.72 − 3.62 (s, 6H), 2.39 (s, 3H). HRMS (ESI) calculated for C_18_H_19_N_4_O_2_ [M + H]^+^ m/z 323.1508, found: 323.1541.

##### (8–(6-Methoxypyridin-3-yl)-6-methylImidazo[1,2-a]pyridin-2-yl)(morpholino)methanone (21)

White solid; Yield: 50.2%; ^1^H NMR (600 MHz, DMSO-*d*_6_) δ 8.92 (d, *J* = 2.2 Hz, 1H), 8.44 (dd, *J* = 8.7, 2.5 Hz, 1H), 8.37 (t, *J* = 1.1 Hz, 1H), 8.34 (s, 1H), 7.48 (d, *J* = 1.4 Hz, 1H), 6.96 (d, *J* = 8.7 Hz, 1H), 4.25 − 4.15 (m, 2H), 3.93 (s, 3H), 3.71 − 3.59 (m, 6H), 2.34 (s, 3H). ^13^C NMR (151 MHz, DMSO) δ 163.9, 162.8, 147.0, 141.1, 139.9, 139.5, 126.7, 125.5, 125.3, 124.2, 123.1, 117.5, 110.5, 67.0, 66.8, 53.8, 47.6, 43.0, 18.1. HRMS (ESI) calculated. for C_19_H_21_O_3_N_4_ [M + H]^+^ m/z 353.1614, found: 353.1619.

##### (8–(4-Fluorophenyl)-6-methylImidazo[1,2-a]pyridin-2-yl)(pyrrolidin-1-yl)methanone (22)

White solid; Yield: 78.9%; *m.p.*: 1 8 8 ∼ 191 °C; ^1^H NMR (400 MHz, CDCl_3_) *δ* 8.19 (s, 1H), 8.12 (t, *J* = 7.0 Hz, 2H), 7.97 (s, 1H), 7.29 − 7.17 (m, 3H), 4.20 (t, *J* = 6.7 Hz, 2H), 3.74 (t, *J* = 6.8 Hz, 2H), 2.43 (s, 3H), 2.06 − 2.00 (m, 2H), 1.99 − 1.93 (m, *J* = 4.5 Hz, 2H). HRMS (ESI) calculated for C_19_H_19_FN_3_O [M + H]^+^ m/z 324.1512, found: 324.1500.

##### (8–(3-Fluorophenyl)-6-methylImidazo[1,2-a]pyridin-2-yl)(pyrrolidin-1-yl)methanone (23)

White solid; Yield: 81.4%; *m.p.*: 1 5 3 ∼ 155 °C; ^1^H NMR (400 MHz, DMSO-*d_6_*) *δ* 9.34 (s, 1H), 8.64 (s, 1H), 8.55 (d, *J* = 7.4 Hz, 1H), 8.45 (s, 1H), 8.32 (s, 1H), 7.62 (s, 1H), 7.60 − 7.52 (m, 1H), 4.03 (t, *J* = 6.7 Hz, 2H), 3.63 (t, *J* = 6.8 Hz, 2H), 2.39 (s, 3H), 1.70 − 1.64 (m, 2H), 1.60 − 1.54 (m, 2H). HRMS (ESI) calculated for C_19_H_19_FN_3_O [M + H]^+^ m/z 324.1512, found: 321.1741.

##### (6-Methyl-8-(pyridin-3-yl)Imidazo[1,2-a]pyridin-2-yl)(pyrrolidin-1-yl)methanone (24)

White solid; Yield: 79.7%; *m.p.*: 1 5 9 ∼ 162 °C; ^1^H NMR (400 MHz, DMSO-*d_6_*) *δ* 9.39 (s, 1H), 8.66 (d, *J* = 3.4 Hz, 1H), 8.59 (d, *J* = 7.9 Hz, 1H), 8.48 (s, 1H), 8.42 (s, 1H), 7.63 (s, 1H), 7.61 − 7.56 (m, 1H), 4.05 (t, *J* = 6.4 Hz, 2H), 3.55 (t, *J* = 6.6 Hz, 2H), 2.40 (s, 3H), 1.99 − 1.93 (m, 2H), 1.90 − 1.85 (m, 2H). HRMS (ESI) calculated for C_18_H_19_N_4_O [M + H]^+^ m/z 307.1559, found: 307.1658.

##### (8–(6-Methoxypyridin-3-yl)-6-methylImidazo[1,2-a]pyridin-2-yl)(pyrrolidin-1-yl)methanone (25)

Gray white solid; Yield: 46.5%; ^1^H NMR (600 MHz, DMSO-*d*_6_) δ 9.01 (d, *J* = 2.4 Hz, 1H), 8.48 (dd, *J* = 8.7, 2.5 Hz, 1H), 8.37 (t, *J* = 1.5 Hz, 1H), 8.35 (s, 1H), 7.49 (d, *J* = 1.3 Hz, 1H), 6.96 (d, *J* = 8.7 Hz, 1H), 4.01 (t, *J* = 6.8 Hz, 2H), 3.93 (s, 3H), 3.52 (t, *J* = 6.9 Hz, 2H), 2.34 (d, *J* = 1.3 Hz, 3H), 1.96 − 1.88 (m, 2H), 1.87 − 1.80 (m, 2H). ^13^C NMR (151 MHz, DMSO-*d*_6_) δ 163.9, 162.1, 147.1, 141.3, 141.1, 139.4, 126.4, 125.5, 125.4, 124.2, 123.1, 117.0, 110.5, 53.9, 48.7, 47.0, 26.8, 23.8, 18.1. HRMS (ESI) calculated. for C_19_H_21_O_2_N_4_ [M + H]^+^ m/z 337.1665, found: 337.1674.

##### 3-Fluoro-N-(4–(6-methyl-2-(pyrrolidine-1-carbonyl)Imidazo[1,2-a]pyridin-8-yl)phenyl)benzamide (26)

White solid; Yield: 68.6%; *m.p.*: 2 6 3 ∼ 265 °C; ^1^H NMR (400 MHz, DMSO-*d_6_*) *δ* 10.48 (s, 1H), 8.43 − 8.25 (m, 4H), 8.00 − 7.90 (m, 2H), 7.65 − 7.46 (m, 5H), 4.10 (t, *J* = 6.8 Hz, 2H), 3.55 (t, *J* = 6.8 Hz, 2H), 2.42 (s, 3H), 1.96 − 1.88 (m, 4H); HRMS (ESI) calculated for C_26_H_24_FN_4_O_2_ [M + H]^+^ m/z 443.1883, found: 443.1995.

##### (6-Methyl-8–(3-(morpholinosulfonyl)phenyl)Imidazo[1,2-a]pyridin-2-yl)(pyrrolidin-1-yl)methanone (27)

White solid; Yield: 71.1%; *m.p.*: 1 7 0 ∼ 172 °C; ^1^H NMR (400 MHz, DMSO-*d_6_*) *δ* 8.97 (s, 1H), 8.51 − 8.45 (m, 2H), 8.43 (s, 1H), 7.87 − 7.80 (m, 2H), 7.72 (s, 1H), 4.13 (t, *J* = 6.4 Hz, 2H), 3.70 − 3.62 (m, 4H), 3.55 (t, *J* = 6.6 Hz, 2H), 2.98 − 2.90 (m, 4H), 2.41 (s, 2H), 2.00 − 1.88 (m, 3H), 1.88 − 1.83 (m, 2H). HRMS (ESI) calculated for C_23_H_27_N_4_O_4_S [M + H]^+^ m/z 455.1753, found: 455.1736.

##### N,N-Diethyl-3–(6-methyl-2-(pyrrolidine-1-carbonyl)Imidazo[1,2-a]pyridin-8-yl)benzenesulfonamide (28)

White solid; Yield: 73.7%; *m.p.*: 1 6 4 ∼ 167 °C; ^1^H NMR (400 MHz, DMSO-*d_6_*) *δ* 8.83 (s, 1H), 8.48 (s, 1H), 8.40 − 8.35 (m, 2H), 7.88 (d, *J* = 7.2 Hz, 1H), 7.77 (t, *J* = 7.6 Hz, 1H), 7.66 (s, 1H), 5.13 (s, 1H), 4.53 (s, 1H), 3.30 − 3.22 (m, 5H), 2.46 − 2.38 (m, 2H), 1.76 − 1.68 (m, 2H), 1.14 − 1.02 (m, 7H), 0.96 (d, *J* = 5.3 Hz, 3H). HRMS (ESI) calculated for C_23_H_29_N_4_O_3_S [M + H]^+^ m/z 441.1960, found: 441.1976.

##### (8–(3-Fluorophenyl)-6-methylImidazo[1,2-a]pyridin-2-yl)(4-methylpiperidin-1-yl)methanone (29)

White solid; Yield: 85.9%; ^1^H NMR (400 MHz, DMSO-*d_6_*) *δ* 8.44 (s, 1H), 8.31 (s, 1H), 8.18 (d, *J* = 11.3 Hz, 1H), 8.03 (d, *J* = 7.7 Hz, 1H), 7.62 (s, 1H), 7.60 − 7.54 (m, 1H), 7.30 (t, *J* = 8.2 Hz, 1H), 5.03 (s, 1H), 4.51 (s, 1H), 3.14 (s, 1H), 2.78 (s, 1H), 2.39 (s, 3H), 1.76 − 1.68 (m, 3H), 1.24 − 1.16 (m, 2H), 0.96 (d, *J* = 5.5 Hz, 3H). HRMS (ESI) calculated for C_21_H_23_FN_3_O [M + H]^+^ m/z 352.1825, found: 352.1854.

##### (6-Methyl-8-(pyridin-4-yl)Imidazo[1,2-a]pyridin-2-yl)(4-methylpiperidin-1-yl)methanone (30)

White solid; Yield: 76.5%; *m.p.*: 1 7 4 ∼ 177 °C; ^1^H NMR (400 MHz, DMSO-*d_6_*) *δ* 8.72 (d, *J* = 5.4 Hz, 2H), 8.50 (s, 1H), 8.33 (s, 1H), 8.23 (d, *J* = 5.4 Hz, 2H), 7.74 (s, 1H), 4.97 (s, 1H), 4.52 (s, 1H), 3.15 (s, 1H), 2.78 (s, 1H), 2.40 (s, 3H), 1.76 − 1.66 (m, 3H), 1.18 − 1.10 (m, 2H), 0.96 (d, *J* = 5.8 Hz, 3H). HRMS (ESI) calculated for C_20_H_23_N_4_O [M + H]^+^ m/z 335.1872, found: 335.1955.

##### (6-Methyl-8-(pyridin-3-yl)Imidazo[1,2-a]pyridin-2-yl)(4-methylpiperidin-1-yl)methanone (31)

White solid; Yield: 79.5%; m.p.: 1 4 4 ∼ 147 °C; ^1^H NMR (400 MHz, DMSO-*d_6_*) *δ* 8.72 (d, *J* = 5.4 Hz, 2H), 8.50 (s, 1H), 8.33 (s, 1H), 8.23 (d, *J* = 5.4 Hz, 2H), 7.74 (s, 1H), 4.97 (s, 1H), 4.52 (s, 1H), 3.15 (s, 1H), 2.78 (s, 1H), 2.40 (s, 3H), 1.77 − 1.67 (m, 3H), 1.17 − 1.11 (m, 2H), 0.96 (d, *J* = 5.8 Hz, 3H). HRMS (ESI) calculated for C_20_H_23_N_4_O [M + H]^+^ m/z 335.1872, found: 335.1959.

##### N,N-Diethyl-3–(6-methyl-2–(4-methylpiperidine-1-carbonyl)Imidazo[1,2-a]pyridin-8-yl)benzenesulfonamide (32)

White solid; Yield: 73.9%; *m.p.*: 1 3 1 ∼ 133 °C; ^1^H NMR (400 MHz, DMSO-*d_6_*) *δ* 8.81 (s, 1H), 8.47 (s, 1H), 8.39 − 8.33 (m, 2H), 7.87 (d, *J* = 6.4 Hz, 1H), 7.76 (t, *J* = 7.6 Hz, 1H), 7.65 (s, 1H), 4.13 (t, *J* = 6.7 Hz, 2H), 3.64 (t, *J* = 6.8 Hz, 2H), 3.29 − 3.21 (m, 5H), 2.41 (s, 3H), 1.69 − 1.63 (m, 2H), 1.61 − 1.55 (m, 4H), 1.14 − 1.06 (m, 7H); ^13^C NMR (100 MHz, DMSO-*d_6_*) *δ* 162.6, 140.9, 140.8, 140.5, 136.9, 132.4, 129.9, 128.0, 127.2, 126.8, 126.4, 125.1, 122.9, 117.2, 42.5, 40.9, 39.1, 31.0, 22.1, 18.1, 14.7. HRMS (ESI) calculated for C_25_H_33_N_4_O_3_S [M + H]^+^ m/z 469.2273, found: 469.2343.

##### 1-Cyclohexyl-3–(3-(6-methyl-2–(4-methylpiperidine-1-carbonyl)Imidazo[1,2-a]pyridin-8-yl)phenyl)urea (33)

White solid; Yield: 75.0%; *m.p.*: 1 6 8 ∼ 170 °C; ^1^H NMR (400 MHz, DMSO-*d_6_*) *δ* 8.50 (s, 1H), 8.39 (s, 1H), 8.29 (s, 1H), 8.25 (s, 1H), 7.59 (s, 1H), 7.44 − 7.32 (m, 3H), 6.21 (s, 1H), 5.08 (s, 1H), 4.51 (s, 1H), 3.11 (s, 1H), 2.76 (s, 1H), 2.37 (s, 3H), 1.86 − 1.78 (m, 3H), 1.73 − 1.65 (m, 4H), 1.55 (s, 1H), 1.36 − 1.28 (m, 2H), 1.27 − 1.21 (m, 7H), 0.98 − 0.90 (m, 4H). ^13^C NMR (100 MHz, DMSO-*d_6_*) *δ* 162.8, 154.9, 141.3, 141.0, 140.7, 137.2, 136.5, 128.9, 127.0, 124.1, 122.8, 121.7, 118.6, 118.1, 116.7, 48.2, 40.9, 38.8, 33.5, 31.1, 25.7, 24.8, 22.1, 18.1. HRMS (ESI) calculated for C_28_H_36_N_5_O_2_ [M + H]^+^ m/z 474.2869, found: 474.2998.

##### N-(2-methoxy-5–(6-methyl-2-(pyrrolidine-1-carbonyl)Imidazo[1,2-a]pyridin-8-yl)pyridin-3-yl)benzenesulfonamide (34)

Light yellow solid; Yield: 49.5%; ^1^H NMR (600 MHz, DMSO-*d*_6_) δ 9.93 (s, 1H), 8.73 − 8.70 (m, 1H), 8.55 (d, *J* = 2.3 Hz, 1H), 8.39 − 8.35 (m, 1H), 8.35 − 8.33 (m, 1H), 7.75 (t, t, *J* = 1.8 Hz, 1H), 7.73 (t, *J* = 1.8 Hz, 1H), 7.64 − 7.58 (m, 1H), 7.55 − 7.50 (m, 2H), 7.43 − 7.41 (m, 1H), 4.03 (t, *J* = 7.3 Hz, 2H), 3.63 (s, 3H), 3.51 (t, *J* = 6.9 Hz, 2H), 2.33 (s, 3H), 1.93 − 1.86 (m, 2H), 1.82 (q, *J* = 6.8 Hz, 2H). ^13^C NMR (151 MHz, DMSO-*d*_6_) δ 162.1, 157.4, 143.4, 141.2, 141.1, 140.9, 134.2, 133.3, 129.4, 127.1, 126.6, 125.6, 124.8, 124.5, 123.0, 120.3, 117.1, 53.9, 48.8, 47.1, 26.8, 23.8, 18.1. HRMS (ESI) calculated. for C_25_H_26_N_5_O_4_S [M + H]^+^ m/z 492.1706, found: 492.1709.

##### 4-Fluoro-N-(2-methoxy-5–(6-methyl-2-(pyrrolidine-1-carbonyl)Imidazo[1,2-a]pyridin-8-yl)pyridin-3-yl)benzenesulfonamide (35)

White solid; Yield: 52.3%; ^1^H NMR (600 MHz, DMSO-*d*_6_) δ 10.01 (s, 1H), 8.76 (d, *J* = 2.2 Hz, 1H), 8.60 (d, *J* = 2.2 Hz, 1H), 8.40 (t, *J* = 1.2 Hz, 1H), 8.36 (s, 1H), 7.85 − 7.79 (m, 2H), 7.47 (d, *J* = 0.9 Hz,1H), 7.43 − 7.36 (m, 2H), 4.05 (t, *J* = 6.8 Hz, 2H), 3.67 (s, 3H), 3.53 (t, *J* = 6.9 Hz, 2H), 2.35 (s, 3H), 1.95 − 1.88 (m, 2H), 1.87 − 1.76 (m, 2H). ^13^C NMR (151 MHz, DMSO-*d*_6_) δ 164.8, 162.1, 157.5, 143. 7, 141.2, 141.1, 137.3, 134.7, 130.3, 126.6, 125.7, 124.7, 124.5, 123.0, 120.0, 117.1, 116.6, 53.9, 48.8, 47.1, 26.8, 23.8, 18.1. HRMS (ESI) calculated. for C_25_H_25_FN_5_O_4_S [M + H]^+^ m/z 510.1611, found: 510.1631.

##### 2,4-Difluoro-N-(2-methoxy-5–(6-methyl-2-(pyrrolidine-1-carbonyl)Imidazo[1,2-a]pyridin-8-yl)pyridin-3-yl)benzenesulfonamide (36)

White solid; Yield: 50.6%; ^1^H NMR (600 MHz, DMSO-*d*_6_) δ 10.27 (s, 1H), 8.83 − 8.81 (m, 1H), 8.63 (d, *J* = 2.2 Hz, 1H), 8.41 − 8.38 (m, 1H), 8.37 − 8.35 (m, 1H), 7.74 (q, *J* = 8.3 Hz, 1H), 7.62 − 7.54 (m, 1H), 7.53 − 7.50 (m, 1H), 7.19 (td, *J* = 8.5, 2.3 Hz, 1H), 4.07 (t, *J* = 6.7 Hz, 2H), 3.66 (s, 3H), 3.53 (t, *J* = 6.9 Hz, 2H), 2.35 (s, 3H), 1.96 − 1.90 (m, 2H), 1.87 − 1.81 (m, 2H). ^13^C NMR (151 MHz, DMSO-*d*_6_) δ 165.8, 161.9, 159.9, 158.4, 144.5, 141.1, 141.0, 136.9, 132.3, 126.5, 125.8, 125.7, 124.5, 124.4, 123.1, 119.3, 117.2, 112.2, 106.2, 53.9, 48.9, 47.1, 26.8, 23.8, 18.1. HRMS (ESI) calculated. for C_25_H_24_F_2_N_5_O_4_S [M + H]^+^ m/z 528.1517, found: 528.1516.

##### 2,4-Difluoro-N-(2-methoxy-5–(6-methyl-2-(morpholine-4-carbonyl)Imidazo[1,2-a]pyridin-8-yl)pyridin-3-yl)benzenesulfonamide (37)

White solid; Yield: 55.2%; ^1^H NMR (600 MHz, DMSO-*d*_6_) δ 10.30 (s, 1H), 8.72 (d, *J* = 2.3 Hz, 1H), 8.62 (d, *J* = 2.1 Hz, 1H), 8.40 (s, 1H), 8.37 (s, 1H), 7.75 (q, *J* = 8.4 Hz, 1H), 7.63 − 7.55 (m, 1H), 7.53 (s, 1H), 7.19 (td, *J* = 8.5, 2.3 Hz, 1H), 4.36 − 4.24 (m, 2H), 3.71 − 3.66 (m, 6H), 3.66 (s, 3H), 2.35 (s, 3H). ^13^C NMR (151 MHz, DMSO-*d*_6_) δ 165.5, 162.5, 159.6, 158.4, 144.4, 140.9, 140.1, 136.9, 132.3, 126.9, 125.7, 125.7, 124.6, 124.4, 123.2, 119.3, 117.9, 112.2, 106.2, 67.1, 66.8, 53.9, 47.5, 43.0, 18.1. HRMS (ESI) calculated. for C_25_H_24_F_2_N_5_O_5_S [M + H]^+^ m/z 544.1466, found: 544.1511.

##### 8–(5-((2,4-Difluorophenyl)sulfonamido)-6-methoxypyridin-3-yl)-6-methyl-N-(2-morpholinoethyl)Imidazo[1,2-a]pyridine-2-carboxamide (38)

White solid; Yield: 51.9%; ^1^H NMR (600 MHz, DMSO-*d*_6_) δ 10.32 (s, 1H), 9.00 (s, 1H), 8.46 (d, *J* = 2.4 Hz, 1H), 8.39 (s, 1H), 8.35 (s, 1H), 8.20 (t, *J* = 5.4 Hz, 1H), 7.77 (q, *J* = 8.1 Hz, 1H), 7.52 − 7.46 (s, 2H), 7.20 (t, *J* = 7.7 Hz, 1H), 3.70 (s, 3H), 3.58 (t, *J* = 4.7 Hz, 4H), 3.44 (q, *J* = 6.4 Hz, 2H), 3.39 − 3.30 (m, 2H), 2.47 − 2.39 (m, 4H), 2.35 (s, 3H). ^13^C NMR (151 MHz, DMSO-*d*_6_) δ 165.5, 162.3, 159.9, 158.0, 144.6, 141.5, 139.7, 135.6, 132.4, 127.0, 125.6, 125.4, 124.8, 124.4, 123.1, 119.9, 115.1, 112.2, 106.3, 66.6, 57.6, 53.8, 53.6, 35.8, 18.1. HRMS (ESI) calculated. for C_27_H_29_F_2_N_6_O_5_S [M + H]^+^ m/z 587.1888, found: 587.1896.

##### 8–(5-((2,4-Difluorophenyl)sulfonamido)-6-methoxypyridin-3-yl)-6-methyl-N-(3-morpholinopropyl)Imidazo[1,2-a]pyridine-2-carboxamide (39)

White solid; Yield: 49.7%; ^1^H NMR (600 MHz, DMSO-*d*_6_) δ 8.88 (d, *J* = 2.2 Hz, 1H), 8.51 (d, *J* = 2.2 Hz, 1H), 8.39 (t, *J* = 1.4 Hz, 1H), 8.34 (s, 1H), 8.24 (t, *J* = 6.0 Hz, 1H), 7.79 − 7.74 (m, 1H), 7.60 − 7.53 (m, 1H), 7.48 (d, *J* = 1.2 Hz, 1H), 7.19 (td, *J* = 8.5, 2.3 Hz, 1H), 3.71 (s, 3H), 3.52 (t, *J* = 4.6 Hz, 4H), 3.36 (q, *J* = 6.7 Hz, 2H), 2.40 − 2.32 (m, 9H), 1.75 − 1.66 (m, 2H). ^13^C NMR (151 MHz, DMSO-*d*_6_) δ 165.5, 162.5, 159.9, 157.9, 144.1, 141.5, 139.9, 135.4, 132.3, 127.0, 125.7, 125.5, 124.8, 124.6, 123.1, 120.1, 115.1, 112.3, 106.3, 66.4, 56.6, 53.9, 53.7, 37.6, 26.4, 18.1. HRMS (ESI) calculated. for C_28_H_31_F_2_N_5_O_5_S [M + H]^+^ m/z 601.2045, found: 601.2063.

##### 8–(5-((4-Fluorophenyl)sulfonamido)-6-methoxypyridin-3-yl)-6-methyl-N-(2-morpholinoethyl)Imidazo[1,2-a]pyridine-2-carboxamide (40)

White solid; Yield: 50.9%; ^1^H NMR (600 MHz, Chloroform-*d*) δ 9.11 (d, *J* = 2.1 Hz, 1H), 8.55 (d, *J* = 2.1 Hz, 1H), 8.12 (s, 1H), 8.09 (t, *J* = 6.1 Hz, 1H), 7.97 − 7.95 (m, 1H), 7.94 − 7.87 (m, 2H), 7.29 (s, 1H), 7.11 − 7.05 (m, 2H), 3.87 (s, 3H), 3.70 (t, *J* = 4.6 Hz, 4H), 3.62 (q, *J* = 6.3 Hz, 2H), 2.64 (t, *J* = 6.6 Hz, 2H), 2.57 − 2.47 (m, 4H), 2.41 (s, 3H). ^13^C NMR (151 MHz, DMSO-*d*_6_) δ 164.8, 162.4, 157.0, 143.6, 141.5, 139.7, 137.0, 133.5, 130.3, 126.8, 125.3, 124.8, 124.5, 123.1, 120.4, 116.7, 115.0, 66.6, 57.6, 53.9, 53.6, 35.8, 18.1. HRMS (ESI) calculated. for C_27_H_30_FN_6_O_5_S [M + H]+ m/z 569.1982, found: 569.1980.

##### 8–(5-((4-Fluorophenyl)sulfonamido)-6-methoxypyridin-3-yl)-6-methyl-N-(3-morpholinopropyl)Imidazo[1,2-a]pyridine-2-carboxamide (41)

Light yellow solid; Yield: 55.2%. ^1^H NMR (600 MHz, DMSO-*d*_6_) δ 10.08 (s, 1H), 8.78 (d, *J* = 2.2 Hz, 1H), 8.69 (d, *J* = 2.2 Hz, 1H), 8.40 (t, *J* = 1.5 Hz, 1H), 8.36 (s, 1H), 8.24 (t, *J* = 6.0 Hz, 1H), 7.88 − 7.83 (m, 2H), 7.49 (d, *J* = 1.3 Hz, 1H), 7.44 − 7.38 (m, 2H), 3.71 (s, 3H), 3.49 (t, *J* = 4.5 Hz, 4H), 3.36 (q, *J* = 6.7 Hz, 2H), 2.35 (d, *J* = 6.1 Hz, 3H), 2.34 − 2.23 (m, 6H), 1.71 − 1.64 (m, 2H). ^13^C NMR (151 MHz, DMSO-*d*_6_) δ 164.81, 162.4, 156.8, 143.2, 141.6, 139.8, 137.1, 133.4, 130.2, 126.8, 125.4, 124.8, 124.6, 123.1, 120.5, 116.7, 115.1, 66.5, 56.6, 53.9, 53.7, 37.6, 26.5, 18.1. HRMS (ESI) calculated. for C_28_H_32_FN_6_O_5_S [M + H]^+^ m/z 583.2139, found: 583.2141.

##### 4-Fluoro-N-(2-methoxy-5–(6-methyl-2-(morpholine-4-carbonyl)Imidazo[1,2-a]pyridin-8-yl)pyridin-3-yl)benzenesulfonamide (42)

White solid; Yield: 60.8%; ^1^H NMR (600 MHz, DMSO-*d*_6_) δ 10.05 (s, 1H), 8.66 (d, *J* = 2.2 Hz, 1H), 8.63 (d, *J* = 2.2 Hz, 1H), 8.40 (d, J = 1.7 Hz, 1H), 8.37 (s, 1H), 7.84 (dd, J = 8.7, 5.2 Hz, 2H), 7.50 − 7.48 (m, 1H), 7.44 − 7.37 (m, 2H), 4.35 − 4.25 (m, 2H), 3.74 − 3.62 (m, 9H), 2.35 (s, 3H). ^13^C NMR (151 MHz, DMSO-*d*_6_) δ 164.8, 162.7, 157.3, 143.4, 140.9, 140.0, 137.2, 134.6, 130.3, 126.9, 125.6, 124.6, 124.5, 123.1, 120.1, 117.8, 116.6, 67.1, 66.8, 53.9, 47.6, 43.0, 18.1. HRMS (ESI) calculated. for C_25_H_25_FN_5_O_5_S [M + H]^+^ m/z 526.1560, found: 526.1552.

##### 4-Fluoro-N-(2-methoxy-5–(6-methyl-2–(4-methylpiperidine-1-carbonyl)Imidazo[1,2-a]pyridin-8-yl)pyridin-3-yl)benzenesulfonamide (43)

White solid; Yield: 50.4%; ^1^H NMR (600 MHz, DMSO-*d*_6_) δ 10.02 (s, 1H), 8.70 (t, *J* = 2.1 Hz, 1H), 8.62 − 8.57 (m, 1H), 8.41 − 8.35 (m, 1H), 8.28 (d, *J* = 1.3 Hz, 1H), 7.88 − 7.81 (m, 2H), 7.50 − 7.44 (m, 1H), 7.39 (t, *J* = 8.9 Hz, 2H), 4.95 (d, *J* = 12.7 Hz, 1H), 4.50 (d, *J* = 12.5 Hz, 1H), 3.67 (s, 3H), 3.12 (t, *J* = 12.2 Hz, 1H), 2.75 (t, *J* = 12.2 Hz, 1H), 2.38 − 2.30 (m, 3H), 1.68 (s, 3H), 1.22 − 1.10 (m, 2H), 0.92 (d, *J* = 6.3 Hz, 3H). ^13^C NMR (151 MHz, DMSO-*d*_6_) δ 164.8, 162.8, 157.3, 143.5, 140.9, 140.6, 137.2, 134.3, 132.0, 131.9, 130.3, 125.7, 124.5, 122.9, 120.1, 116.7, 116.5, 53.9, 47.0, 42.8, 35.1, 34.2, 31.1, 22.2, 18.1. HRMS (ESI) calculated. for C_27_H_29_FN_5_O_4_S [M + H]^+^ m/z 538.1924, found: 538.1952.

##### 4-Fluoro-N-(2-methoxy-5–(6-methyl-2–(4-methylpiperazine-1-carbonyl)Imidazo[1,2-a]pyridin-8-yl)pyridin-3-yl)benzenesulfonamide (44)

White solid; Yield: 59.9%; ^1^H NMR (600 MHz, DMSO-*d*_6_) δ 10.08 (s, 1H), 8.66 (d, *J* = 2.2 Hz, 1H), 8.60 (d, *J* = 2.2 Hz, 1H), 8.40 (t, *J* = 1.4 Hz, 1H), 8.34 (s, 1H), 7.87 − 7.81 (m, 2H), 7.47 (s, 1H), 7.43 − 7.36 (m, 2H), 4.25 − 4.15 (m, 2H), 3.73 − 3.63 (m, 5H), 2.40 (t, *J* = 5.1 Hz, 4H), 2.35 (s, 3H), 2.21 (s, 3H). ^13^C NMR (151 MHz, DMSO-*d*_6_) δ 164.7, 162.7, 157.3, 143.1, 140.9, 140.2, 137.5, 134.1, 130.2, 126.8, 125.6, 124.7, 124.5, 123.0, 120.6, 117.4, 116.5, 55.6, 54.9, 53.9, 46.7, 46.0, 42.3, 18.1. HRMS (ESI) calculated. for C_26_H_28_FN_6_O_4_S [M + H]^+^ m/z 539.1875, found: 539.1877.

##### 6-Chloro-8–(5-((2,4-difluorophenyl)sulfonamido)-6-methoxypyridin-3-yl)-N-(3-morpholinopropyl)Imidazo[1,2-a]pyridine-2-carboxamide (45)

White solid; Yield: 47.9%; ^1^H NMR (600 MHz, Chloroform-*d*) δ 8.92 (d, *J* = 2.1 Hz, 1H), 8.46 (d, *J* = 2.1 Hz, 1H), 8.20 (d, *J* = 1.7 Hz, 1H), 8.17 (s, 1H), 8.03 (t, *J* = 5.9 Hz, 1H), 7.88 − 7.81 (m, 1H), 7.33 (d, *J* = 1.7 Hz, 1H), 6.97 − 6.90 (m, 1H), 6.89 − 6.85 (m, 1H), 3.96 (s, 3H), 3.64 (t, *J* = 4.4 Hz, 4H), 3.56 (q, *J* = 6.7 Hz, 2H), 2.48 (t, *J* = 7.0 Hz, 3H), 2.44 (s, 3H), 1.89 − 1.81 (m, 2H). ^13^C NMR (151 MHz, Chloroform-*d*) δ 166.2, 162.4, 159.9, 154.9, 141.8, 141.3, 140.9, 132.4, 129.3, 126.9, 124.4, 123.8, 123.4, 123.3, 121.7, 119.9, 114.8, 112.0, 105.8, 66.7, 56.8, 54.2, 53.6, 37.9, 26.2. HRMS (ESI) calculated. for C_27_H_28_ClF_2_N_6_O_5_S [M + H]^+^ m/z 621.1498, found: 621.1503.

##### 4-Fluoro-N-(2-methoxy-5–(2-(4-methylpiperidine-1-carbonyl)Imidazo[1,2-a]pyridin-8-yl)pyridin-3-yl)benzenesulfonamide (46)

White solid; Yield: 58.1%; ^1^H NMR (600 MHz, DMSO-*d*_6_) δ 10.03 (s, 1H), 8.68 (d, *J* = 2.2 Hz, 1H), 8.63 − 8.61 (m, 1H), 8.59 (d, *J* = 6.7 Hz, 1H), 8.38 (s, 1H), 7.88 − 7.83 (m, 2H), 7.62 − 7.58 (m, 1H), 7.41 − 7.37 (m, 2H), 7.10 − 7.05 (m, 1H), 4.89 (d, *J* = 11.0 Hz, 1H), 4.51 (d, *J* = 10.2 Hz, 1H), 3.68 (s, 3H), 3.13 (s, 1H), 2.76 (s, 1H), 1.68 (d, *J* = 10.7 Hz, 3H), 1.19 − 1.05 (m, 2H), 0.95 − 0.85 (m, 3H). ^13^C NMR (151 MHz, DMSO-*d*_6_) δ 164.8, 162.9, 157.2, 143.4, 141.8, 140.7, 137.2, 134.0, 130.3, 127.2, 125.7, 125.3, 123.7, 120.2, 117.0, 116.6, 113.8, 53.9, 47.1, 42.7, 35.0, 34.2, 31.0, 22.2. HRMS (ESI) calculated. for C_26_H_27_FN_5_O_4_S [M + H]^+^ m/z 524.1768, found: 524.1852.

### Pharmacology

#### *In vitro* enzymatic assay

*In vitro* enzymatic assay of compounds **15–46** against PI3K*α* was evaluated by ADP-Glo™ Kinase Assay. Briefly, the compound, PI3K enzyme (PI3K*α* from Invitrogen), the PIP2 (Life Technologies) substrate, and ATP (25 μM, Sigma) were diluted in kinase buffer to the indicated concentrations. The assay plate was covered and incubated at room temperature for 1 h. Then, the Kinase-Glo reagent (Promega) was added to the PI3K*α* plate to stop the reaction, mixed briefly with centrifuge, shaken slowly on the shaker and then equilibrate for 120 min. Finally, add Kinase detection reagent to each well, shake 1 min, equilibrate for 30 min before reading on a plate reader for luminescence. The data were collected on Envision and presented in Excel. IC_50_ values were calculated from the inhibition curves.

#### Cell proliferation assays

All target compounds were evaluated for antiproliferative potency against SKOV-3, T47D,NCI-H1975, NCI-H460,and MCF-7 tumour cell lines using a CellTiter-Glo^®^ Luminescent Cell Viability Assay. The human tumour cell lines used were obtained from the ATCC or Shanghai ZhongQiaoXinZhou Biotech. All the mediums and FBS were Gibco. T47D, H1975 and NCI-H460 tumour cells were cultured in RPMI1640 medium supplemented with 10% FBS. SKOV-3 was cultured in McCoy’s 5 A medium supplemented with 10%FBS. The day before treatment with compounds, cells were seeded at a density of 2000 or 5000 in each well of a 96-well plate. The tumour cells were then treated with 3-fold serial diluted compound or DMSO control in the incubator at 37 °C and 5% CO_2_ for 3 days, prior to the addition of CellTiter-Glo reagents (Promega) and reading of luminescence using a PerkinElmer Envision plate reader. Data were analysed using GraphPad Prism 7.0.

#### Cell cycle and apoptosis analysis

For cell cycle analysis, T47D cell (1 × 10^6^) were seeded in 6-well plates overnight and treated with different concentrations of compound **35** and DMSO control on the next day. After 24 h, the cells were collected by EDTA-free trypsinization, centrifuged at 1500 rpm for 5 min, and fixed in 75% ethanol at −20 °C for 1 h. The cells were then centrifuged at 2000 rpm for 5 min, resuspended in 500 µL PI/Rnase Staining Buffer, incubated for 30 min at room temperature, and analysed using flow cytometry. The data were analysed using Modfit software. For cell apoptosis analysis, T47D cell were seeded in 6 well-plates and treated with DMSO or compound **35** for 24 h. The treated cells were collected and washed with cold PBS for three times, resuspended in 185 µL binding buffer containing 5 μL Annexin V-FITC Apoptosis Detection Kit and 10 μL 7-AAD Viability Staining Solution for 15 min at room temperature in the dark. Apoptotic cells were analysed by flow cytometer.

#### Liver microsomal stability assay

The metabolic stability of compound **35** was determined in human or SD rat liver microsomes, with and without the NADPH regenerating system. Briefly, compound **35** was incubated with microsomes (human microsome, CORNING, lot No. 38296; rat microsome, Xenotech, Lot No. 2110178) (0.5 mg protein/mL) at 1 μM at 37 °C in potassium phosphate buffer (100 mM at pH 7.4 with 10 mM MgCl_2_). The reactions were initiated by adding prewarmed cofactors (1 mmol NADPH). After incubation for different times (0, 5, 10, 20, 30, and 60 min) at 37 °C, cold acetonitrile containing 200 ng/mL tolbutamide and 200 ng/mL labetalol as internal standards was added to precipitate the protein. Then, the samples were centrifuged, and the supernatants were transferred into HPLC water, mixed by plate shaker for 10 min prior to LC − MS/MS analysis.

#### Parallel artificial membrane permeability assay (PAMPA)

10.0 μM donor solution (5% DMSO) was prepared by diluting of working solution with PBS. The donor solution was added to each well of the donor plate, whose PVDF membrane was precoated with 5 µL of 1% lecithin/dodecane mixture. Duplicates were prepared.Then, PBS was added to each well of the PTFE acceptor plate. The donor plate and acceptor plate were combined and incubated for 4 h at room temperature with shaking at 300 rpm. Acceptor samples and donor samples were prepared and analysed by LC-MS/MS.

#### Cyp 450 inhibition assay

Cytochrome P450 inhibition was evaluated in human liver microsomes (0.253 mg/mL, Lot No. 38292) using five specific probe substrates (CYP1A2, 10 μM phenacetin; CYP2D6, 5 μM dextromethorphan; and CYP3A4, 2 μM midazolam; 2C9, 5 μM Diclofenac; 2C19, 30 μM S-mephenytoin) and positive control in the presence of multiple concentrations of the test compound. After pre-warm at 37 °C for 10 min, the reaction was initiated by the addition of of NADPH. The mixture was incubated at 37 °C for 10 min, terminated by the addition of cold stop solution (200 ng/mL tolbutamide and 200 ng/mL labetalol in acetonitrile). The samples were centrifuged, and the supernatants were analysed by LC − MS/MS.

### Molecular docking and dynamics simulation

The protein coordinate (PDB: 4JPS), downloaded from the Protein Data Bank (http://www.rcsb.org/pdb/), was chosen as templates to compare the docking mode among compound **35** bound to PI3K*α*. Molecular docking calculations were conducted using the Dock6 protocol in Yinfo Cloud Platform (http://cloud.yinfotek.com/). Briefly, the structure of compound was built with energy minimisation in MMFF94 force field, and PI3K*α* was assigned hydrogen atoms and partial charges in Amber ff14SB force field and partial charges in Chimaera. The binding pocket of the crystal ligand was assumed to be analogous to that of **35** in PI3Ks. The box centre and the dimensions were thus set. The DOCK 6.7 program was utilised to conduct semiflexible docking with 10 000 different orientations generated. Then, the Grid-based score was calculated for each pose. The image files were generated by Pymol. To investigate the combined stability of inhibitors to PI3K kinase, the MD simulations were conducted on the PI3K kinase in complex with compound **35** by Amber 16 software. The restrained electrostatic potential (RESP) calculated by the Gaussian16 package was used to fit the charges for the inhibitors. The ff14SB force field and the general AMBER force field (gaff2) were used for PI3K kinase and the inhibitors, respectively. Build a solvent octahedral box with a boundary 15 Å away from the protein and use the TIP3P water model to fill the entire octahedral box. 7 Na^+^ ions were added to neutralise the system. The MD simulation protocol comprised the following steps^1^: The limiting potentials of proteins, ligands, and counterions were all restricted by the force constant of 200 kcal/(mol Å), and the energy of the solvent water molecules was minimised to make the water molecules reach a relaxed state. (2) The energy of the system is further minimised. Protein, ligand and ions were subjected to the limiting potential with a force constant of 300 kcal/(molÅ). (3) The restriction potential of the protein backbone was restricted by the force constant of 20 kcal/(mol Å). (4) Then the system was minimised without any restriction. The cpptraj module in AMBER16 was employed for the RMSD calculations.

## Supplementary Material

Supplemental Material
