# Supplementary material for: Design, synthesis and evaluation of 2, 6, 8-substituted Imidazopyridine derivatives as potent PI3Kα inhibitors
Source: J Enzyme Inhib Med Chem. 2023 Jan 17;38(1):2155638. doi: 10.1080/14756366.2022.2155638 (PMC9858543; doi:10.1080/14756366.2022.2155638)
Supplement: Supplemental Material [file IENZ_A_2155638_SM7591.pdf]

Supplementary Material

Design, synthesis and evaluation of 2, 6, 8-substituted Imidazo[1,2-a]pyridine derivatives as potent PI3K*α* inhibitors

Contents:

Figure S1. ^{1}H-NMR spectrum of **15.**

Figure S2. ^{13}C-NMR spectrum of **15.**

Figure S3. ^{1}H-NMR spectrum of **16.**

Figure S4. ^{1}H-NMR spectrum of **17.**

Figure S5. ^{1}H-NMR spectrum of **18.**

Figure S6. ^{1}H-NMR spectrum of **19.**

Figure S7. ^{1}H-NMR spectrum of **20.**

Figure S8. ^{1}H-NMR spectrum of **21.**

Figure S9. ^{13}C-NMR spectrum of **21.**

Figure S10. ^{1}H-NMR spectrum of **22.**

Figure S11. ^{1}H-NMR spectrum of **23.**

Figure S12. ^{1}H-NMR spectrum of **24.**

Figure S13. ^{1}H-NMR spectrum of **25.**

Figure S14. ^{13}C-NMR spectrum of **25.**

Figure S15. ^{1}H-NMR spectrum of **26.**

Figure S16. ^{1}H-NMR spectrum of **27.**

Figure S17. ^{1}H-NMR spectrum of **28.**

Figure S18. ^{1}H-NMR spectrum of **29.**

Figure S19. ^{1}H-NMR spectrum of **30.**

Figure S20. ^{1}H-NMR spectrum of **31.**

Figure S21. ^{1}H-NMR spectrum of **32.**

Figure S22. ^{13}C-NMR spectrum of **32.**

Figure S23. ^{1}H-NMR spectrum of **33.**

Figure S24. ^{13}C-NMR spectrum of **33.**

Figure S25. ^{1}H-NMR spectrum of **34.**

Figure S26. ^{13}C-NMR spectrum of **34.**

Figure S27. ^{1}H-NMR spectrum of **35.**

Information Classification: General

Figure S28. ^{13}C-NMR spectrum of **35.**

Figure S29. ^{1}H-NMR spectrum of **36.**

Figure S30. ^{13}C-NMR spectrum of **36.**

Figure S31. ^{1}H-NMR spectrum of **37.**

Figure S32. ^{13}C-NMR spectrum of **37.**

Figure S33. ^{1}H-NMR spectrum of **38.**

Figure S34. ^{13}C-NMR spectrum of **38.**

Figure S35. ^{1}H-NMR spectrum of **39.**

Figure S36. ^{13}C-NMR spectrum of **39.**

Figure S37. ^{1}H-NMR spectrum of **40.**

Figure S38. ^{13}C-NMR spectrum of **40.**

Figure S39. ^{1}H-NMR spectrum of **41.**

Figure S40. ^{13}C-NMR spectrum of **41.**

Figure S41. ^{1}H-NMR spectrum of **42.**

Figure S42. ^{13}C-NMR spectrum of **42.**

Figure S43. ^{1}H-NMR spectrum of **43.**

Figure S44. ^{13}C-NMR spectrum of **43.**

Figure S45. ^{1}H-NMR spectrum of **44.**

Figure S46. ^{13}C-NMR spectrum of **44.**

Figure S47. ^{1}H-NMR spectrum of **45.**

Figure S48. ^{13}C-NMR spectrum of **45.**

Figure S49. ^{1}H-NMR spectrum of **46.**

Figure S50. ^{13}C-NMR spectrum of **46.**

Information Classification: General

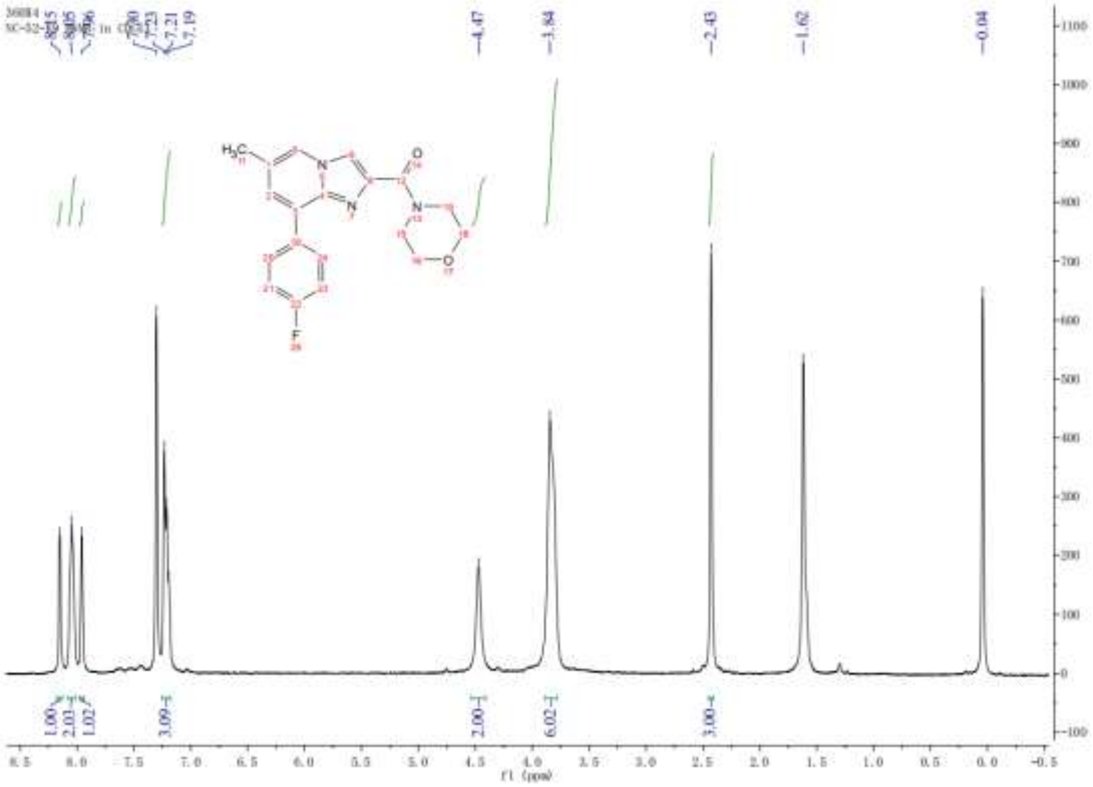

Figure S1. ^{1}H-NMR spectrum of compound **15.**

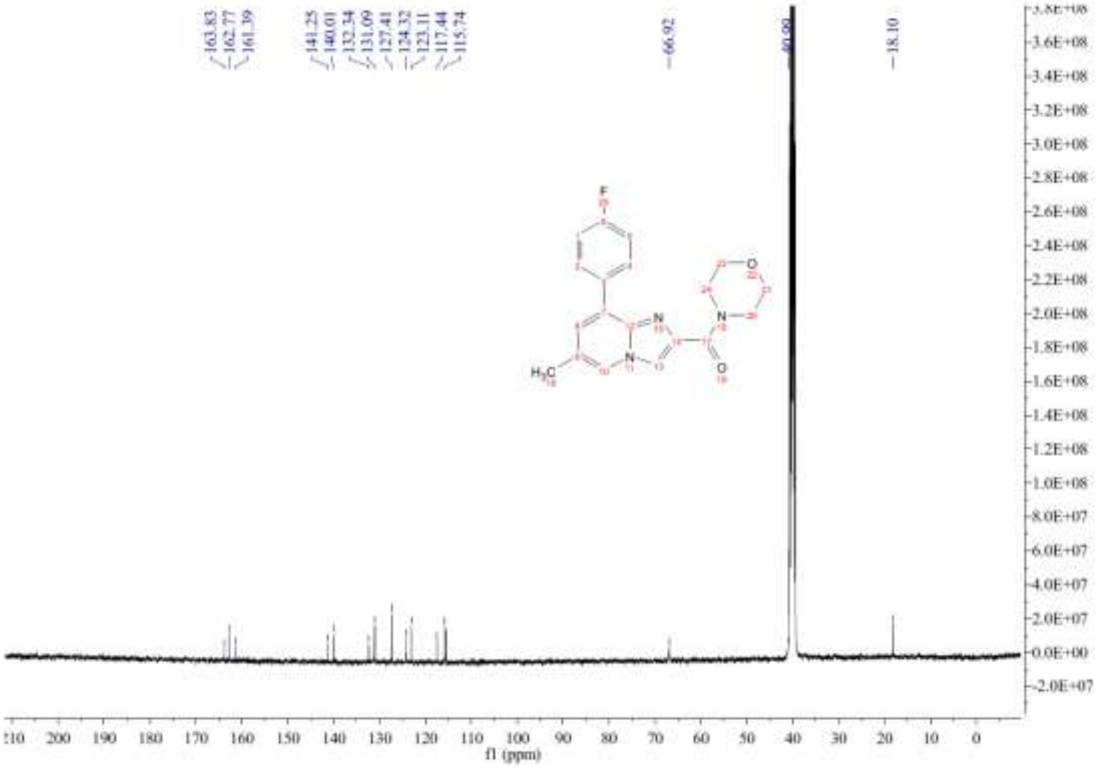

Figure S2. ^{13}C-NMR spectrum of compound **15.**

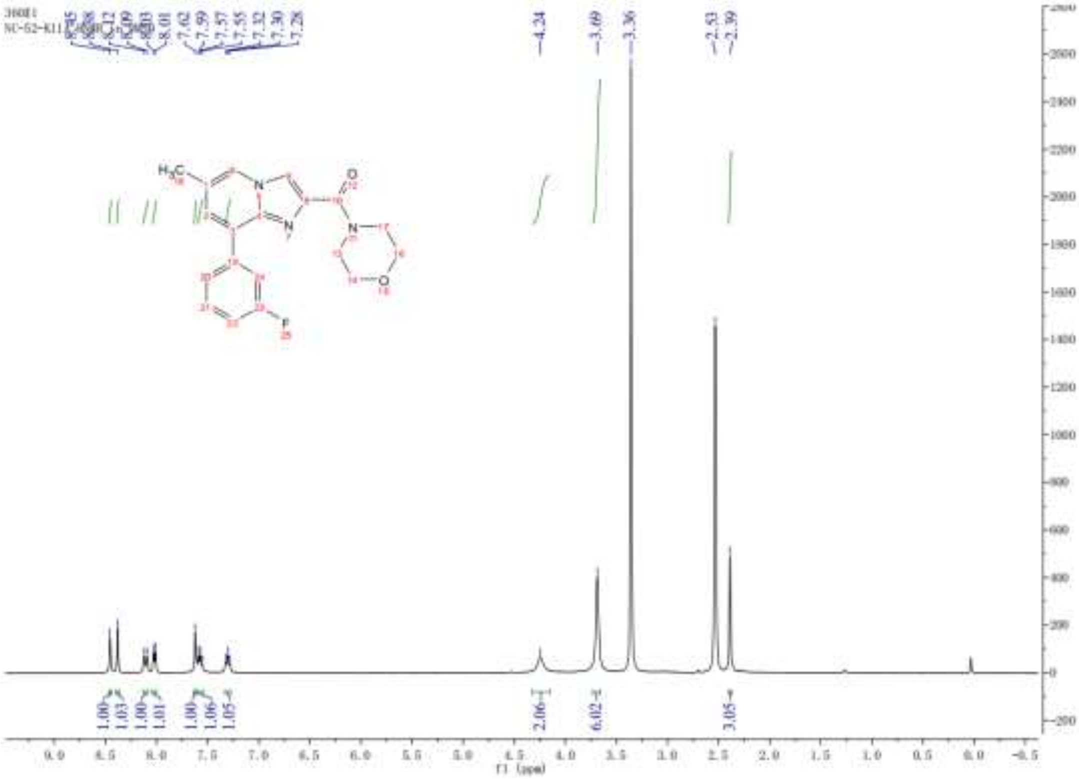

Figure S3. ^{1}H-NMR spectrum of compound **16.**

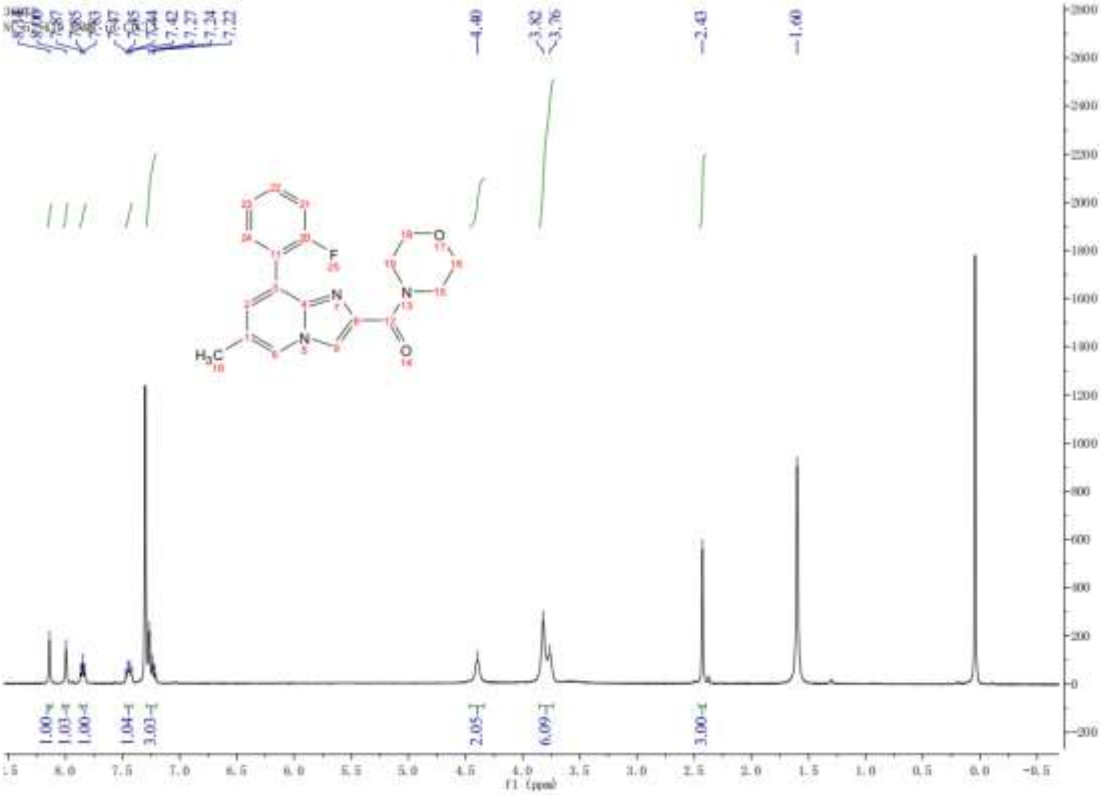

Figure S4. ^{1}H-NMR spectrum of compound **17.**

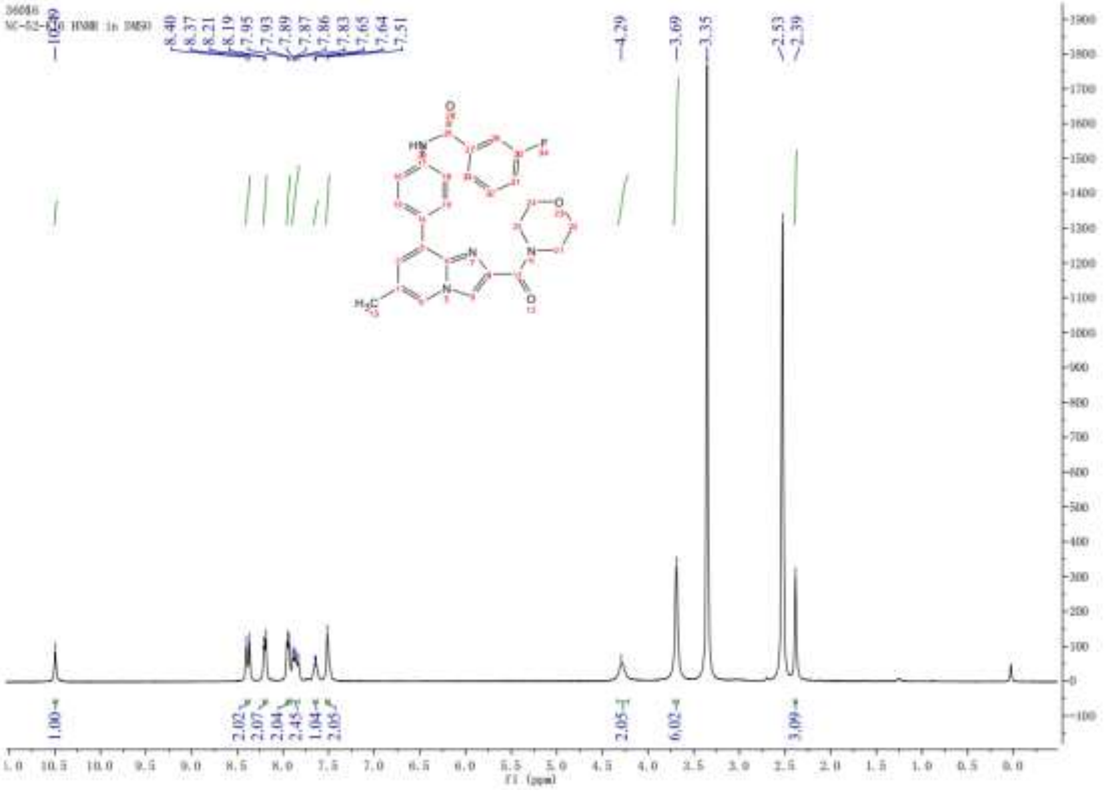

Figure S5. ^{1}H-NMR spectrum of compound **18.**

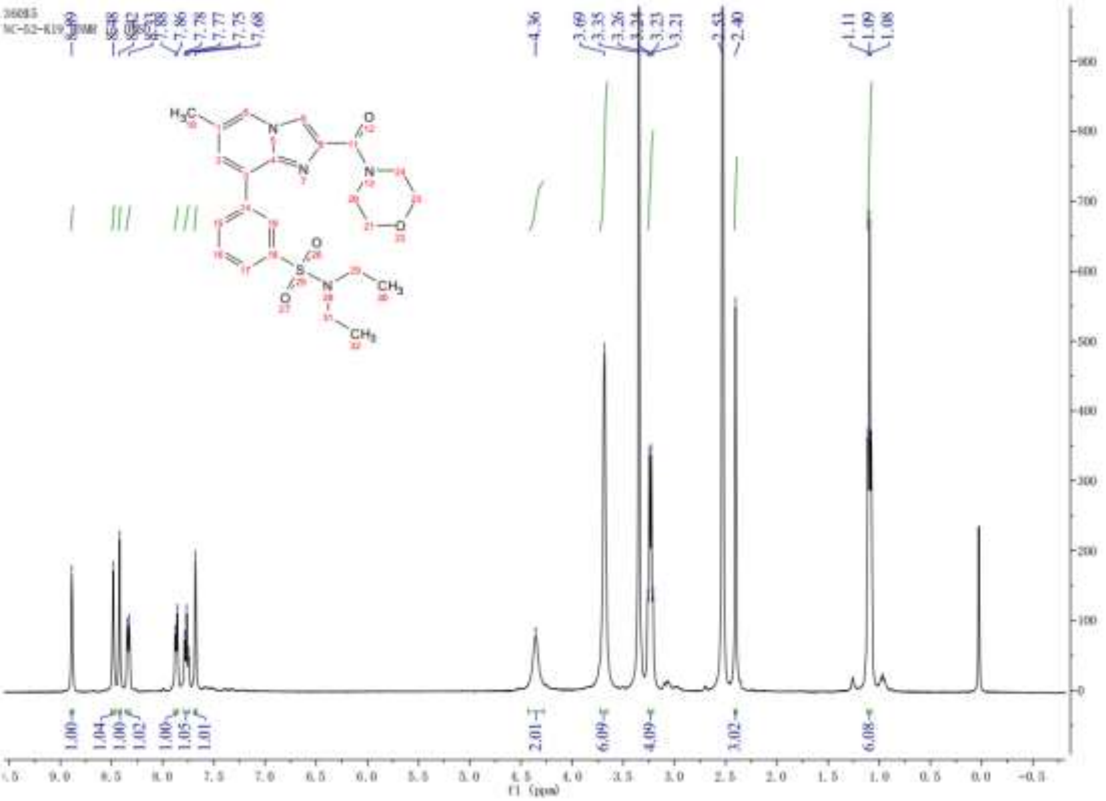

Figure S6. ^{1}H-NMR spectrum of compound **19.**

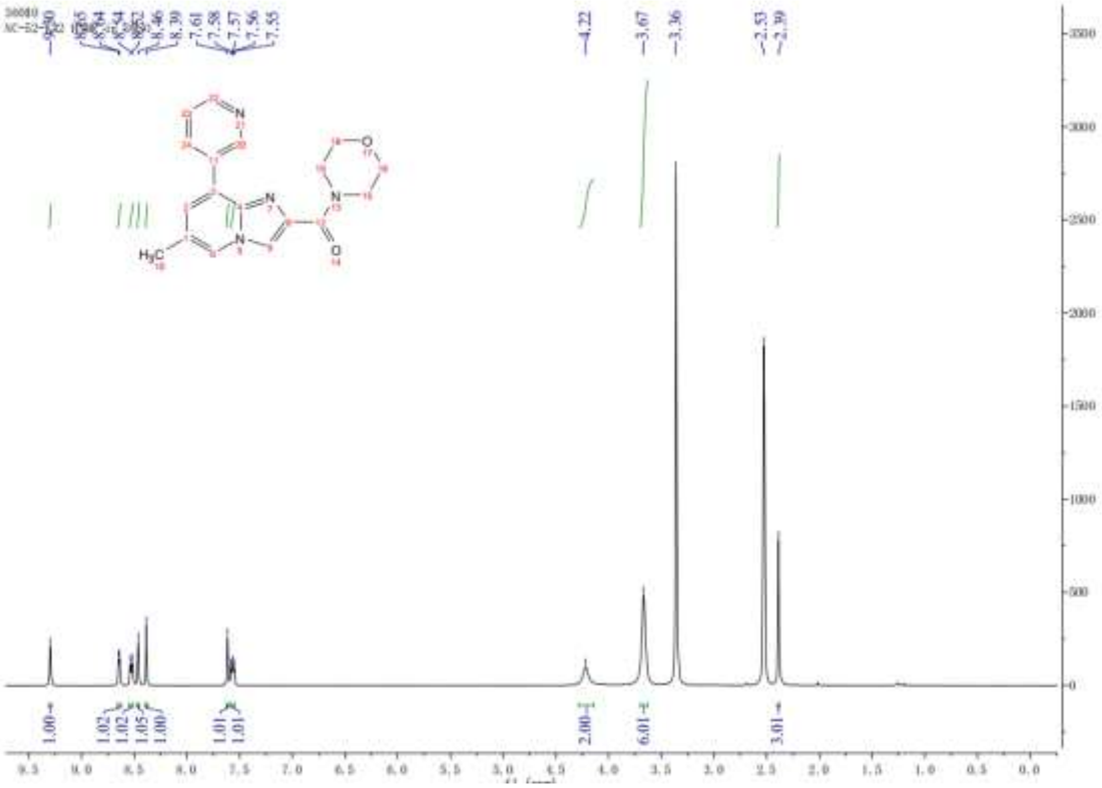

Figure S7. ^{1}H-NMR spectrum of compound **20.**

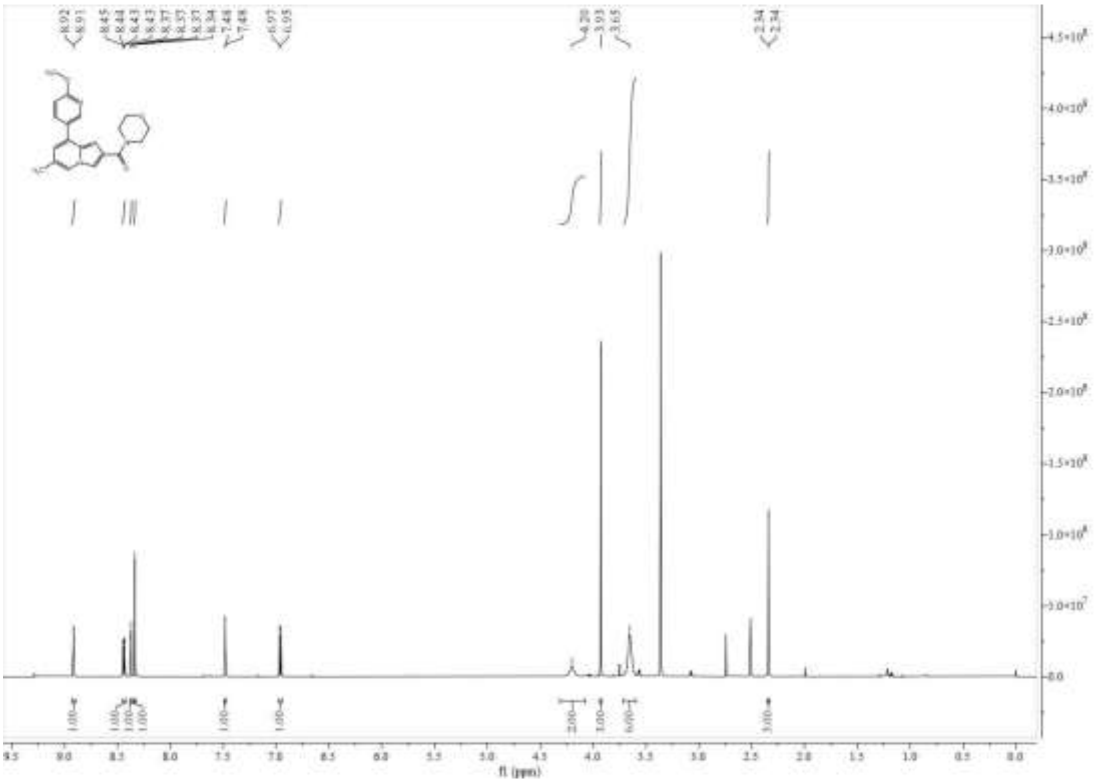

Figure S8. ^{1}H-NMR spectrum of compound **21.**

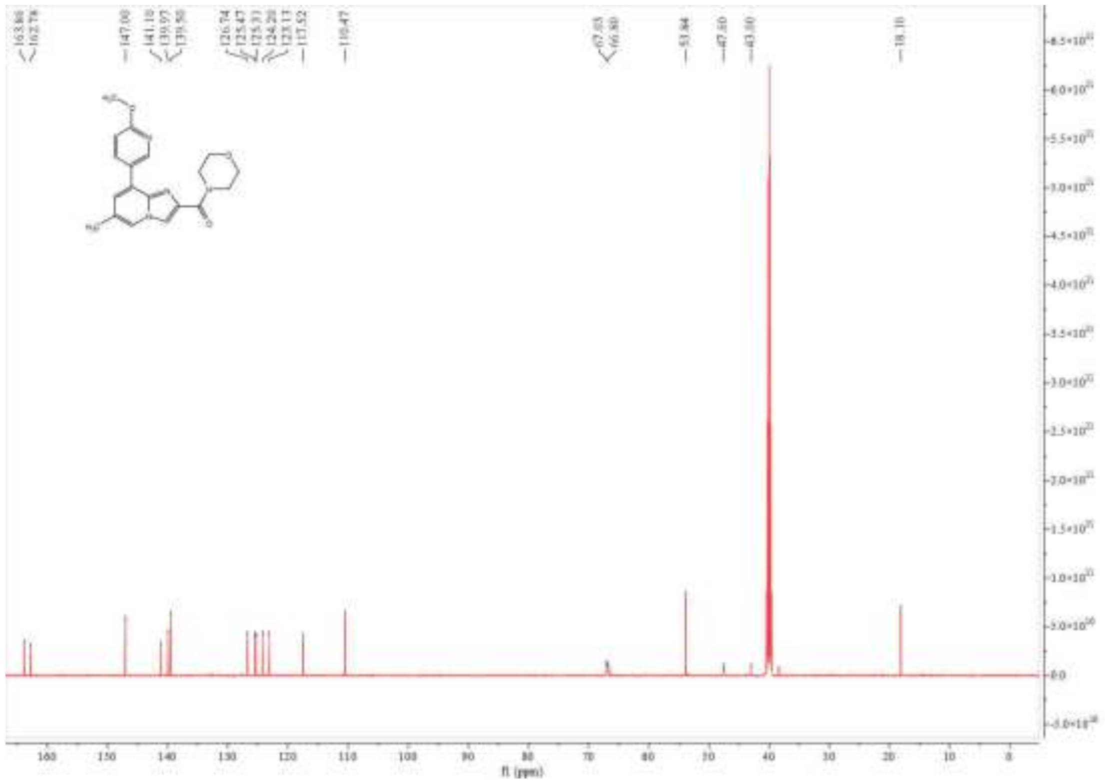

Figure S9. ^{13}C-NMR spectrum of compound **21.**

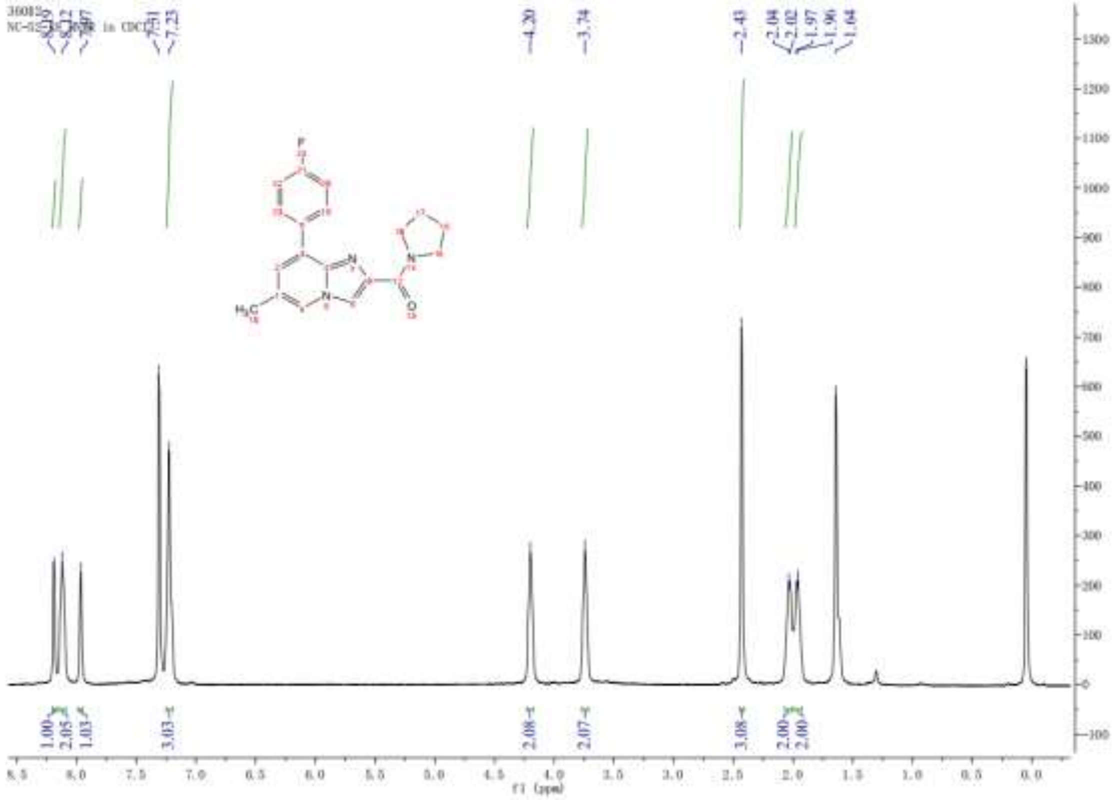

Figure S10. ^{1}H-NMR spectrum of compound **22.**

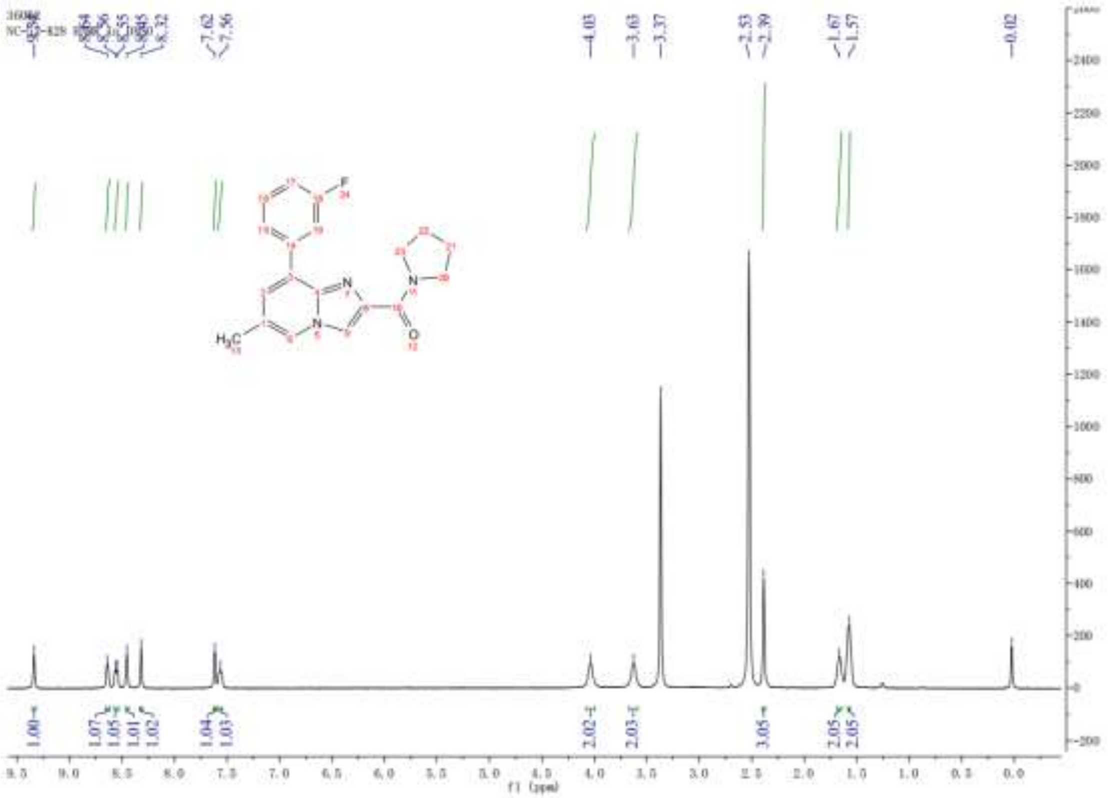

Figure S11. ^{1}H-NMR spectrum of compound **23.**

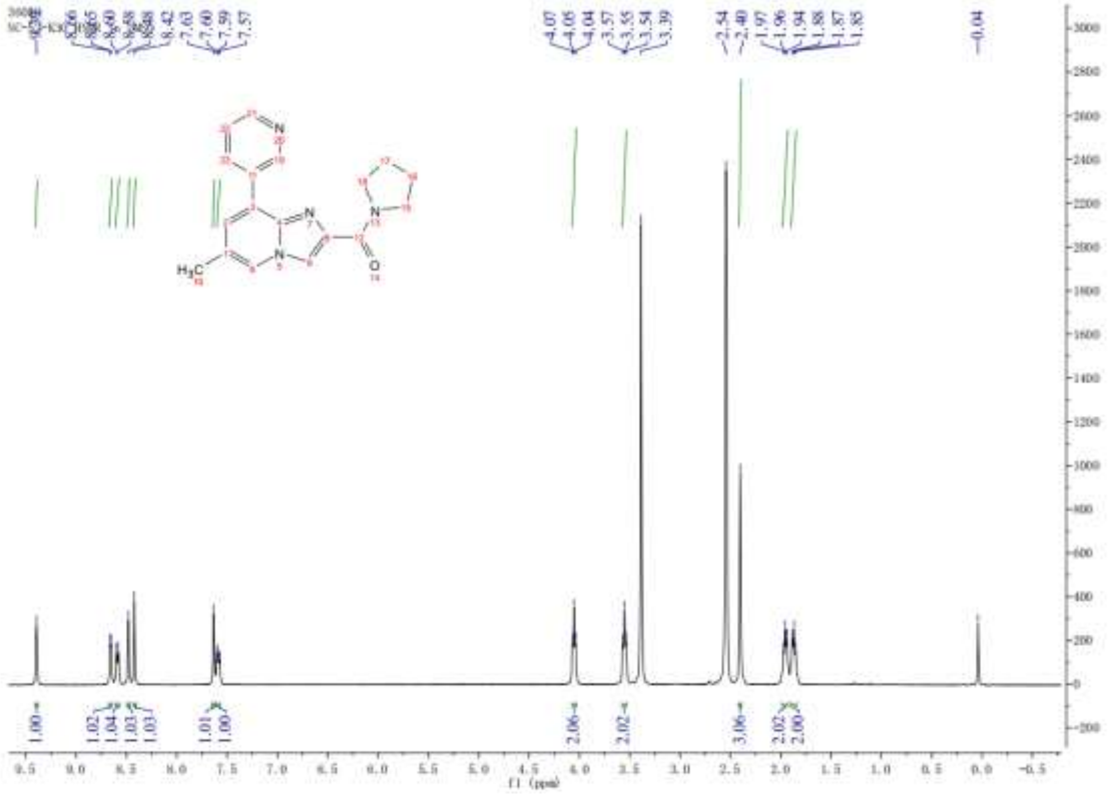

Figure S12. ^{1}H-NMR spectrum of compound **24.**

Information Classification: General

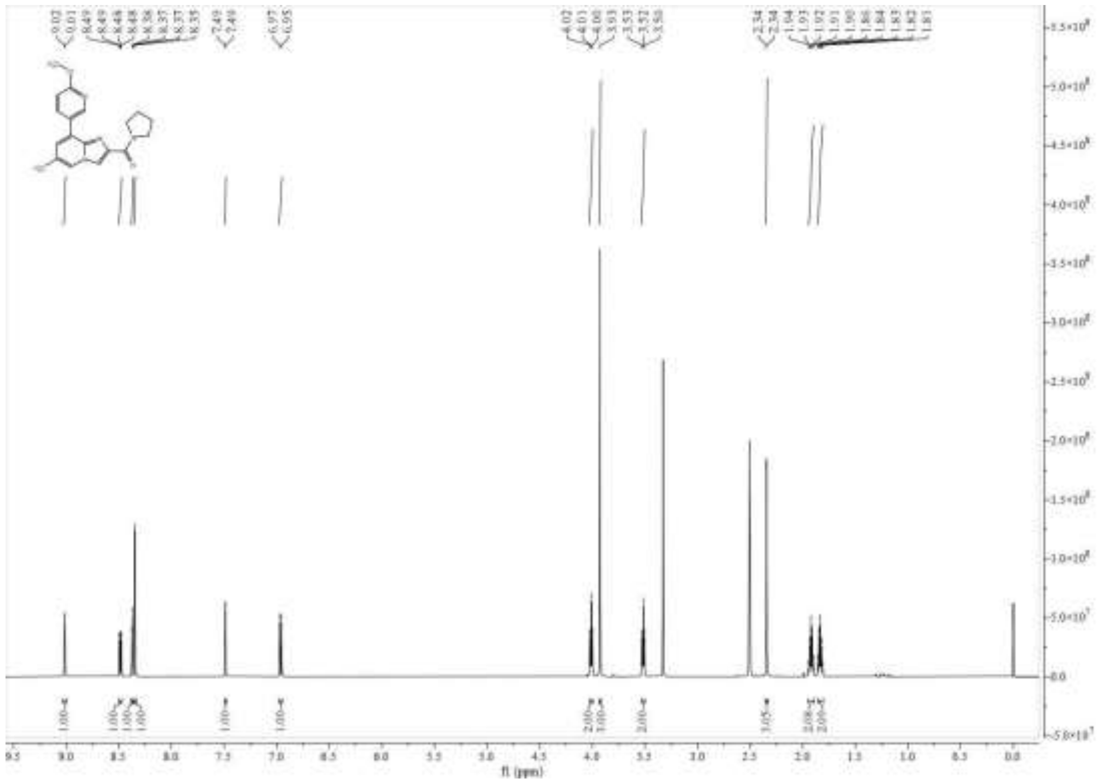

Figure S13. ^{1}H-NMR spectrum of compound **25.**

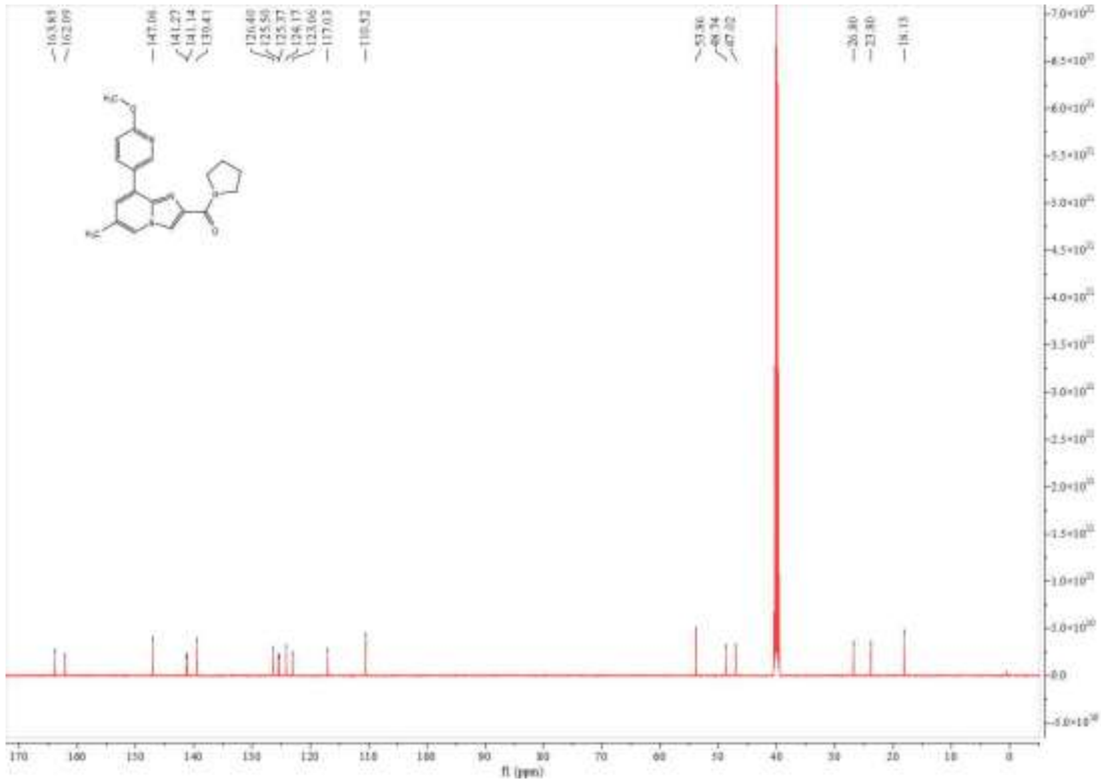

Figure S14. ^{13}C-NMR spectrum of compound **25.**

Information Classification: General

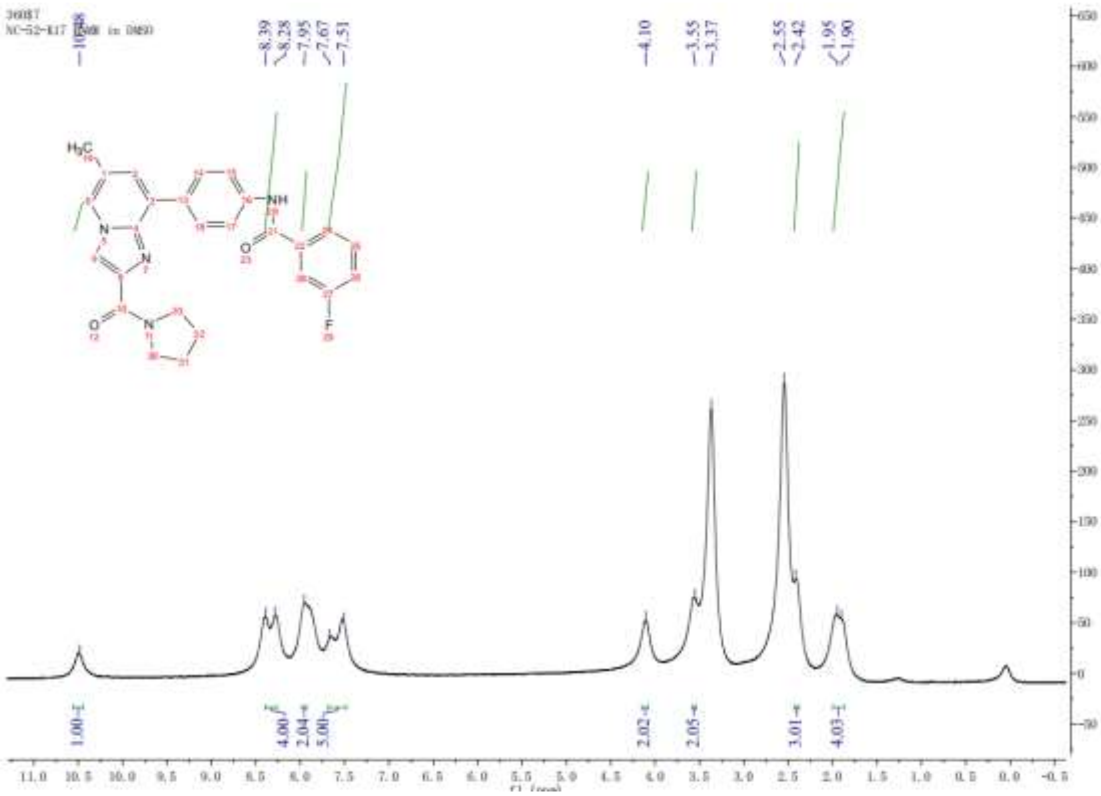

Figure S15. ^{1}H-NMR spectrum of compound **26.**

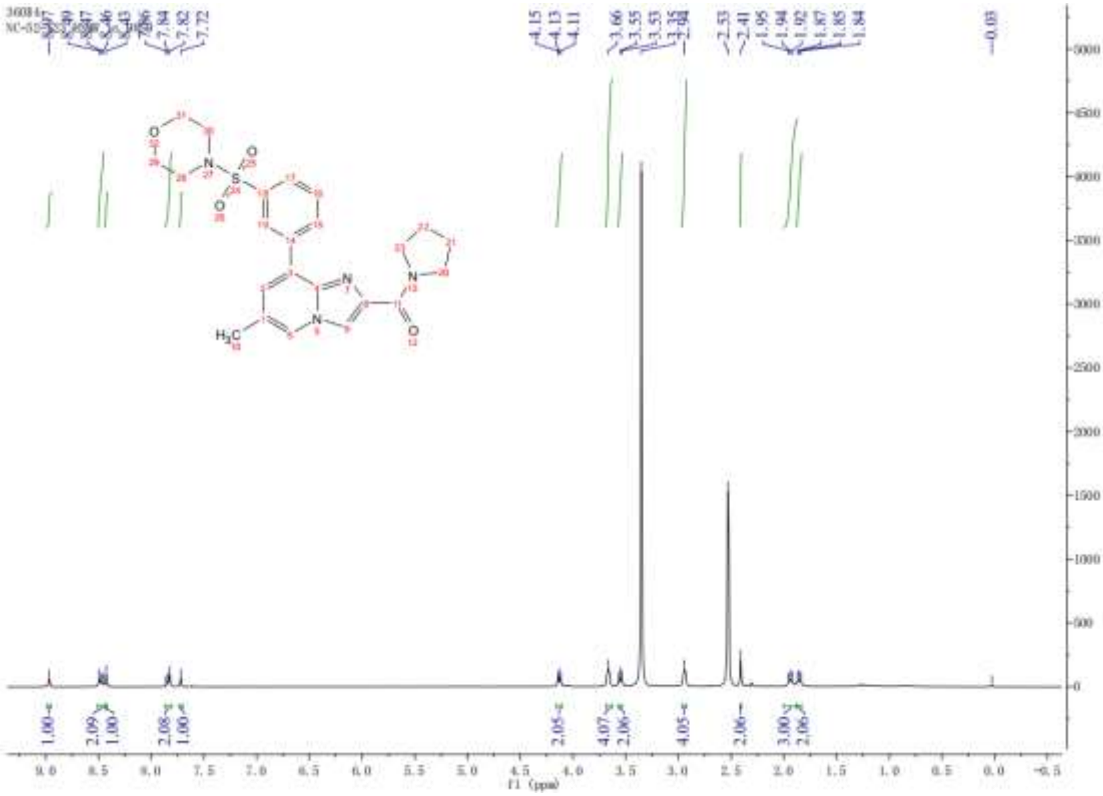

Figure S16. ^{1}H-NMR spectrum of compound **27.**

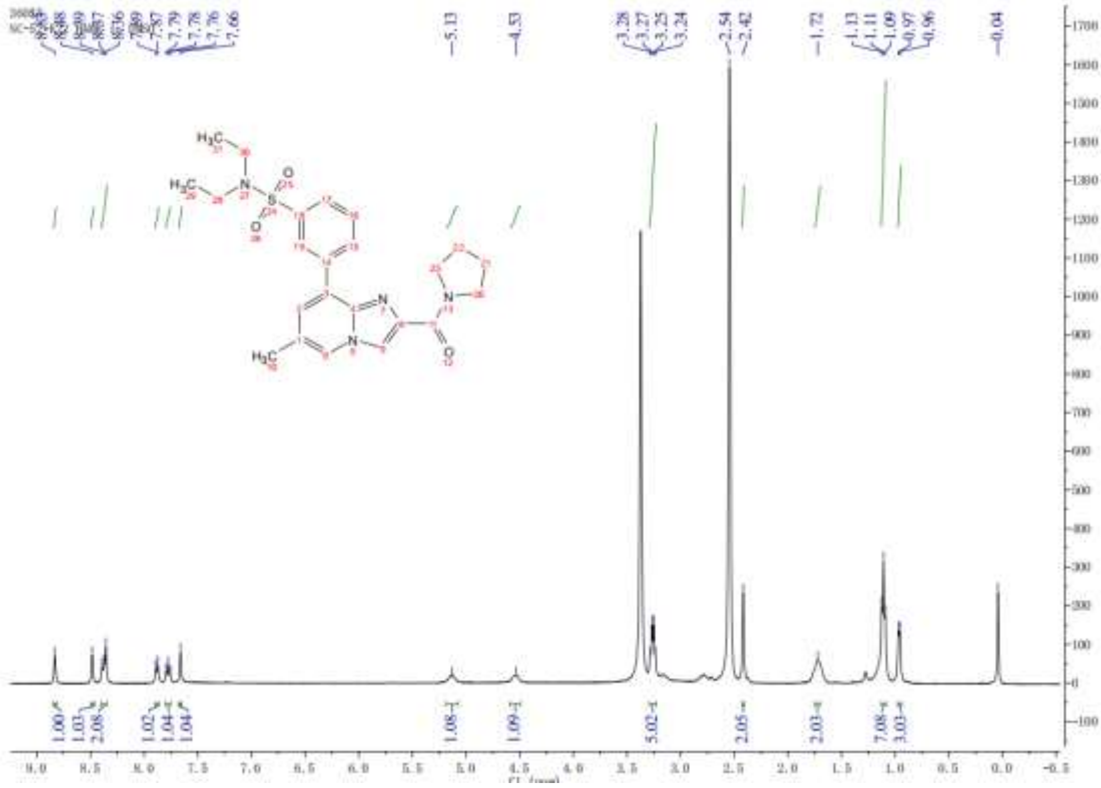

Figure S17. ^{1}H-NMR spectrum of compound **28.**

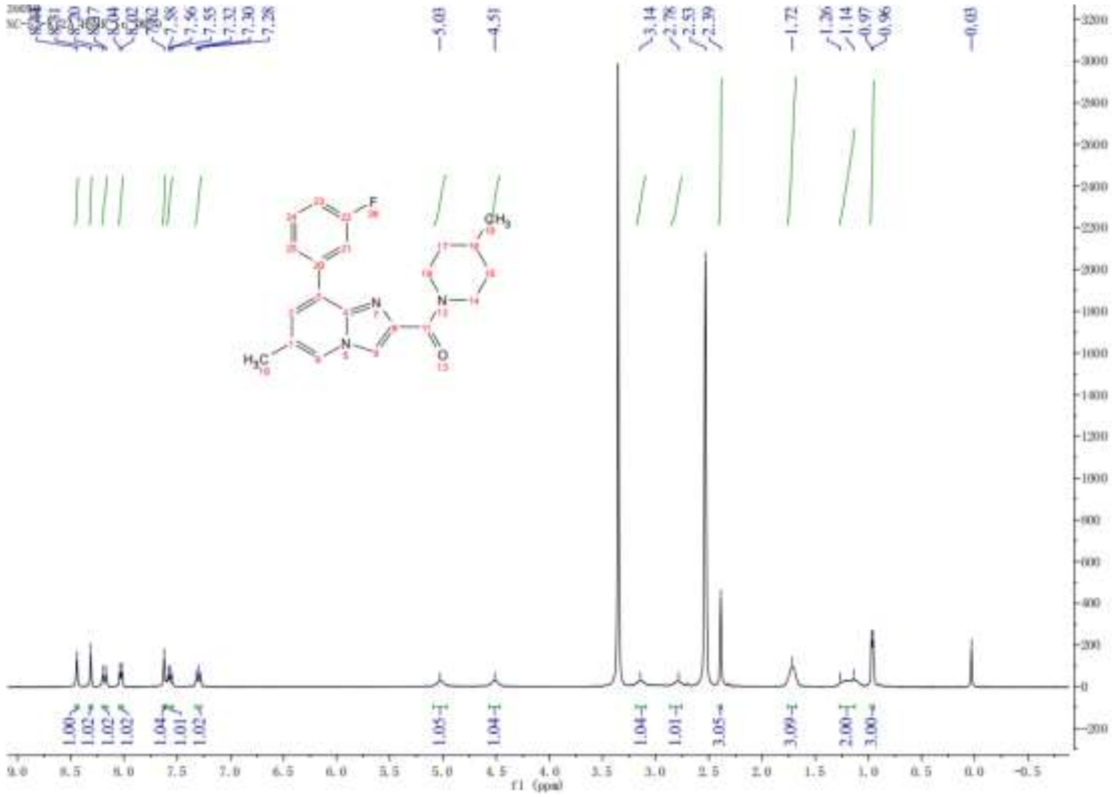

Figure S18. ^{1}H-NMR spectrum of compound **29.**

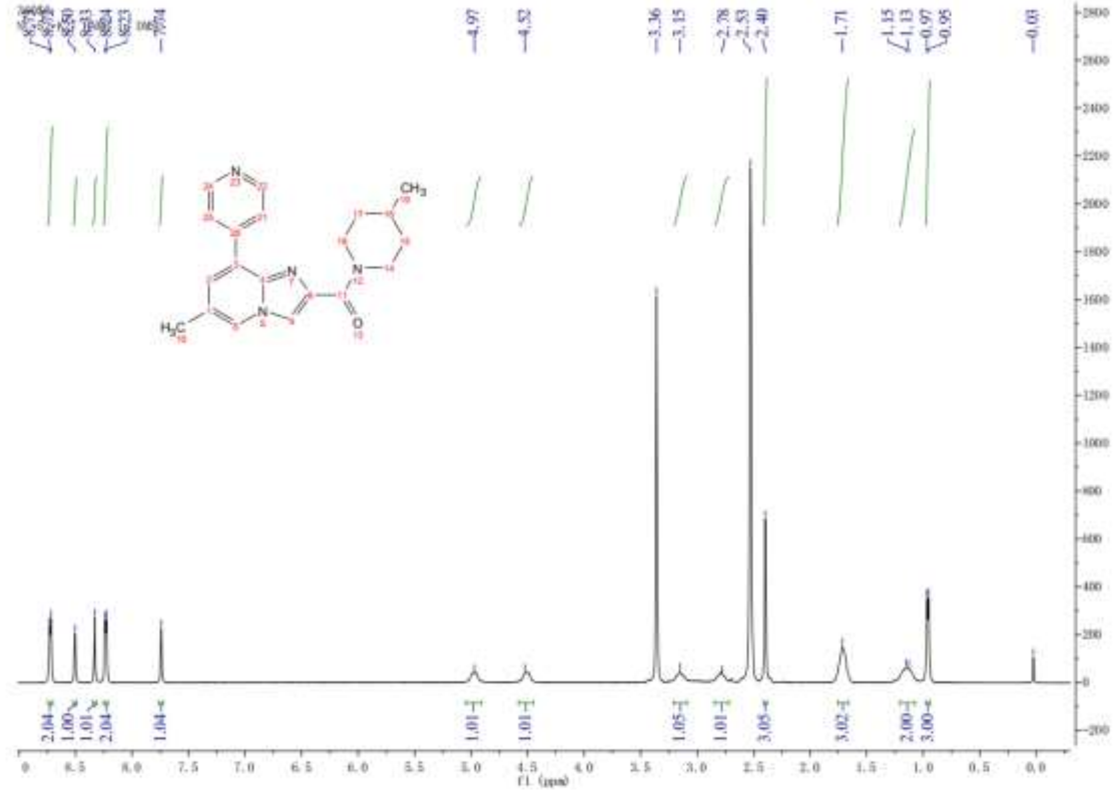

Figure S19. ^{1}H-NMR spectrum of compound **30.**

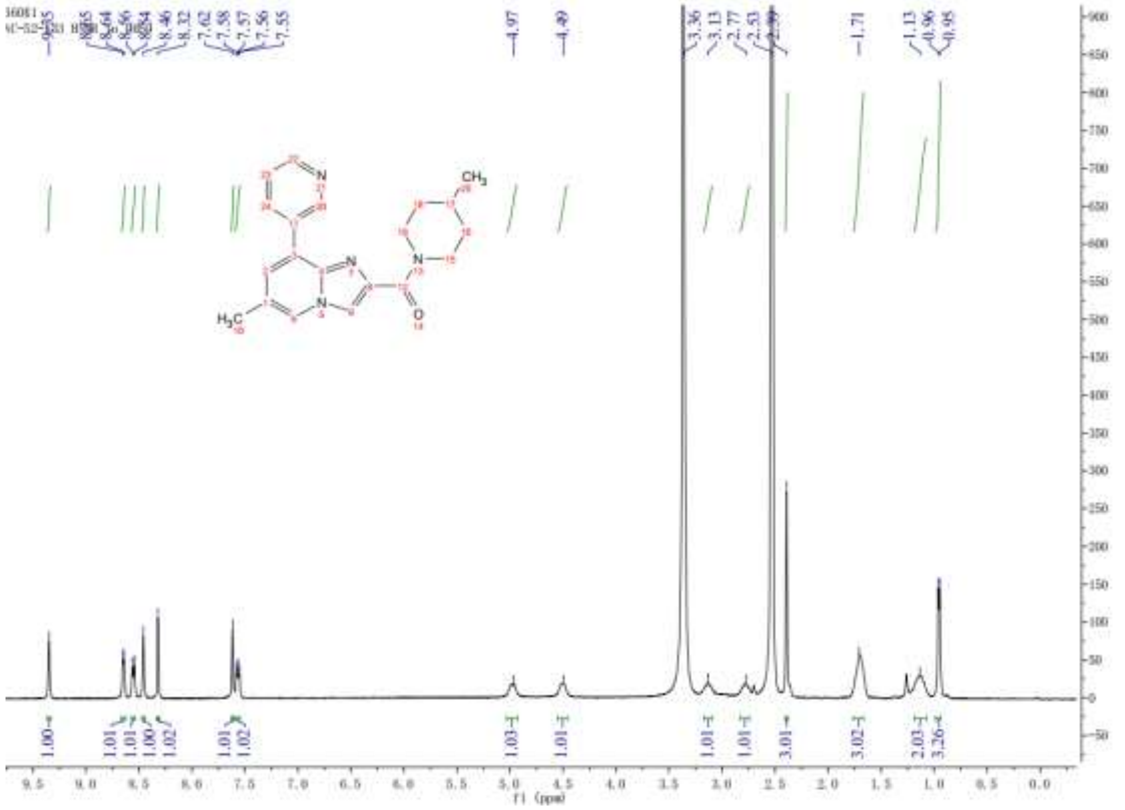

Figure S20. ^{1}H-NMR spectrum of compound **31.**

Information Classification: General

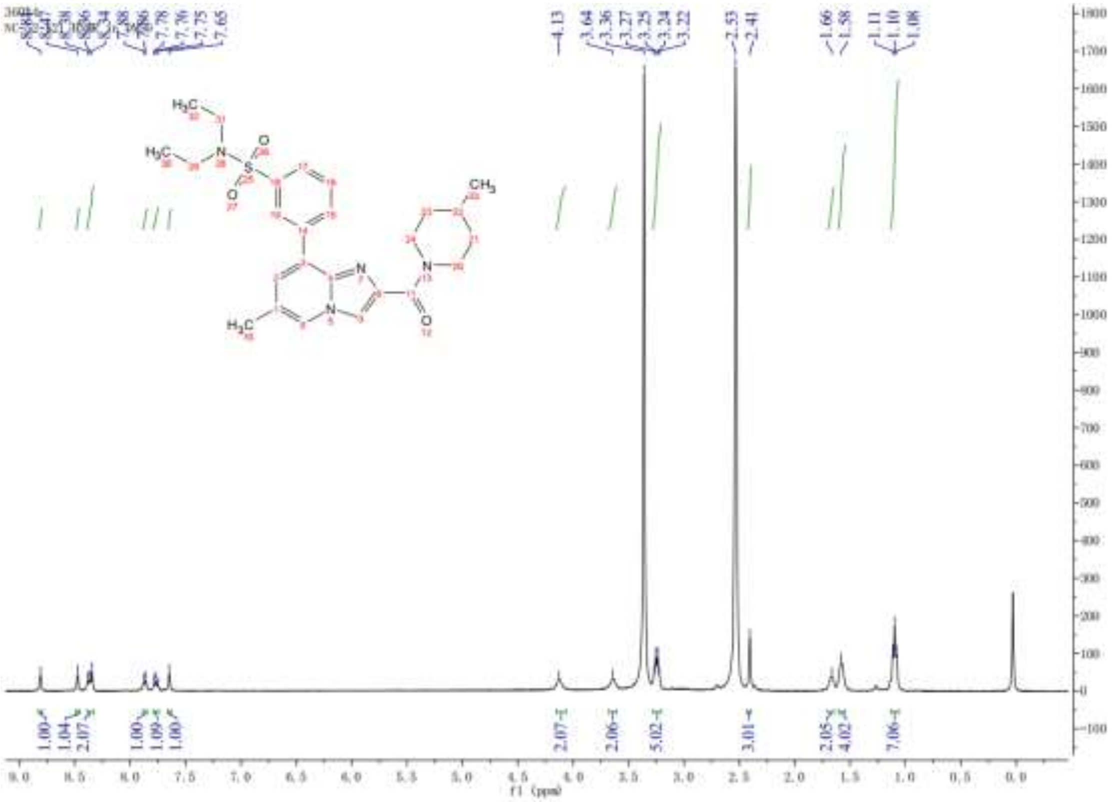

Figure S21. ^{1}H-NMR spectrum of compound **32.**

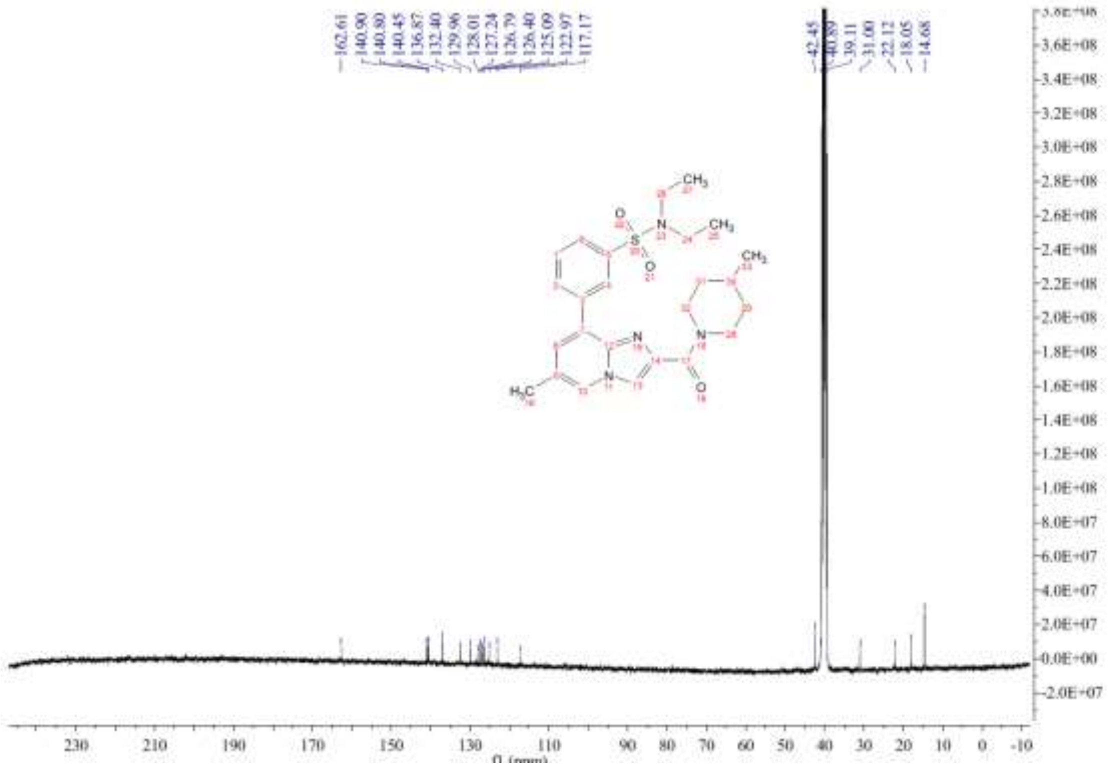

Figure S22. ^{13}C-NMR spectrum of compound **32.**

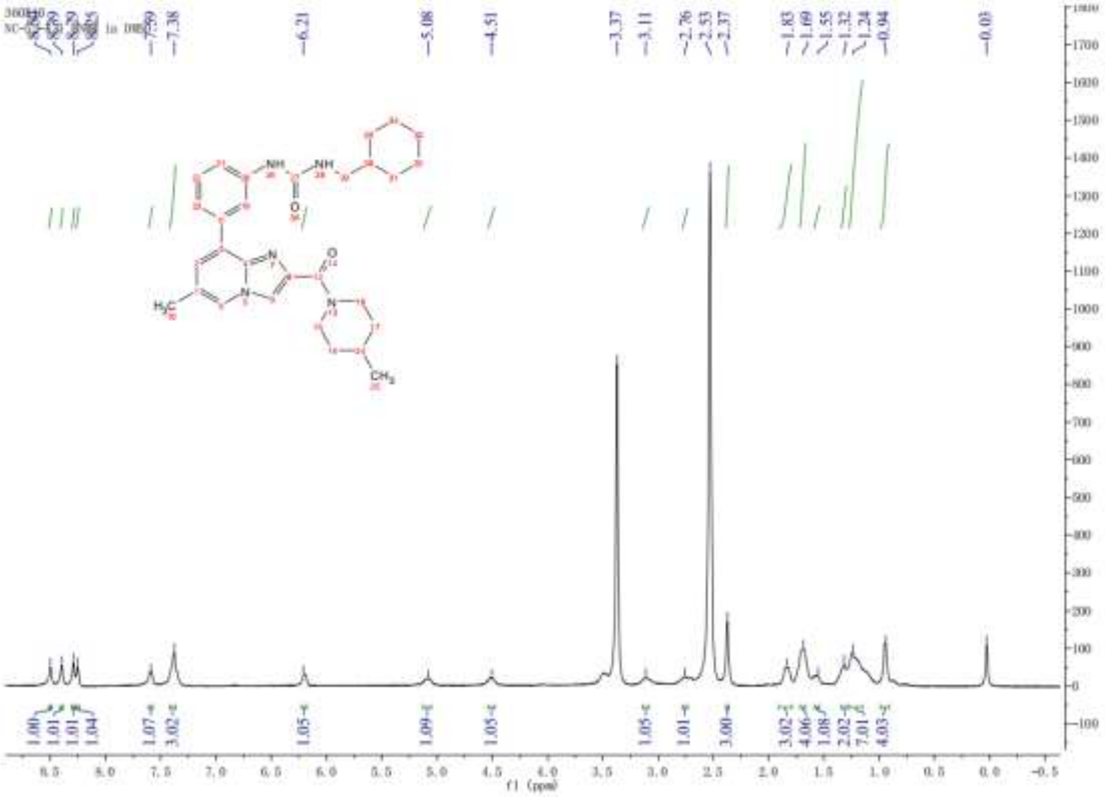

Figure S23. ^{1}H-NMR spectrum of compound **33.**

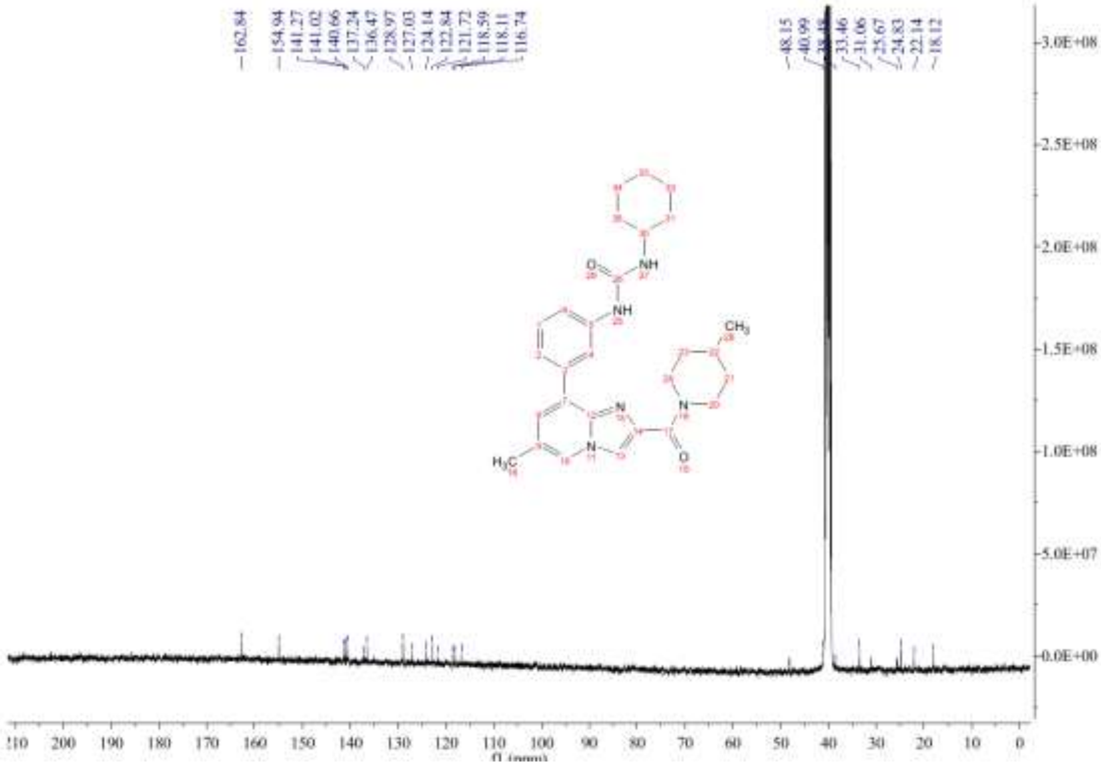

Figure S24. ^{13}C-NMR spectrum of compound **33.**

Information Classification: General

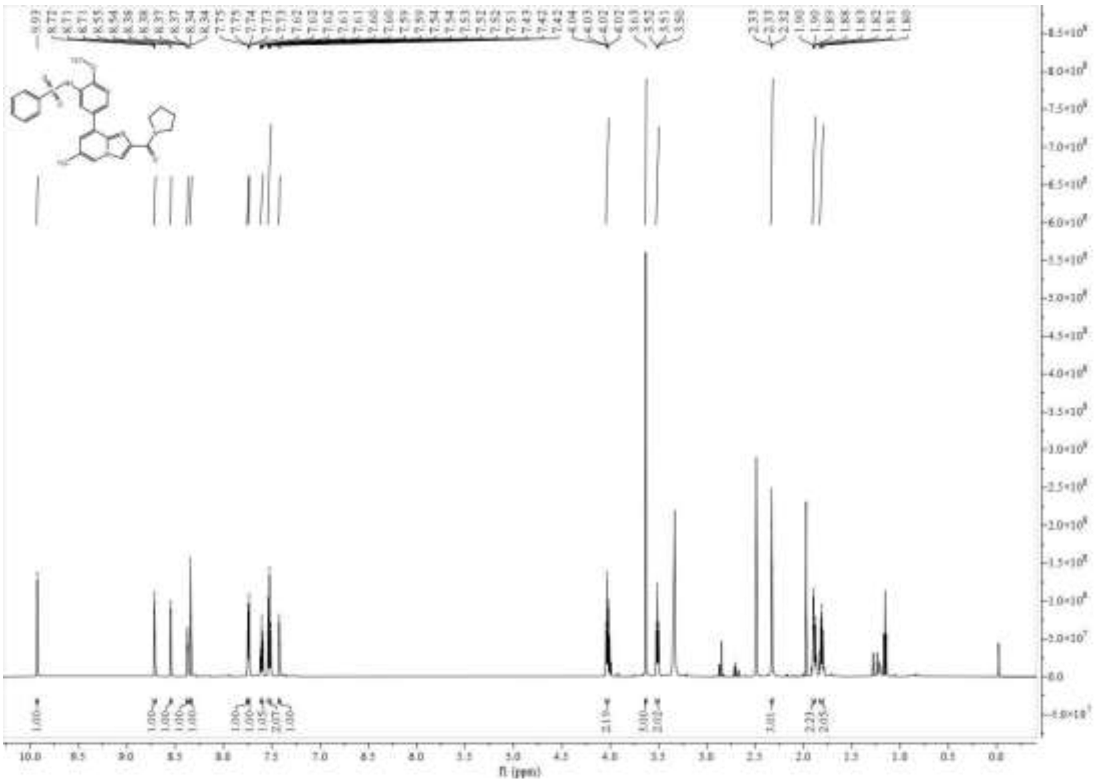

Figure S25. ^{1}H-NMR spectrum of compound **34.**

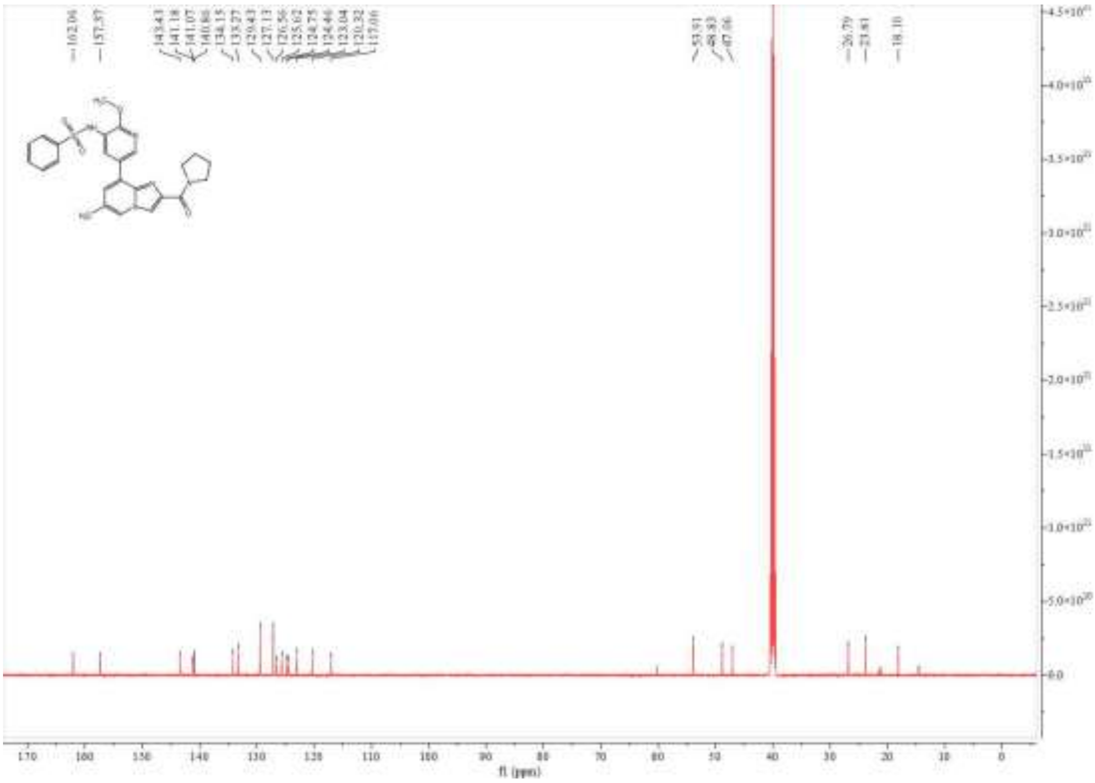

Figure S26. ^{13}C-NMR spectrum of compound **34.**

Information Classification: General

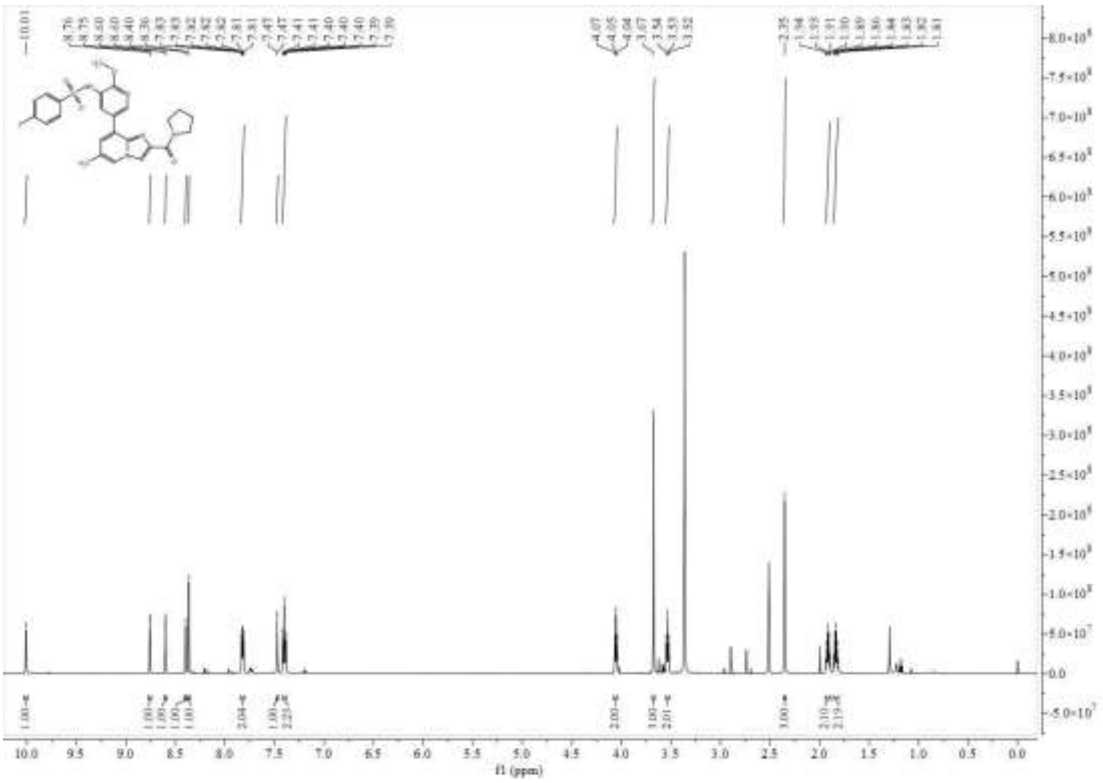

Figure S27. ^{1}H-NMR spectrum of compound **35.**

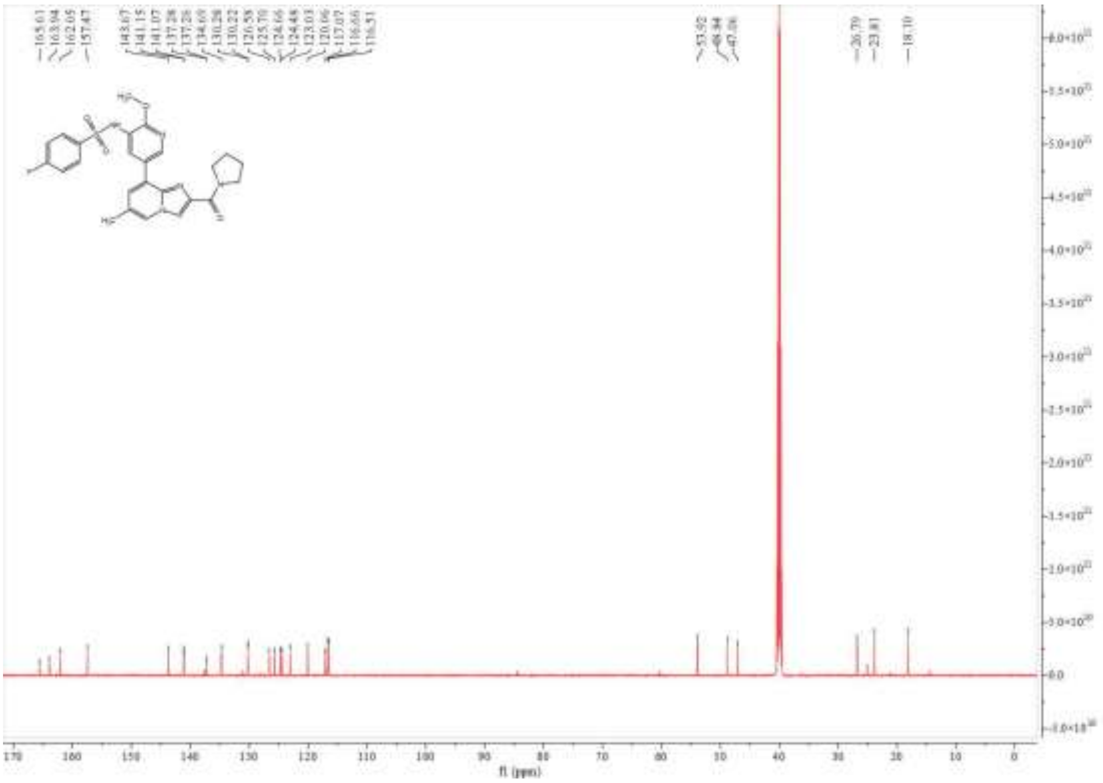

Figure S28. ^{13}C-NMR spectrum of compound **35.**

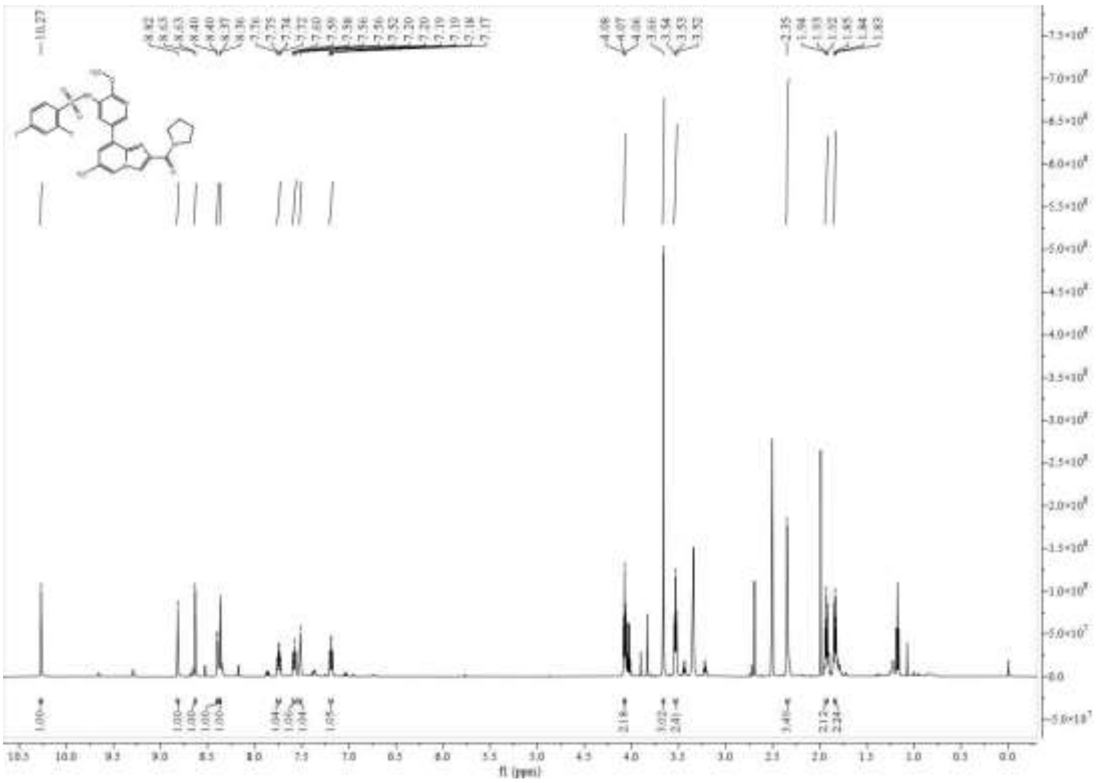

Figure S29. ^{1}H-NMR spectrum of compound **36.**

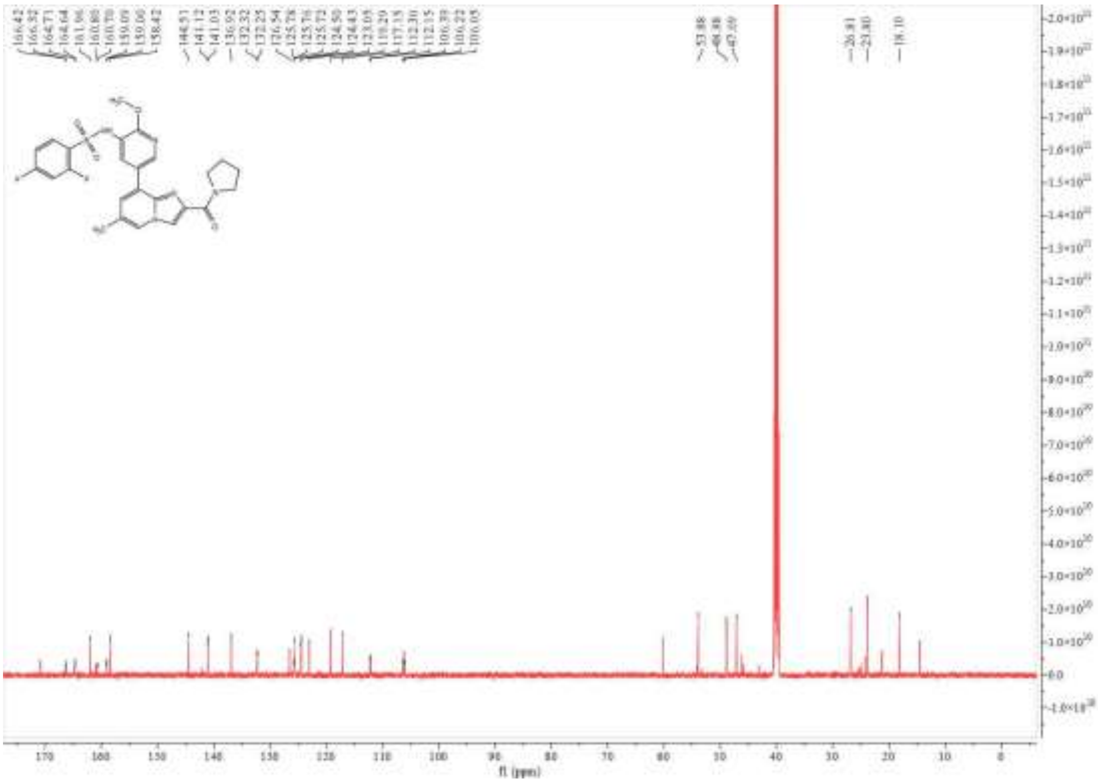

Figure S30. ^{13}C-NMR spectrum of compound **36.**

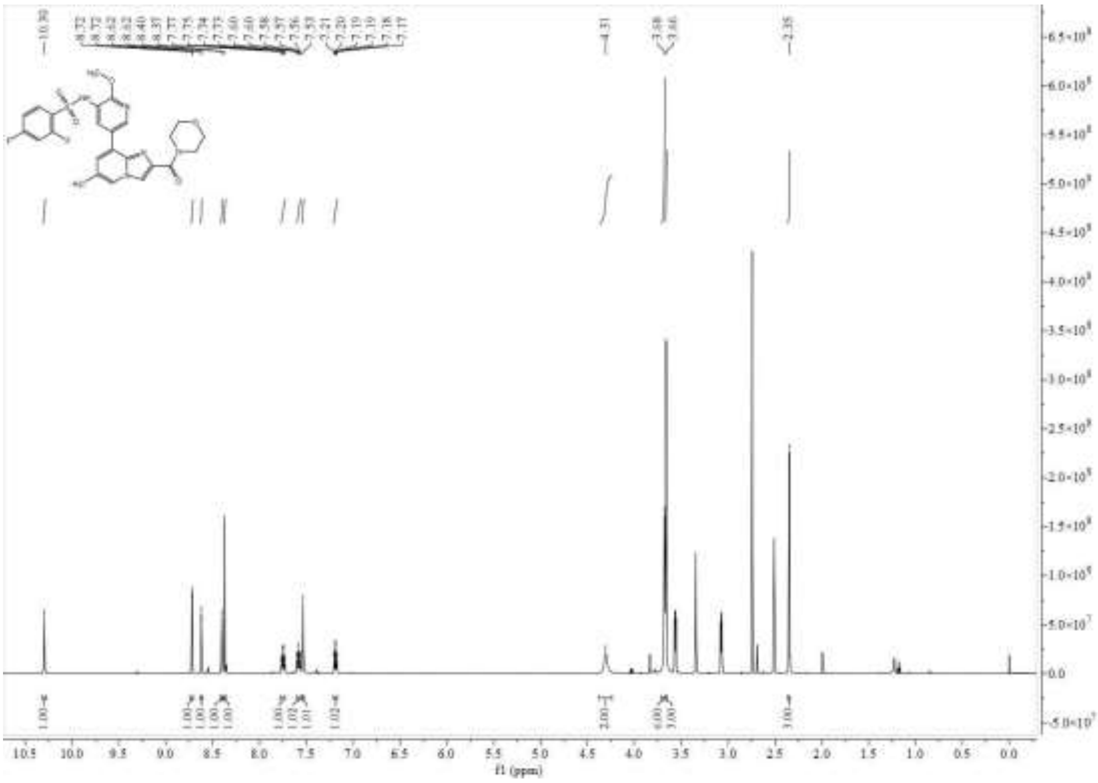

Figure S31. ^{1}H-NMR spectrum of compound **37.**

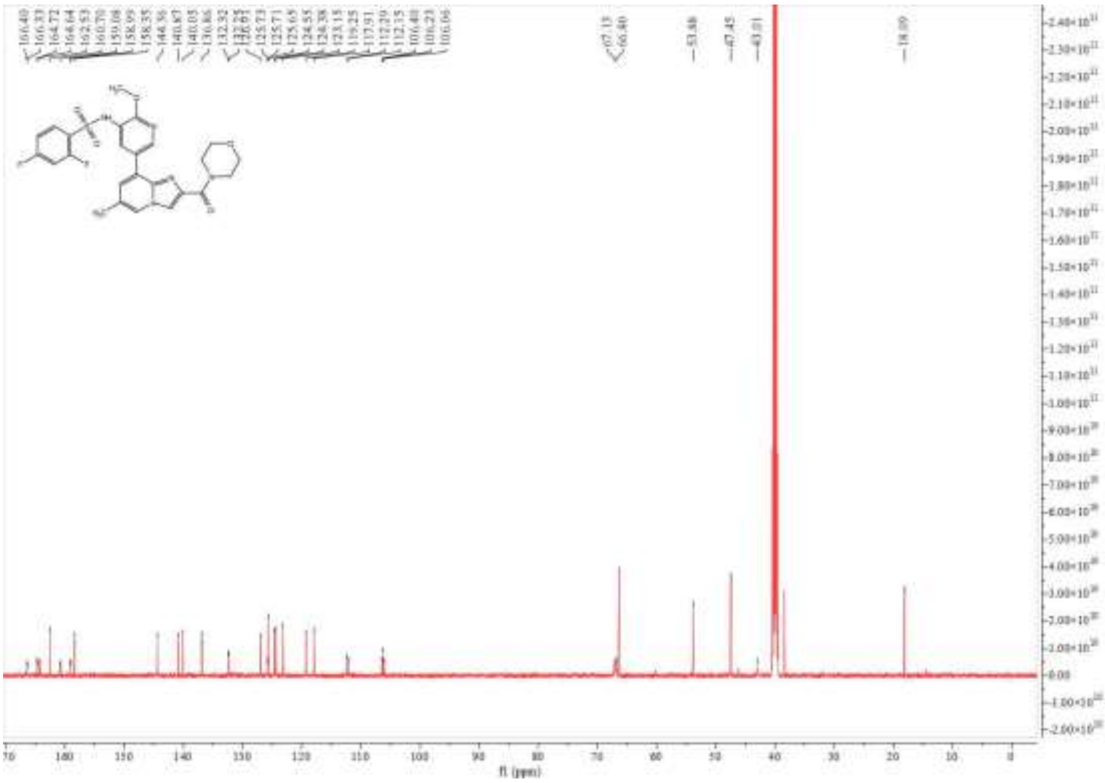

Figure S32. ^{13}C-NMR spectrum of compound **37.**

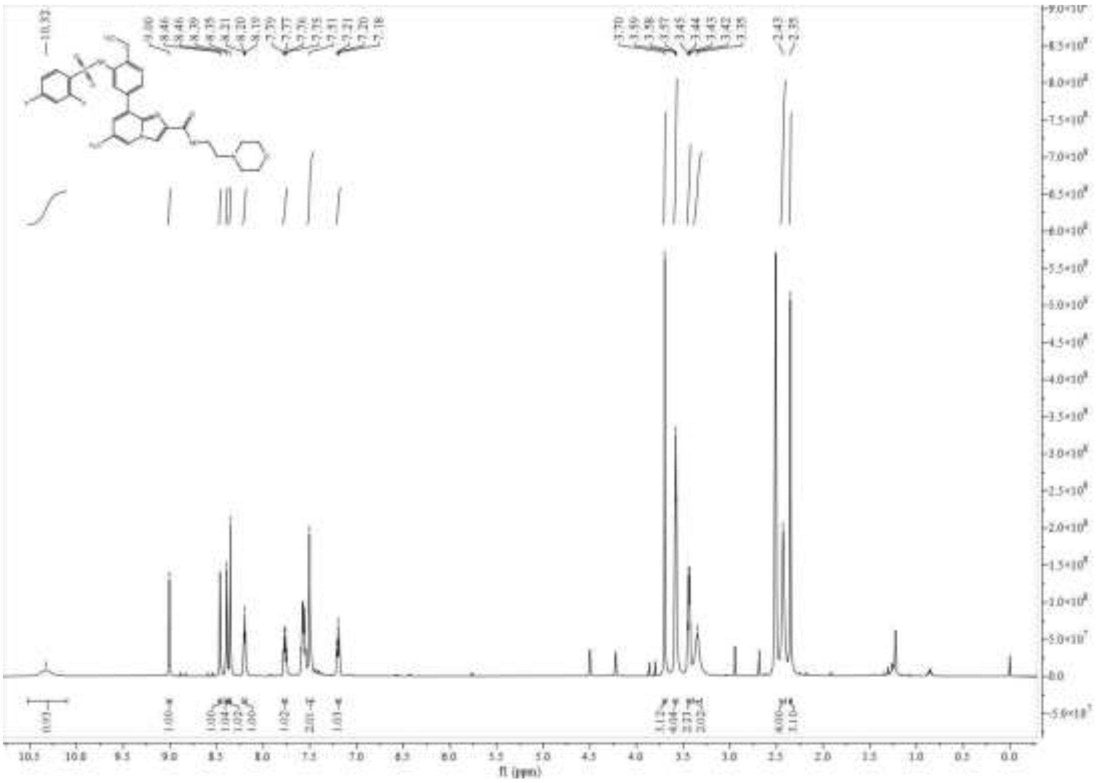

Figure S33. ^{1}H-NMR spectrum of compound **38.**

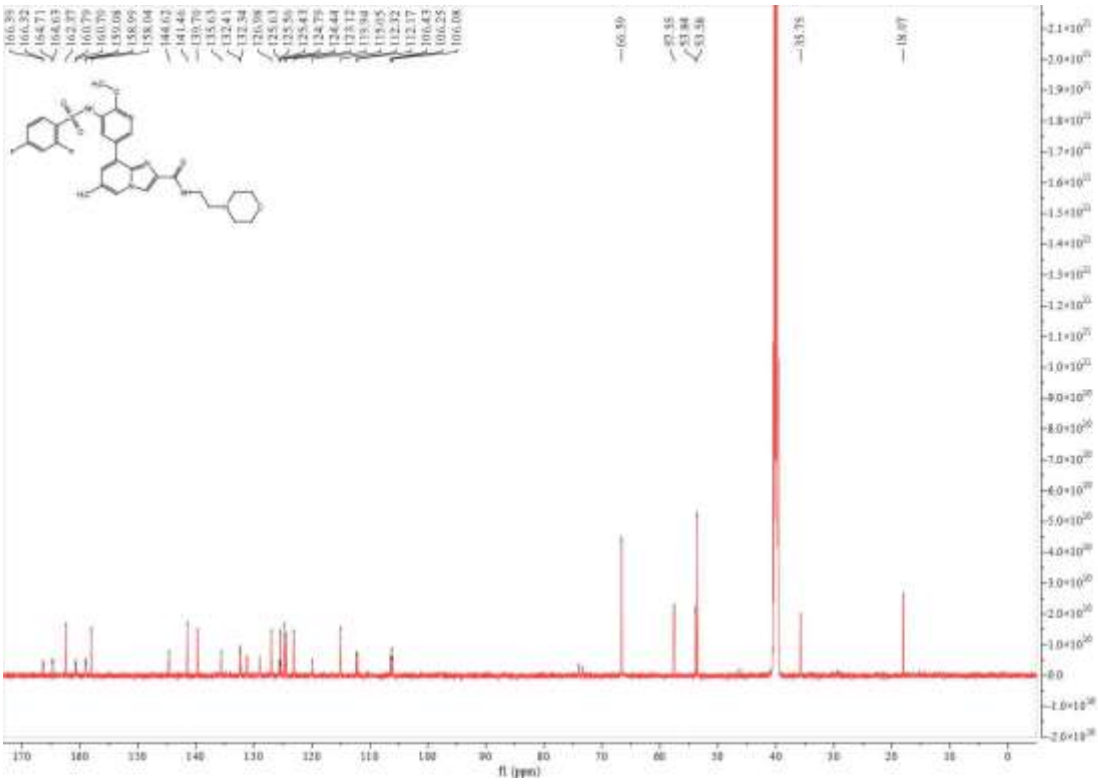

Figure S34. ^{13}C-NMR spectrum of compound **38.**

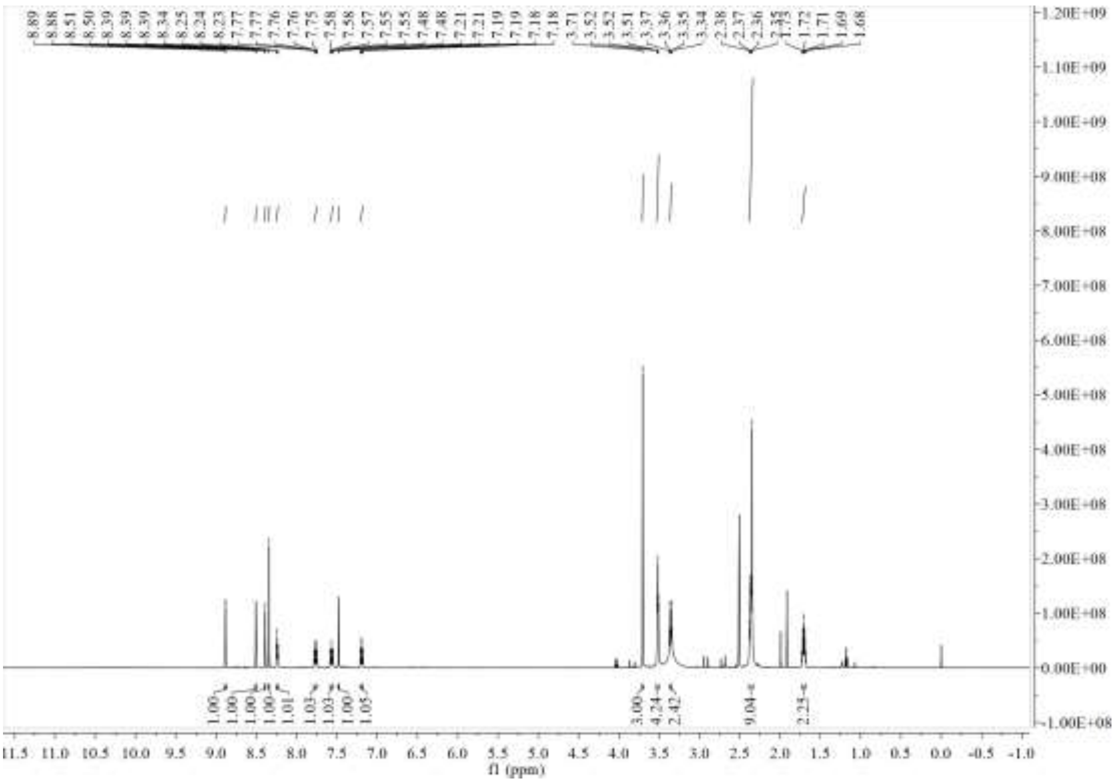

Figure S35. ^{1}H-NMR spectrum of compound **39.**

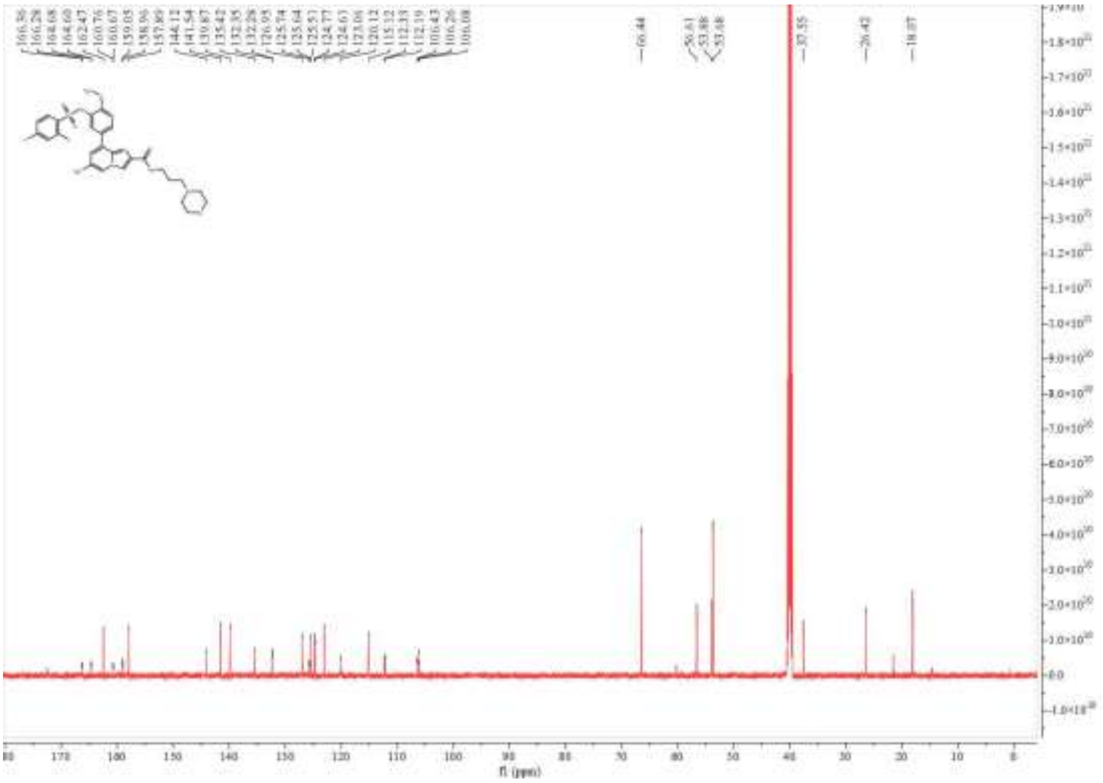

Figure S36. ^{13}C-NMR spectrum of compound **39.**

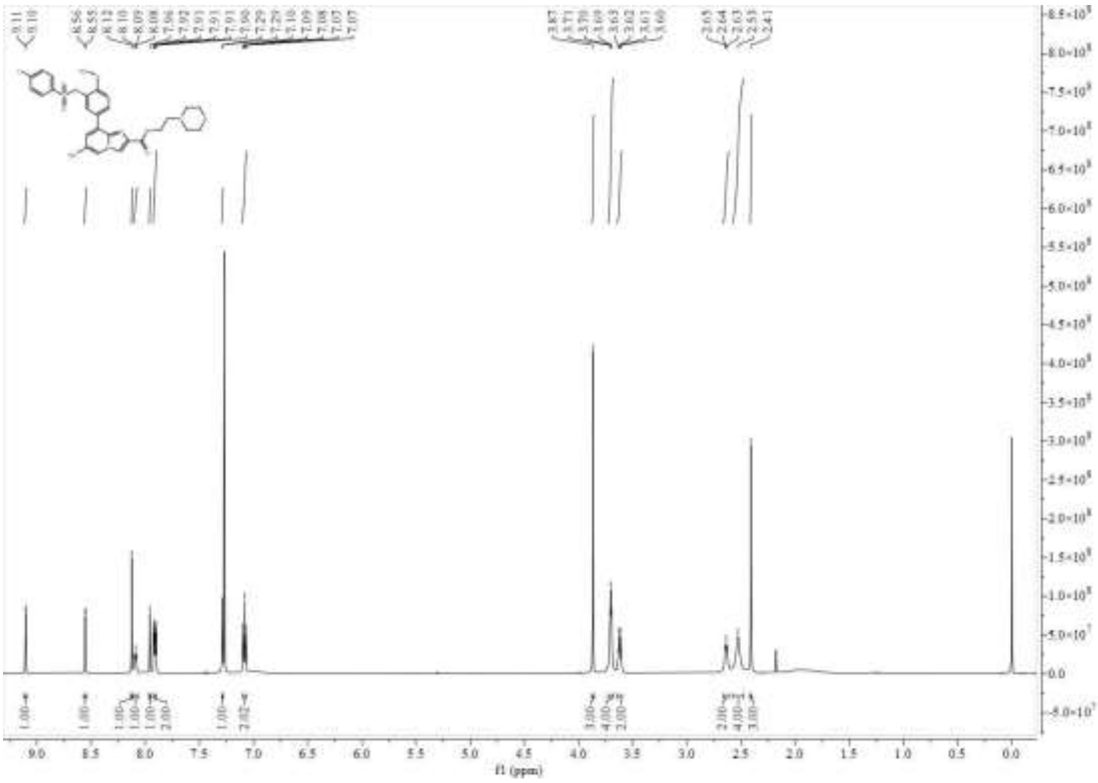

Figure S37. ^{1}H-NMR spectrum of compound **40.**

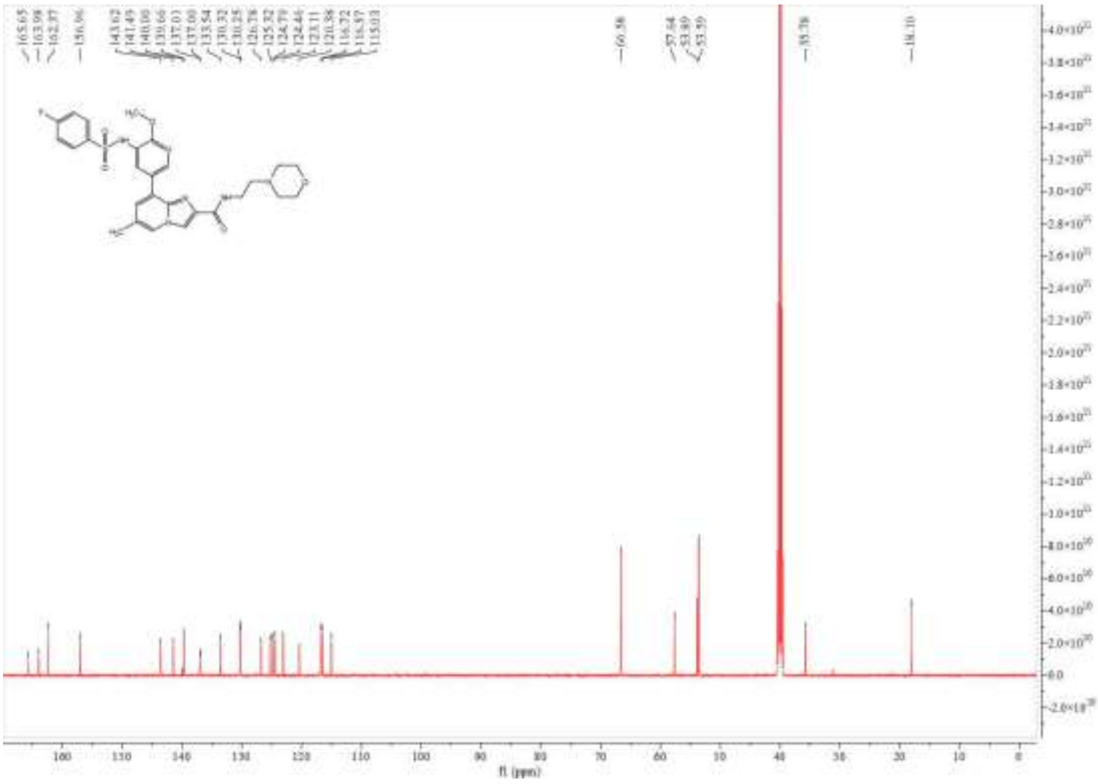

Figure S38. ^{13}C-NMR spectrum of compound **40.**

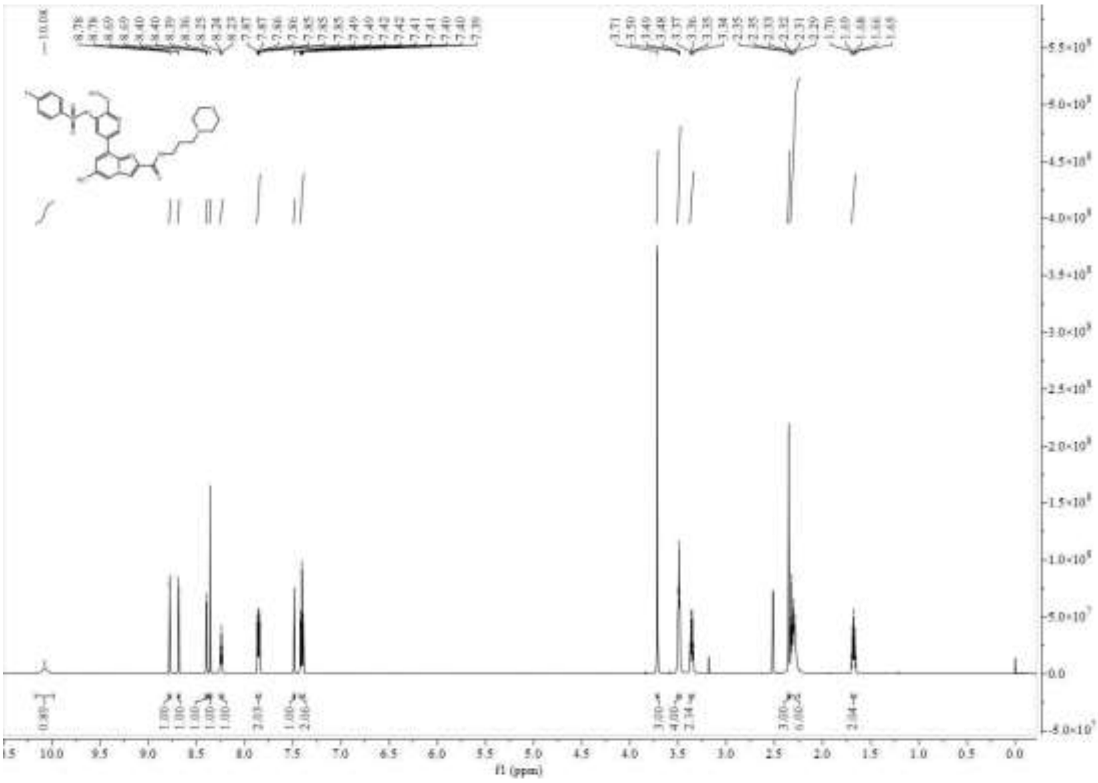

Figure S39. ^{1}H-NMR spectrum of compound **41.**

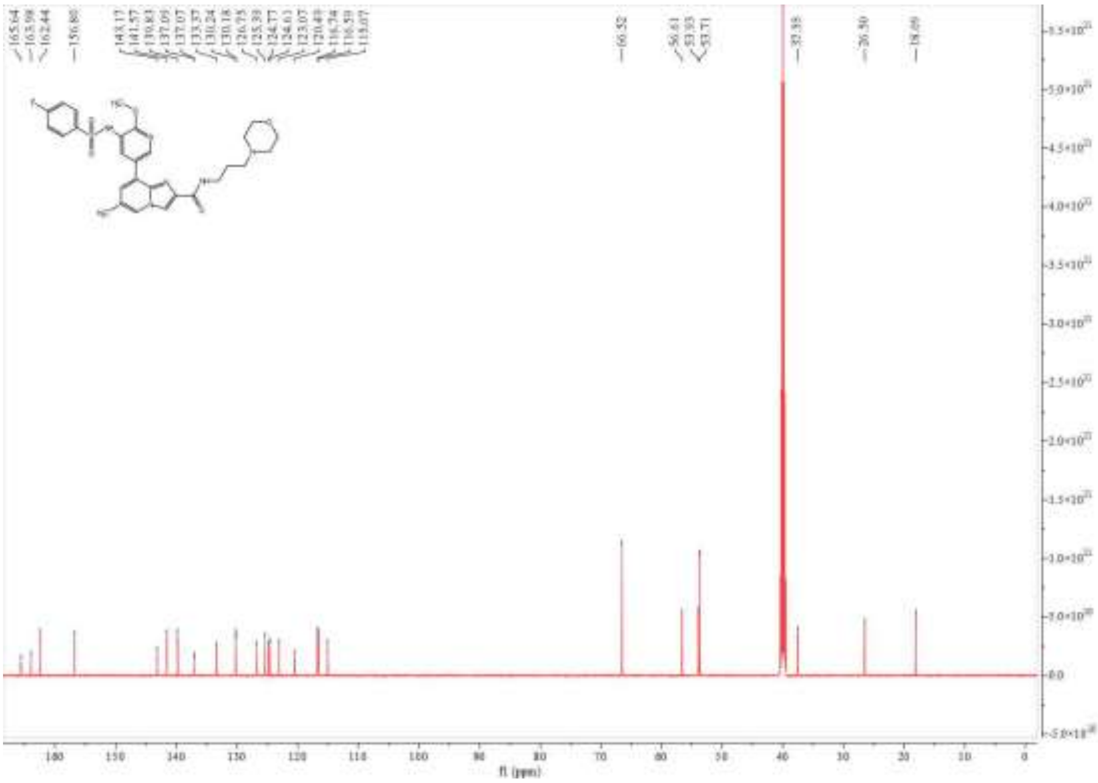

Figure S40. ^{13}C-NMR spectrum of compound **41.**

Information Classification: General

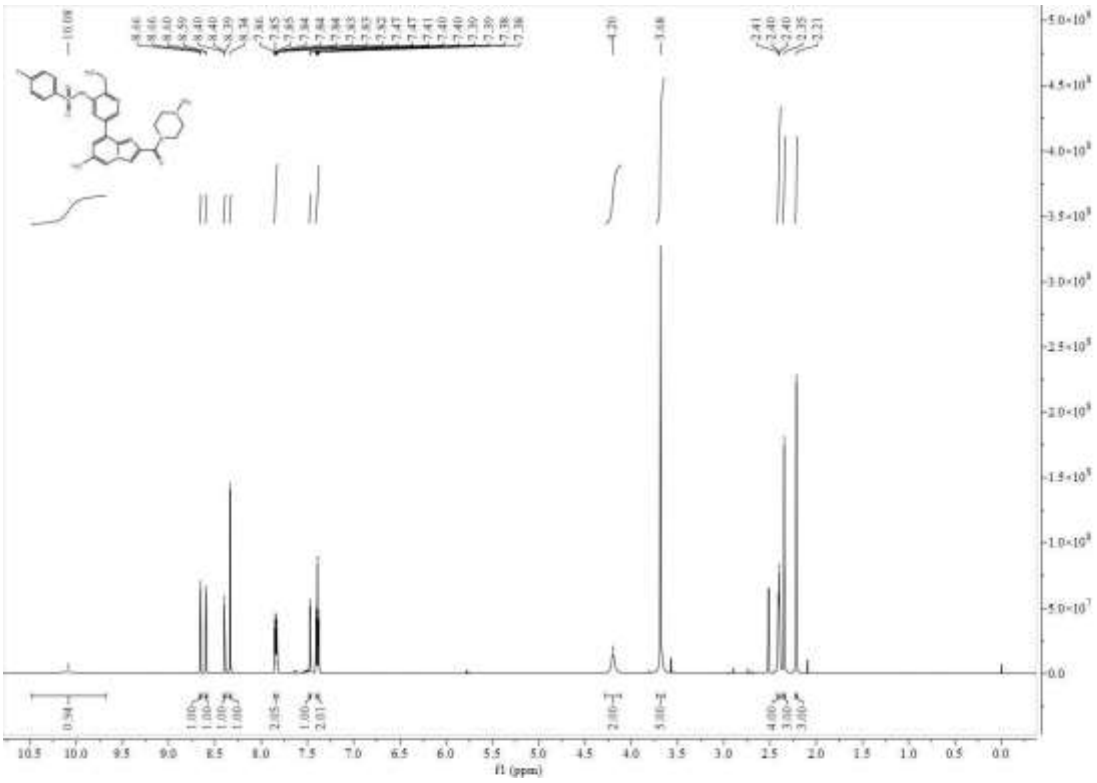

Figure S41. ^{1}H-NMR spectrum of compound **42.**

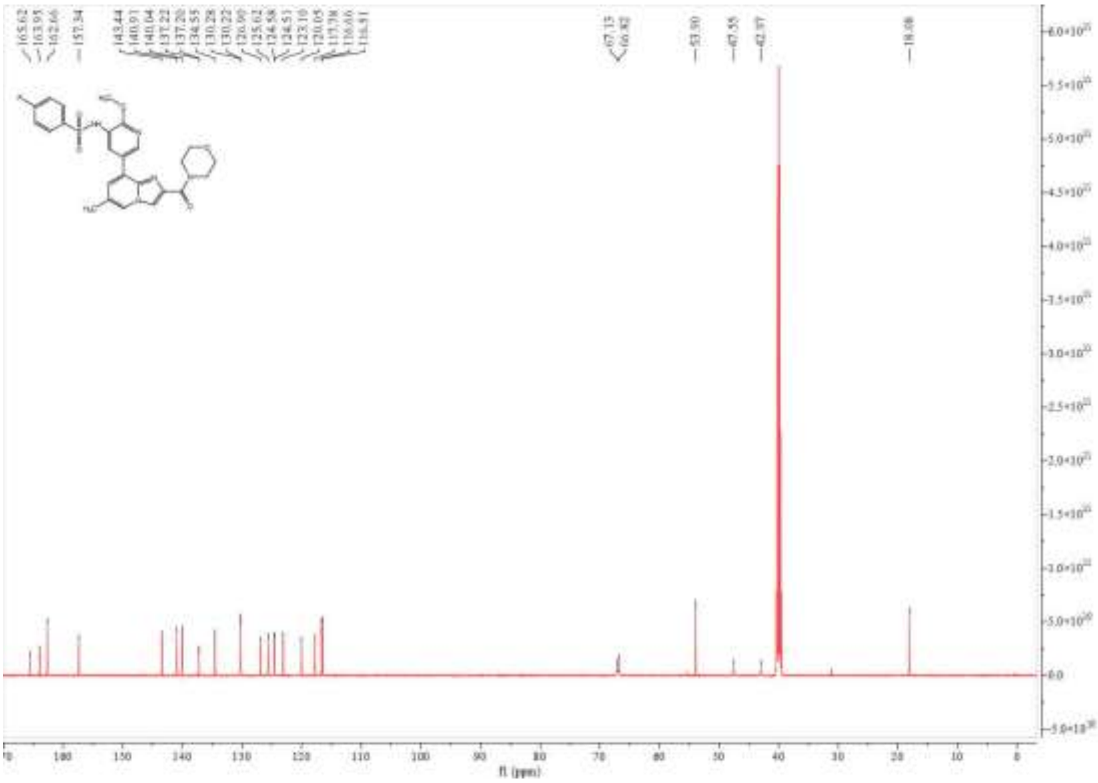

Figure S42. ^{13}C-NMR spectrum of compound **42.**

Information Classification: General

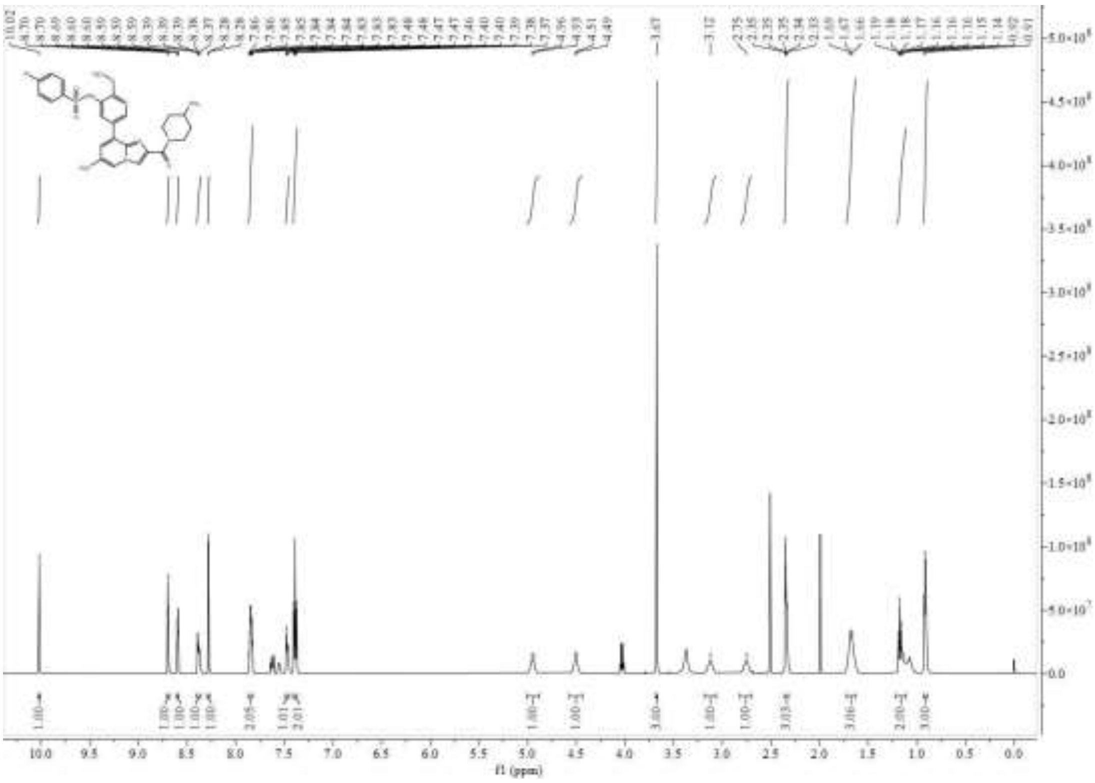

Figure S43. ^{1}H-NMR spectrum of compound **43.**

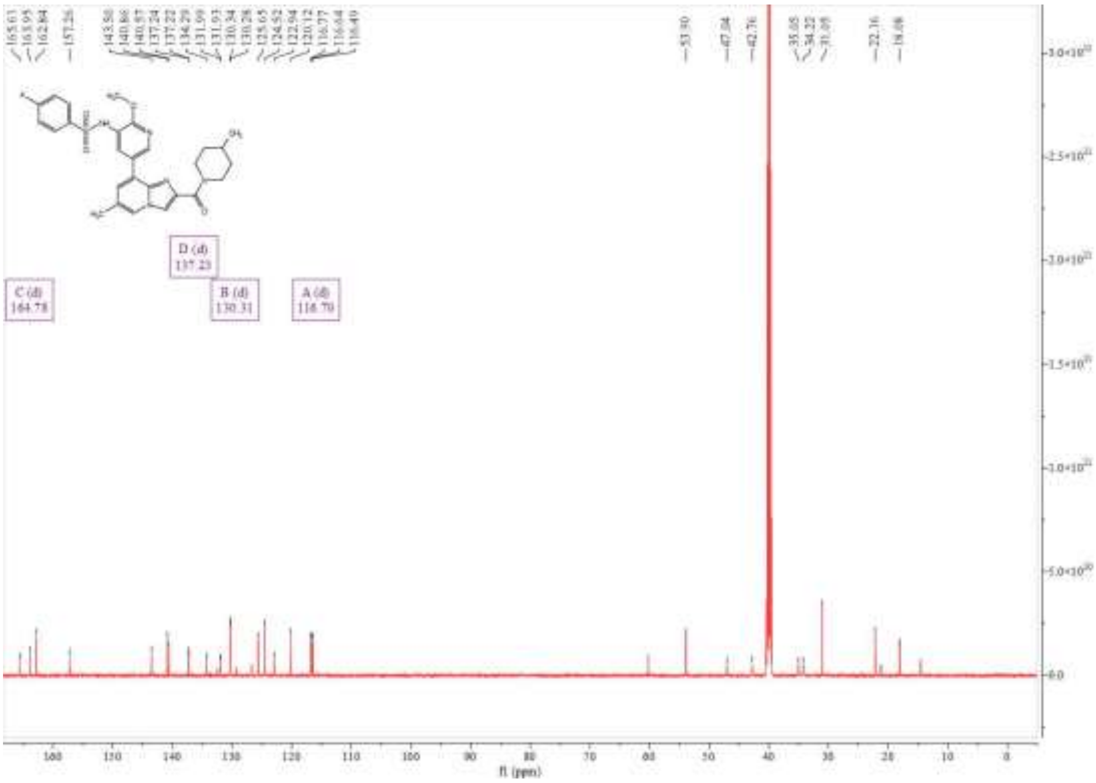

Figure S44. ^{13}C-NMR spectrum of compound **43.**

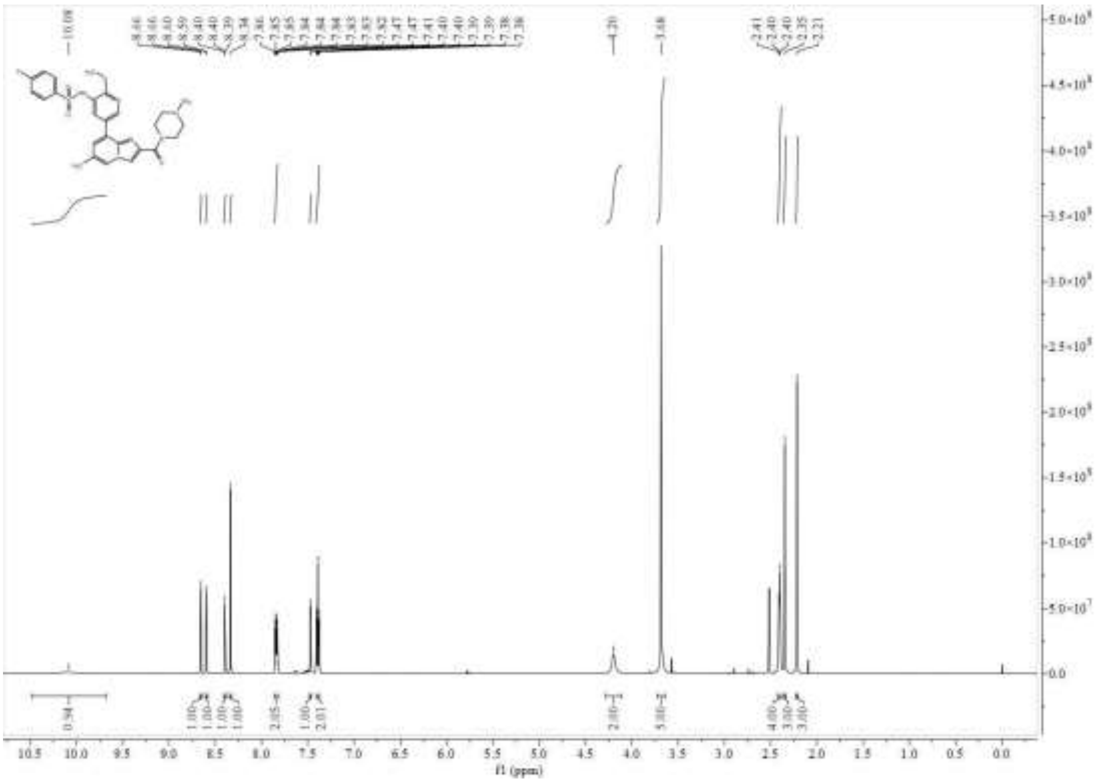

Figure S45. ^{1}H-NMR spectrum of compound **44.**

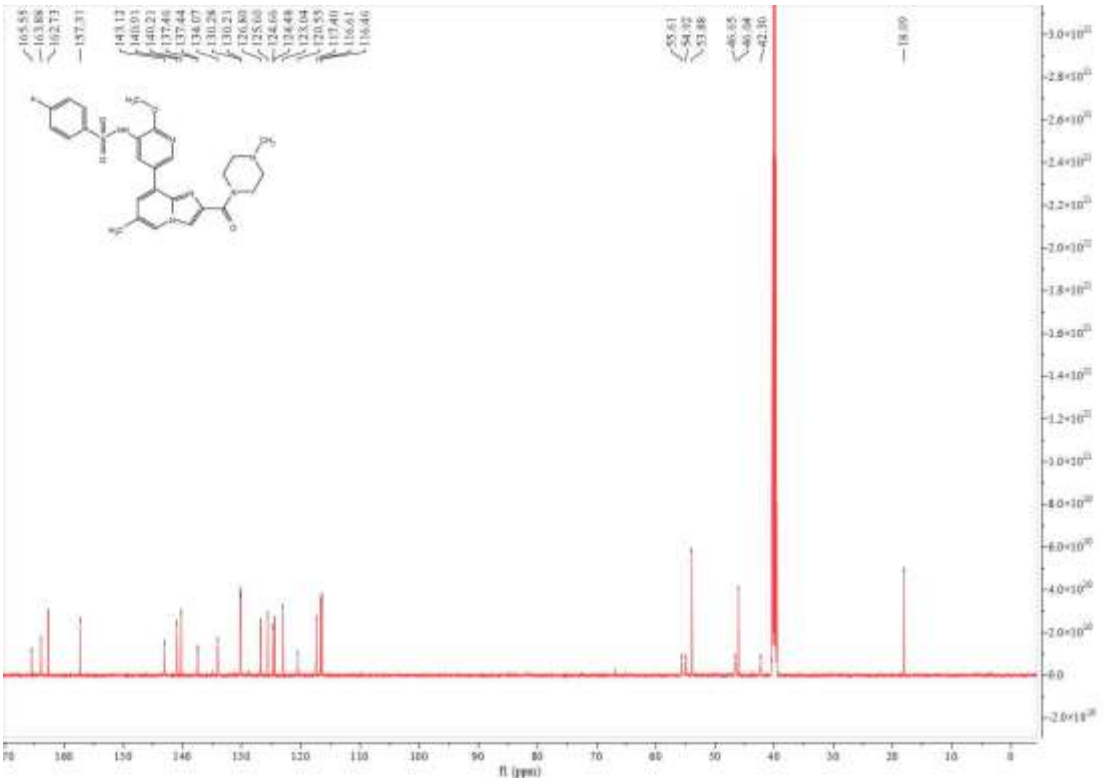

Figure S46. ^{13}C-NMR spectrum of compound **44.**

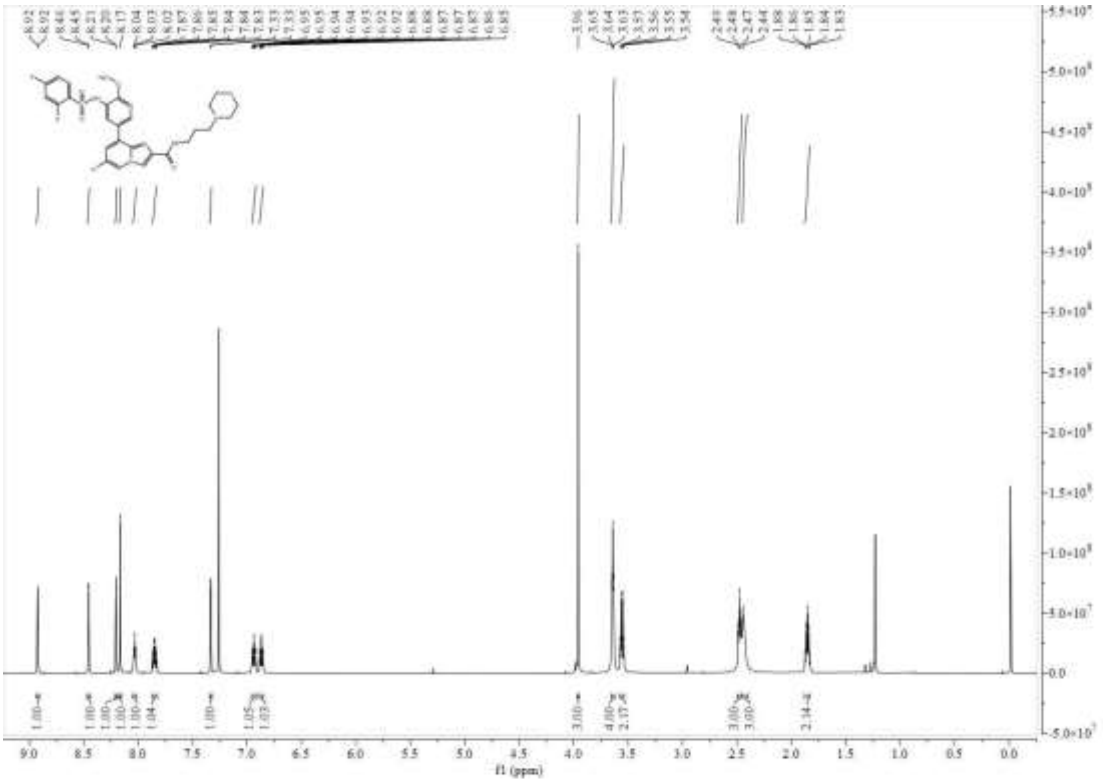

Figure S47. ^{1}H-NMR spectrum of compound **45.**

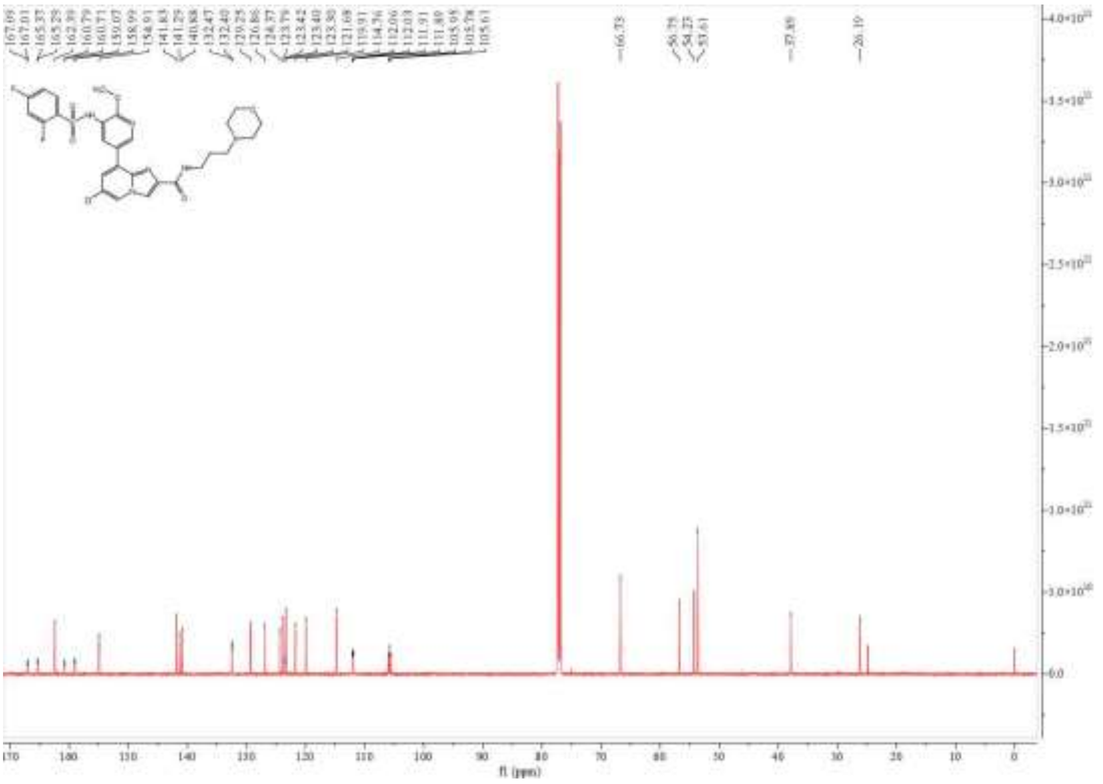

Figure S48. ^{13}C-NMR spectrum of compound **45.**

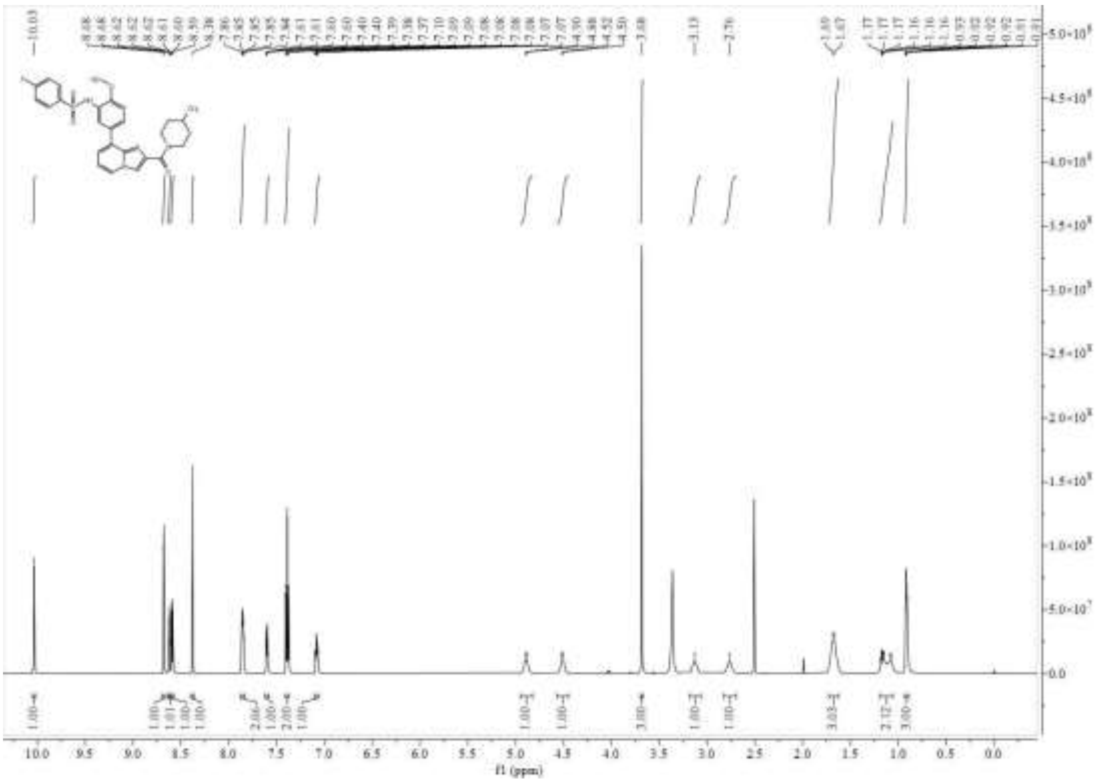

Figure S49. ^{1}H-NMR spectrum of compound **46.**

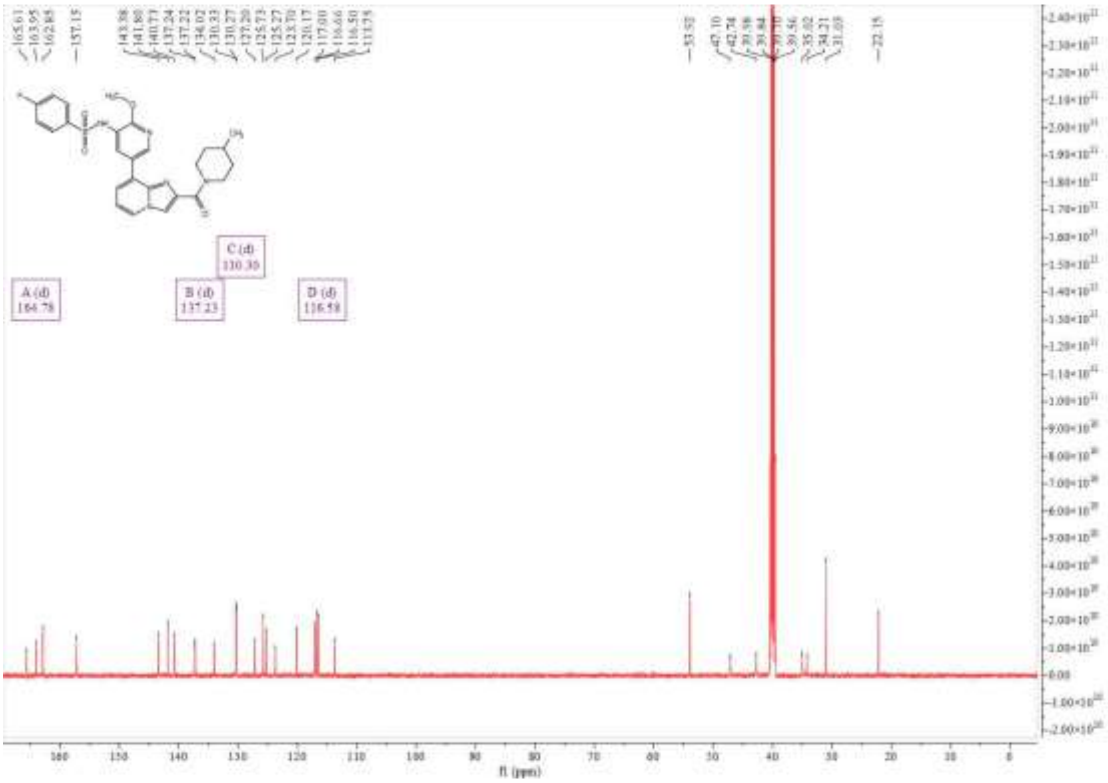

Figure S50. ^{13}C-NMR spectrum of compound **46.**
